# A fast risk assessment method for power balance under extreme weather conditions based on deep reinforcement learning with a coupled attention mechanism

**DOI:** 10.1371/journal.pone.0359101

**Published:** 2026-10-08

**Authors:** Wang Hang, Sun Shouheng, Li Yiming, Li Baoju, Cong Yu, Xu Meiyue, HeXiang Niu

**Affiliations:** 1 School of Energy and Power Engineering, Northeast Electric Power University, Jilin, China; 2 Electric Power Research Institute of State Grid Jilin Electric Power Co., Ltd., Changchun, China; 3 State Grid Jilin Electric Power Co., Ltd., Changchun, China; Aalto University, FINLAND

## Abstract

Even if persistent stagnant weather does not directly damage power grid equipment, it may lead to prolonged insufficient output of renewable energy sources and further trigger power balance risks. This paper proposes a rapid risk assessment framework that comprehensively incorporates meteorological sensitivity models, time-varying component failure probabilities, a Denoising Variational Autoencoder (DVAE) for generating coupled wind-photovoltaic-temperature scenarios, and a Deep Deterministic Policy Gradient (DDPG) model embedded with the feature token attention mechanism.The 72-hour assessment task is formulated as an offline Markov Decision Process (MDP): the state space covers meteorological conditions, power sources, power grid topology, load profiles, and risk features of the previous time step; continuous actions are defined as risk indicator vectors; the reward function is designed to penalize deviations between model outputs and PSD-BPA benchmarks, temporal inconsistency, and violations of physical constraints.To distinguish the sequential reinforcement learning formulation from direct regression, a feedforward Multi-Layer Perceptron (MLP) is introduced as the non-reinforcement learning baseline. Four metrics, namely Mean Absolute Error (MAE), Root Mean Square Error (RMSE), coefficient of determination (R^2^), and the 95th percentile of absolute error (P95AE), are adopted to evaluate all models on an independent scenario-level test set.Verified on the planned power grid of Northeast China in 2025 as the test case, the proposed DDPG-Attention achieves MAE of 0.0310, RMSE of 0.0389, R^2^ of 0.954 and P95AE of 0.0760 on the independent test set. The average time consumption for a single risk assessment is approximately 0.12 seconds, yielding an acceleration ratio of multiple orders of magnitude compared with the traditional time-sequential Monte Carlo simulation based on PSD-BPA. This study provides an efficient and accurate novel technical paradigm for the rapid evaluation of power supply-demand imbalance risks in power systems under extreme weather conditions.

## 1. Introduction

With the in depth advancement of the “dual carbon” goals, the construction of new power systems has entered a critical stage, and the share of renewable energy installations, represented by wind power and photovoltaics (PV), continues to increase [1,2]. However, the strong intermittency and volatility of renewable energy output make the system operation significantly more dependent on meteorological conditions. Unlike highly destructive extreme weather events such as typhoons and thunderstorms, persistent stagnant weather does not directly damage the physical structure of the power grid, but its long duration and wide impact range can easily lead to renewable energy being in a state of low or even zero output for an extended period, thereby triggering long term power supply demand imbalance risks in the power system. Events such as high temperature droughts and multiple days without wind or sunlight that have occurred in many regions worldwide have fully demonstrated that weather factors are becoming an important unknown risk source threatening the security of power supply in new power systems [3,4]. Therefore, studying the supply demand imbalance risk of power systems under extreme weather and developing fast and accurate risk assessment methods are of significant theoretical importance and engineering value.

Currently, domestic and international scholars have conducted extensive research on power system risk assessment under extreme weather. In terms of source load characteristic modeling, references [5,6] proposed renewable energy station output prediction methods considering factors such as wind speed, temperature, humidity, and solar irradiance. Reference [7] separated climate sensitive load from total load by analyzing the spatiotemporal correlation between climate factors and power load. Reference [8] studied the impact of high temperature and drought weather on the output of hydropower and thermal power, and proposed elasticity indicators at the power source level. In the field of system resilience assessment, existing studies have proposed static resilience indicators based on resource adequacy and component redundancy [9,10], as well as dynamic resilience indicators established according to the recovery process during and after extreme events. Regarding scenario generation, reference [11] proposed a weather scenario construction method based on extreme value theory, but it lacked specific scenario modeling and case study validation. References [12,13] explored the emergency control strategies of power systems and the optimal configuration of distributed generation and energy storage devices under different scenarios.

However, existing research still has obvious limitations in coping with supply demand imbalance risks of power systems under extreme weather. On the one hand, most studies tend to analyze sudden extreme weather events and persistent stagnant weather separately, failing to uniformly reveal the differentiated impact mechanisms of the two types of extreme weather from the three aspects of supply, transmission, and demand. On the other hand, at the uncertainty modeling level, source side output fluctuations, grid side equipment failures, and load side climate sensitive responses are often treated in isolation, and a comprehensive quantification of their coupled uncertainties is lacking. In particular, the time varying characteristics of equipment failure probability, which are jointly affected by service life, environmental conditions, and operating status, are insufficiently considered in existing risk assessment models. More critically, although traditional time series production simulation or Monte Carlo methods can obtain relatively accurate risk results, their huge computational overhead makes it difficult to meet the timeliness requirements for rapid risk identification during the evolution of extreme weather. Moreover, existing resilience assessment studies mostly focus on short term emergency power supply after the destruction of grid topology by extreme weather, while the exploration of rapid assessment methods for global and persistent supply demand imbalance risks remains insufficient. The above deficiencies indicate that there is an urgent need to develop an intelligent method that can efficiently handle high dimensional state spaces, adaptively focus on key risk features, and combine offline training with online rapid assessment.

Deep Learning (DL), Active Learning (AL) and Deep Reinforcement Learning (DRL) address different types of methodological problems and should not be conflated. Supervised deep learning learns a direct mapping from labeled inputs to outputs; active learning selects highly informative samples from unlabeled data for supplementary labeling; deep reinforcement learning derives optimal policies through state transitions and reward feedback. Existing power system studies based on deep learning or deep active/transfer learning are mainly applied to transient stability classification or regression tasks to lower the cost of label acquisition [14–20]. Such methods are suitable when each operating point is processed independently. In contrast, the problem investigated in this paper involves a 72-hour evolutionary process, in which meteorological conditions, equipment states and risk indicators are temporally coupled. Accordingly, the DDPG algorithm [21] that supports Actor–Critic learning with continuous actions and sequential state spaces is adopted in this work. Meanwhile, given that PSD-BPA labels can be batch-generated via offline simulations, direct supervised regression serves as a reliable alternative for this task. To quantify the performance gain brought by the temporal dependency modeling within the reinforcement learning framework compared with static supervised mapping, a Multi-Layer Perceptron (MLP) is added as a comparative baseline in this paper. Unified comparisons in terms of prediction accuracy and computational efficiency are conducted between the proposed DDPG-Attention method and the MLP on the same independent test set. Table 1 categorizes and compares mainstream methodological paradigms in power system risk assessment, including their scenario sources, modeling characteristics, core limitations, and the roles adopted in this work.

**Table 1:** Distinguishes the contributions of this study from the most relevant methodological categories.

Method Category	Scenario/ Label Source	Learning & Modeling Paradigm	Main Limitations Compared with the Proposed Method	Role in This Work
Temporal or Monte Carlo Risk Assessment [24,25]	Physics-based simulation/ PSD-BPA	Learning-free; scenario-by-scenario computation	Excessively high online computational overhead	Label generator and numerical benchmark
DVAE Scenario Generation [22]	Historical wind, photovoltaic and temperature data	Unsupervised generative model	Incapable of risk inference or source-grid-load fault coupling	Adopted as the referenced scenario generation module
Supervised/ Active/ Transfer Learning [14–20]	Labeled operating points or selectively queried samples	Direct classification or regression	Samples are processed independently without sequential reward optimization	Used for demonstration and construction of the MLP baseline
Vanilla DDPG [21] & Scaled Dot-Product Attention [23]	General environmental interaction	Attention-augmented Actor–Critic for continuous actions	Lack of domain-specific risk states, reward design and coupling with PSD-BPA labels	Adopted as the fundamental algorithm modules
Proposed Method	DVAE-generated scenarios + time-varying failures + PSD-BPA risk labels	Offline sequential MDP with continuous multi-risk actions	–	Newly proposed integrated modeling, reward function, feature token attention and evaluation protocol

To remedy the above limitations, this paper proposes a rapid risk assessment framework for power balance under extreme weather scenarios. The inherited modules from existing literature include the DVAE scenario generator [22], DDPG Actor–Critic architecture [21], and scaled dot-product attention mechanism [23]. The original contributions of this work are summarized as four aspects: (1) Construction of a unified source-grid-load uncertainty chain that connects meteorological sensitivity, time-varying component failures and time-series risk labels from PSD-BPA; (2) Formulation of an offline sequential MDP with continuous actions defined as multi-dimensional risk indicator vectors, where the reward function is explicitly correlated with PSD-BPA prediction errors as well as temporal and physical constraint consistency; (3) Design of a feature token attention layer targeting feature groups of meteorology, power sources, power grid topology, load profiles and historical risks; (4) Establishment of a comprehensive evaluation protocol covering the supervised MLP baseline, scenario-level training/validation/test set partition, independent test error metrics, and reproducible real-time runtime measurement.

## 2. Analysis on multidimensional impacts of extreme meteorology on supply-transmission-demand of power system

### 2.1. Qualitative analysis of supply-transmission-demand in power system under extreme meteorological conditions

The impact of extreme weather events on power systems is becoming increasingly significant, particularly in the context of a high proportion of renewable energy integration, where system dependence on meteorological conditions is markedly enhanced. Unlike conventional meteorological fluctuations, extreme weather is characterized by suddenness, persistence, and strong destructiveness, and can simultaneously impact the supply capacity, transmission security, and load characteristics of power systems from multiple dimensions, thereby triggering risks of power supply–demand imbalance. Based on the deep coupling relationship between meteorology and power systems, this paper qualitatively analyzes the influence mechanism of extreme weather from three aspects: supply, transmission, and demand.

On the power supply side, the impact of extreme weather on renewable energy generation capacity manifests as two typical processes. Sudden extreme weather events, such as typhoons, blizzards, and thunderstorms, are often accompanied by strong winds, low temperatures, or heavy precipitation, directly causing damage to generation equipment or shutdowns. Typhoons can cause wind turbine tower collapse or blade fracture; blizzards and freezing rain may lead to ice coating on photovoltaic (PV) panels, shading sunlight and significantly reducing PV conversion efficiency; and cold waves may cause brittle fracture of mechanical components or lubricant failure in wind turbines, thereby substantially reducing wind power output. In addition, extreme precipitation or drought can alter reservoir inflow conditions, limiting hydroelectric generation capacity. Another type is persistent stagnant weather, such as stagnant haze and low sunlight. Although not violently destructive, its long duration and wide influence range can easily lead to wind and PV power remaining at low or even zero output for extended periods. Under stagnant weather, low wind speeds limit wind power output, while haze reduces solar radiation intensity, further suppressing PV generation, creating a “low wind and low sunlight” scenario, which in turn leads to longterm supply–demand imbalances in the power system.

Power transmission systems also face severe challenges under extreme weather conditions. Extreme weather events such as wind, rain, ice, and snow can directly act on transmission lines, towers, insulators, and other equipment, compromising the integrity of the grid structure and causing transmission channel interruptions or electrical faults. Typhoons and strong winds easily lead to windinduced conductor flashovers, tower structure damage, and even tripping due to short circuits caused by external floating objects on highvoltage electrical equipment. Cold waves and blizzards can cause line icing, which in severe cases may lead to conductor breakage and tower collapse, resulting in largescale blackouts. Thunderstorms may cause lightning flashovers on substations and transmission lines, damaging insulation components and triggering frequency excursions or voltage instability. These transmissionside failures not only weaken crossregional power allocation capabilities but also exacerbate regional supply–demand contradictions, especially when transmission corridors for renewable energy are constrained, further amplifying power balance risks.

On the load side, under extreme weather conditions, electricity demand exhibits distinctly nonlinear response characteristics. Extremely high temperatures lead to massive activation of air conditioners and cooling equipment, causing a sharp increase in load; extremely low temperatures trigger concentrated release of heating loads such as electric heating and water heaters. Meanwhile, distributed generation and electric vehicles may also experience power supply interruptions or concentrated charging demand outbreaks under extreme weather, further disturbing load profiles.

The impacts of extreme weather on the three dimensions of power supply, transmission, and demand are not isolated but rather coupled and amplified. While extreme weather reduces renewable energy output, it may simultaneously cause transmission line tripping. If, at the same time, load increases due to heating or cooling demand, the system will face multiple shocks, easily triggering cascading events such as frequency excursions and regional blackouts. To systematically summarize the influence mechanisms of different types of extreme weather on the source–grid–load of power systems, Fig 1 categorizes, from three time scales (ultra short term, short term, and medium to–long term), the specific impact pathways of extreme weather events (including thunderstorms, dust storms, typhoons, blizzards, droughts, and high temperatures) on source-side equipment, grid-side structures, and load-side demand. It reveals the systemic mechanism by which extreme weather ultimately affects power balance through the chain of “equipment damage transmission interruption load fluctuation”, thereby providing a theoretical basis for subsequent quantitative analysis and risk modeling.

**Fig 1:**
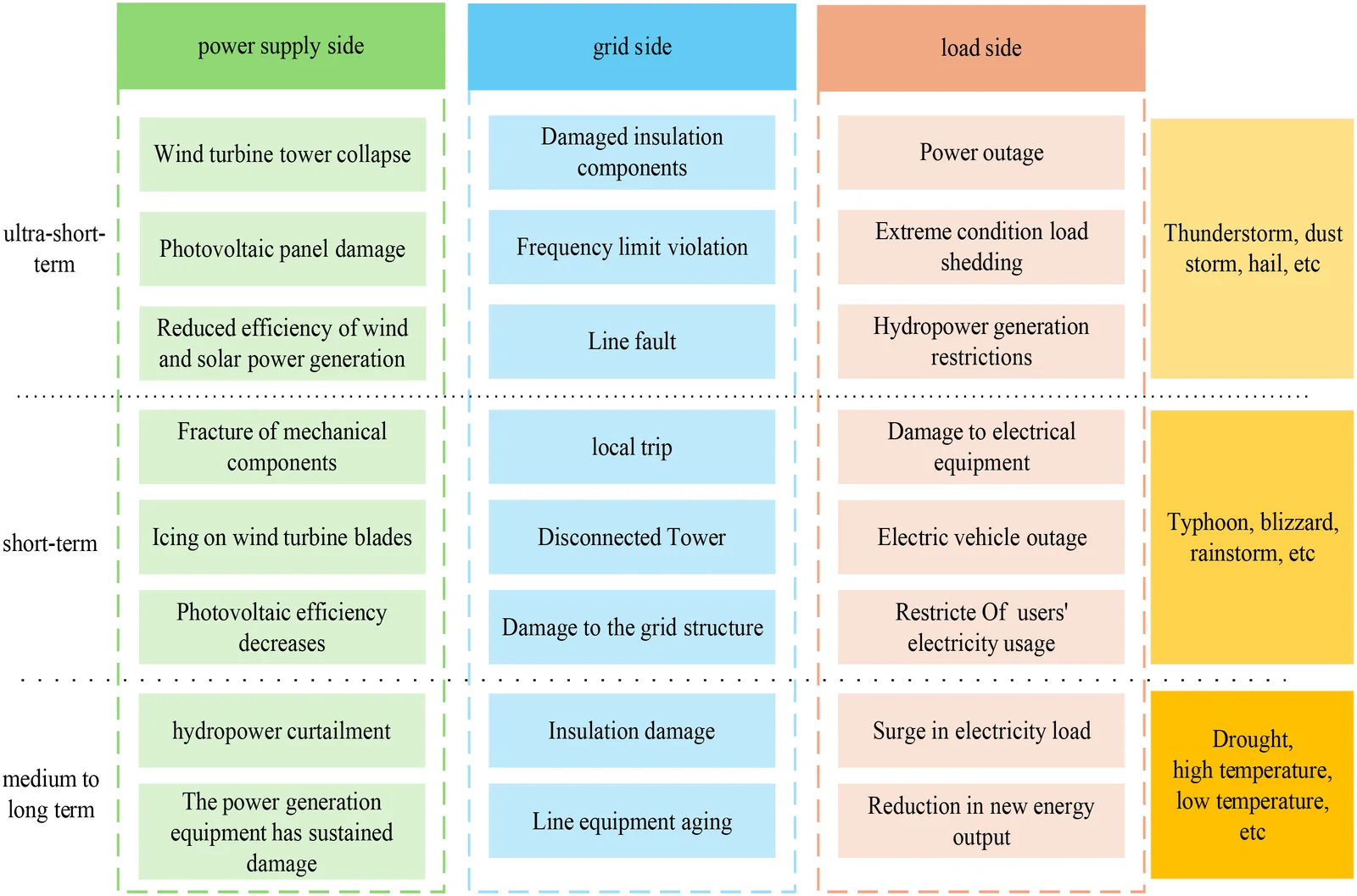
Extreme weather typing and its impact on source-grid-load of power svstem.

### 2.2. Construction f meteorological sensitivity model for source-grid-load in power system

After completing the qualitative analysis of the influence mechanisms of extreme weather, these mechanisms are further transformed into mathematical models that are computable and embeddable into the risk assessment process. Based on the key meteorological parameter sets that affect renewable energy station output, transmission capacity, and climatesensitive loads, this paper constructs meteorological sensitivity models for wind power, photovoltaic (PV) power, hydropower, transmission lines, and loads, respectively. In doing so, the “influence pathways” described above are quantified into input variables required for subsequent scenario generation and risk calculation.

#### 2.2.1. Wind turbine unit.

Wind turbines convert wind energy into electrical energy through rotors and synchronous generators. The output power depends on the realtime wind speed and the power characteristic curve of the turbine, and is calculated using Eq. (1) [26].


$$\begin{array}{c} P_{w,t}=min\left(\frac{\text{1}}{\text{2}}\rho_{a}AC_{p}w_{t},\text{\textbackslash{}hspace\{0.33em}\}\frac{\text{1}}{\text{2}}\rho_{a}AC_{p}w_{n}^{\text{3}}\right)\; \end{array}$$ (1)

In the formula: $P_{w,t}$ is the realtime output of the wind turbine at time t; $\rho_{a}$ is the air density; A is the swept area; $C_{p}$ is the wind turbine efficiency coefficient; $w_{t}$ is the wind speed at time t; $w_{n}$ is the wind speed at the rated power of the wind turbine. Among them, $\rho_{a}$ decreases with increasing temperature. When the temperature rises from 0°C to 40°C, $\rho_{a}$ decreases from 1.29 kg/m³ to 1.08 kg/m³.

#### 2.2.2. Photovoltaic power generation system.

The output power of a photovoltaic (PV) power generation system varies proportionally with solar irradiance, while temperature also affects the power generation efficiency of solar cells. Assuming that the control strategy of the PV inverter ensures that the PV modules operate at the maximum power point, the output power is proportional to the solar irradiance within the normal range. Within the rated power range, the output power of a PV module is proportional to the normal incident irradiance on the PV panel [27]. as shown in Eq. (2).


$$\begin{array}{c} P_{PV,t}=min\left\{\frac{\text{1}}{1000}r_{t}P_{PVn}\left[1-0.005(\theta_{a,t}-25^{\circ }C)\right],P_{PVn}\right\} \end{array}$$ (2)

In the formula: $P_{PV,t}$ is the realtime output of the photovoltaic power generation system at time t; $r_{t}$ is the irradiance at time t; $\theta_{a,t}$ is the ambient temperature at time t; $P_{PV,t}$ is the output of the photovoltaic power generation system under standard test conditions.

For example, when the operating temperature of a PV panel reaches 55°C, the output power under the same irradiance is reduced by 15% compared to that at 25°C.

#### 2.2.3. Hydropower unit.

The output power of a hydropower unit is determined jointly by the water flow rate and the water pressure, both of which are related to the reservoir water level. Over a relatively long period, the water level is closely associated with weather conditions and flood discharge scheduling operations. When persistent hot and dry weather causes significant evaporation and a consequent drop in the reservoir water level, the output power of the hydropower unit decreases. The approximate output power of the hydropo wer unit is determined by Eq. (3) [8].


$$\begin{array}{c} P_{H,t}=min\left(1\times 10^{-6}\eta_{H}Q_{tb}\rho_{w}gH_{net},P_{Hn}\right)\; \end{array}$$ (3)

In the formula: $P_{H,t}$ is the realtime output of the hydropower unit at time t; $\eta_{H}$ is the generation efficiency of the hydropower unit; $Q_{tb}$ is the water flow velocity through the turbine; $\rho_{w}$ is the density of water; g is the gravitational acceleration; $H_{net}$ is the elevation of the water level relative to the turbine, also known as the hydraulic head; $P_{Hn}$ is the rated output of the hydropower unit.

The increment in reservoir water level over a given time period ΔT is equal to the integral of the net inflow, involving five parameters: the upstream inflow velocity $Q_{in}$, the surface evaporation rate $Q_{eva}$, the bottom seepage rate $Q_{leak}$, the flood discharge velocity Qout, and the water flow velocity through the turbine Qtb. The expression for $H_{net}$ is given by Eq. (4) [28, 29].


$$\begin{array}{c} H_{\text{net}}=H_{\text{net0}}+\int_{\text{0}}^{\Delta T}f_{\text{Re}}(Q_{\text{in}}-Q_{\text{eva}}-Q_{\text{leak}}-Q_{\text{out}}-Q_{\text{tb}})\;dt \end{array}$$ (4)

$Q_{leak}$ is related to the geological conditions of the reservoir and is largely unaffected by weather; Qout is determined by the scheduling decisions of the water resources department; $Q_{eva}$ is significantly influenced by climatic factors. Due to the irregularity of the reservoir’s slope surface, the function $f_{\text{Re}}$ is nonlinear. When the change in reservoir water level is small, it can be approximately considered that the slope surface is vertical. Under this assumption, if the various flow parameters are constant, Eq. (4) can be approximated as Eq. (5).


$$\begin{array}{c} H_{\text{net}}\approx H_{\text{net0}}+\frac{(Q_{\text{in}}-Q_{\text{eva}}-Q_{\text{leak}}-Q_{\text{out}}-Q_{\text{tb}})\Delta T}{A_{\text{res}}} \end{array}$$ (5)

In the formula: $A_{\text{res}}$ is the reservoir area.

Generally, $Q_{eva}$ is mainly influenced by factors such as the water vapor pressure difference Δe, the water vapor temperature difference Δθ, the relative air humidity h, and the wind speed above the water surface $w_{\text{1}}$. If the influences of these four factors are decoupled, $Q_{eva}$ can be simulated using the empirical formula given in Eq. (6) [30].


$$\begin{array}{c} Q_{eva}=\Delta ef_{H}\left(\Delta \theta \right)f_{M}\left(h\right)f_{W}\left(w_{\text{1}}\right)\; \end{array}$$ (6)

In the formula: $f_{H}$ (Δθ) is the function of the influence of water vapor temperature difference; $f_{M}\left(h\right)$ is the function of the influence of relative humidity; $f_{W}\left(w_{\text{1}}\right)$ is the function of the influence of wind speed.

Based on observation data from multiple reservoirs over a long timescale, three functions can be fitted using statistical methods. The statistical model shows that when the water vapor temperature difference increases from 0°C to 10°C, the evaporation rate increases by approximately 45%. Therefore, under the condition that $Q_{in}$ and Qout are constant, temperature is the main factor affecting the reservoir water level. Furthermore, high temperatures and drought weather in summer lead to a decrease in relative air humidity, which increases $f_{H}\left(\Delta \theta \right)$ and further reduces the reservoir water level [30].

#### 2.2.4. Transmission line capacity model.

The available transfer capacity also referred to as the thermal stability limit) of a transmission line is influenced by the ambient temperature. Based on the heating and heat dissipation mechanisms of conductors, the transferable capacity of an overhead line (i,j), denoted as ${\overset{―}{P}}_{ij}\left(\theta_{a,t}\right)$, can be estimated using Eq. (7) [31].where *i* and *j* are the numbers of the sendingend and receivingend nodes of transmission line l, respectively.


$$\begin{array}{c} {\overset{―}{P}}_{ij}\left(\theta_{a,t}\right)=\sqrt{\frac{9.92(w_{l}D{)}^{0.5}+\alpha_{\text{1}}[(\Delta \theta_{l}+\theta_{a,t}{)}^{\text{4}}-\theta_{a,t}^{\text{4}}]-\alpha_{\text{2}}D}{R_{l}}} \end{array}$$ (7)

In the formula: $R_{l}$ is the AC resistance of transmission line l at 0°C; $w_{l}$ is the wind speed above transmission line l; D is the outer diameter of the conductor; $\Delta \theta_{l}$ is the average temperature rise on the surface of transmission line l; $\alpha_{\text{1}},\alpha_{\text{2}}$ are parameters related to the physical characteristics of the conductor.

#### 2.2.5. Climate-sensitive load.

The composition of load is relatively complex, and there are multiple classification methods. According to user category, load can be divided into industrial, commercial, residential, and public service loads. From the perspective of emergency power protection under extreme weather conditions, the substation node load ${\text{P}}_{\text{D,t}}$ at time t is divided into rigid load ${\text{P}}_{\text{D1,t}}$, elastic load ${\text{P}}_{\text{D2,t}}$, and interruptible load ${\text{P}}_{\text{D3,t}}$, See Eq. (8) for an example:.


$$\begin{array}{c} {\text{P}}_{\text{D,t}}{\text{= P}}_{\text{D1,t}}{\text{+P}}_{\text{D2,t}}{\text{+P}}_{\text{D3,t}} \end{array}$$ (8)

Among these, the rigid load represents the load essential for ensuring social production and daily life and is generally noninterruptible. The elastic load represents the load that can be curtailed through demandside response under extremely hot or extremely cold weather conditions. The interruptible load represents the load that can be completely shed when power supply is insufficient, including certain industrial and commercial loads [32], for which the amount to be shed can be determined according to the severity of the power shortage, thereby alleviating the imbalance between supply and demand.

In this paper, elastic load is defined as climate sensitive load, which mainly consists of residential cooling and heating loads. In recent years, with economic and social development, industrial structure adjustment, and improvement in people’s living standards, the proportion of climate sensitive load in total electricity load has been increasing. In several provincial power grids, the summer cooling load accounts for more than 40% of the peak electricity load. Taking air conditioners and electric heating as examples, the relationship between load and ambient temperature can be estimated using Eq. (9) [33].


$$\begin{array}{c} \left\{ \begin{array}{c} @l@P_{D_{Co,t}}=\frac{N_{AC}(\theta_{a,t}-\theta_{c})}{\eta_{c}R_{AC}}\\ P_{D_{Hea,t}}=\frac{N_{AC}(\theta_{H}-\theta_{a,t})}{\eta_{H}R_{AC}} \end{array}\right.\; \end{array}$$ (9)

In the formula: $P_{D_{Co,t}}$ and $P_{D_{Hea,t}}$ are the power of cooling air conditioners and heating systems at time t under different temperatures, respectively; $N_{AC}$ is the number of users; $\theta_{c}$ and $\theta_{H}$ are the average set temperatures for cooling and heating of the air conditioner cluster, respectively; $R_{AC}$ is the average thermal resistance of the houses where the air conditioner cluster is located, which is determined by the house area, the position of the house in the building, and the exterior material of the building; $\eta_{c}$ and $\eta_{H}$ are the average thermal efficiencies for cooling and heating, respectively.

In extremely hot weather, orderly power utilization measures can be implemented to raise the set temperature of user air conditioners, thereby reducing air conditioning load. For example, a large sample questionnaire survey on incentive based demand response in two U.S. states showed that user participation in demand response programs increases with the monthly compensation amount. Among these, a monthly compensation plan of $10 includes a series of load control contracts, such as raising the air conditioner set temperature by 3°C during extremely hot weather (maximum temperature > 38°C). According to the survey results, approximately 40% of users were willing to enroll in this plan [33]. In fact, orderly power utilization under extreme weather differs from that under normal operating scenarios. In China, the regulation depth of air conditioning and electric heating loads can be further enhanced [34] through policy guidance. Based on Eq. (9), the reduction in temperature controlled load can be estimated using Eq. (10).


$$\begin{array}{c} \Delta P_{D2,t}=\frac{N_{AC}\left|\Delta \theta_{AC}\right|}{\eta R_{AC}} \end{array}$$ (10)

In the formula: $\Delta P_{D2,t}$ is the load shedding of thermostatically controlled loads at time t; η is the average thermal efficiency, which can be $\eta_{C}$ or $\eta_{H}$; $\Delta \theta_{AC}$ is the difference between ambient temperature and load temperature.

When $\Delta \theta_{AC}$ takes its upper limit $\Delta {\overset{―}{\theta }}_{AC}$, the upper limit of load shedding is $\Delta {\overset{―}{P}}_{D2,t}$.

## 3. Uncertainty probability modeling and generation of system risk scenarios based on extreme meteorology

Based on the meteorological sensitivity models, this chapter further quantifies the occurrence probabilities of extreme weather events and establishes a time varying fault probability model that comprehensively considers equipment service life, environmental conditions, and operating states. Finally, a denoising variational autoencoder (DVAE) is introduced to generate risk scenarios with complex coupling characteristics, providing a reasonable scenario set for assessing the risk of supply–demand imbalance.

### 3.1. Extreme meteorological occurrence probability model

The inverse transform method can generate random variables that follow a given probability distribution [35]. Using this method to generate meteorological factors under corresponding extreme weather conditions in risk scenarios, the specific implementation steps are as follows:

1) From historical meteorological data, fit the probability density function, cumulative distribution function, and its inverse function of the meteorological factor $x_{i}^{\left(h\right)}$, denoted as

$f\left(x_{i}^{\left(h\right)}\right),F\left(x_{i}^{\left(h\right)}\right)$ and $F^{-1}\left(x_{i}^{\left(h\right)}\right)$ respectively, where h=1,2,3;

2) Generate n random variables  R uniformly distributed on (0,1), substitute each into the inverse probability distribution function, and the obtained $F^{-1}\left(R\right)$ gives the values of the meteorological factors, i.e., $\hat{X}=(F^{-1}(R){)}_{n\times n}$;
3) Randomly select $T^{\prime }$ sets of values from $\hat{X}$ that satisfy the extreme meteorological condition expression as the meteorological factors X for the corresponding risk scenarios.

$$\begin{array}{c} p_{s,x}=\prod_{h=1}^{\text{3}}p_{s,x_{t}^{\left(h\right)}} \end{array}$$ (11)

In the formula: $x_{t}^{\left(h\right)}$ is the type of meteorological factor at time t; $p_{s,x_{t}}$ is the probability of occurrence of the type of meteorological factor at time t under risk scenario s. This probability can be obtained by integrating the probability density function $f\left(x_{t}^{\left(h\right)}\right)$ of the meteorological factor $x_{t}^{\left(h\right)}$ over the value interval corresponding to extreme meteorological conditions, See Eq. (12) for an example:


$$\begin{array}{c} p_{s,x_{t}^{\left(h\right)}}=\left\{ \begin{array}{cc} @ll@\int_{(1-\sigma )\phi_{t}^{\left(h\right)}}^{+\infty }f\left(x_{t}^{\left(h\right)}\right)\;dx_{t}^{\left(h\right)} & x_{t}^{\left(h\right)}\geq (1-\sigma )\phi_{t}^{\left(h\right)}\\ \int_{-\infty }^{(1+\sigma )\phi_{t}^{\left(h\right)}}f\left(x_{t}^{\left(h\right)}\right)\;dx_{t}^{\left(h\right)} & x_{t}^{\left(h\right)}\leq (1+\sigma )\phi_{t}^{\left(h\right)} \end{array}\right. \end{array}$$ (12)

In the formula: $\phi_{t}^{\left(h\right)}$ is the extreme boundary value of the power grid state related to the meteorological factor $x_{t}^{\left(h\right)}$.Taking $\phi_{t}^{\left(1\right)}$ as an example, it can be expressed as $\phi_{t}^{\left(1\right)}$ = $\left\{\Delta P^{L,pred}\left(y_{t}^{\left(2\right)}\;\right),\theta^{line,rate}\left(y_{t}^{\left(3\right)}\;\right),\theta^{transf,rate}\left(y_{t}^{\left(3\right)}\;\right)\right\}$.

### 3.2. System equipment failure probability model

After obtaining the probability distribution of meteorological factors using the inverse transform method, the occurrence probability of extreme weather events can be further calculated. However, knowing the meteorological conditions alone is insufficient for assessing system risk; it is also necessary to determine the equipment failure probability under such weather conditions. Under extreme weather, the equipment failure probability in the planning grid exhibits time varying characteristics due to the combined effects of long term service life, short term external environment, and operating states. To accurately reflect the time varying equipment failure probability, the above three types of influencing factors are comprehensively considered, and a comprehensive time varying failure probability model is established for various types of equipment, including transmission lines, transformers, and generators.

1) Basic equipment failure probability

The basic failure rate of equipment follows a bathtub curve with increasing service life and rises sharply during the wearout failure period. Since a large number of equipment units in the planning grid may be in this period, the service life factor is taken into account. It is assumed that the basic failure rate of equipment remains constant during the assessment period. The bathtub curve and an exponential model are used to describe the basic failure rate and basic failure probability of all types of equipment, See Eqs. (13) and (14) for an example:


$$\begin{array}{c} \lambda_{j,t}^{\text{base}}=\frac{\beta_{j}}{\alpha_{j}}{\left(\frac{T_{j}^{\text{0}}}{\alpha_{j}}\right)}^{\beta_{j}-1} \end{array}$$ (13)

$$\begin{array}{c} p_{j,t}^{\text{base}}=1-exp\left(-\sum_{t\in \Omega^{T}}\lambda_{j,t}^{\text{base}}\Delta t\right)=1-exp\left(-\lambda_{j,t}^{\text{base}}T\right)\; \end{array}$$ (14)

In the formula: $T_{j}^{\text{0}}$ is the service life of equipment *j*; $\lambda_{j,t}^{\text{base}}$ are the base failure rate and base failure probability of equipment *j* at time *t*, respectively; $\alpha_{j}$, $\beta_{j}$ are the life parameter and shape parameter of equipment *j*, respectively; Δt is the *t*ime step.

2) Environmentdependent equipment failure probability

At the environmental level, different types of extreme weather have different influence mechanisms on different equipment. Under continuous high temperature weather, when the hotspot temperature of equipment is excessively high, the tensile life of lines and the insulation life of transformers are significantly shortened, leading to accelerated equipment aging. Under blizzard weather, transmission lines experience icing and bear the dual mechanical effects of wind and ice loads. When the force on the lines exceeds the mechanical limit as ice thickness increases, metal deformation or even conductor breakage may occur. Therefore, in this section, a temperature-condition-dependent equipment failure probability model and an ice-and-wind-load-dependent line failure probability model are established, respectively.

(1) Temperature-condition-dependent equipment failure probability model

The relationships between the service life and the operating temperature of transmission lines and transformers see Eqs. (15) and (16) for an example:


$$\begin{array}{c} \alpha_{j,t}^{\text{line}}=\chi exp(-\gamma x_{j,t}^{\left(1\right),\text{line}}) \end{array}$$ (15)

$$\begin{array}{c} \alpha_{j,t}^{\text{trf}}=fexp\left(\frac{g}{x_{j,t}^{\left(1\right),\text{trf}}+273}\right) \end{array}$$ (16)

In the formula: $\alpha_{j,t}^{\text{line}}$ and $\alpha_{j,t}^{\text{trf}}$ are the operational lifetimes of line *j* and transformer *j* at time *t*, respectively; $x_{j,t}^{\left(1\right),\text{trf}}$ and $x_{j,t}^{\left(1\right),\text{line}}$ are the operating temperatures of line *j* and transformer *j* at *t*ime *t*, respectively, which can be calculated based on the ambient temperature $x_{t}^{\left(1\right)}$; γ is a coefficient; γ = b/m where b is a constant related to the properties of the conductor ma*t*erial, and m is the mass of the line; *f* and *g* are empirical constants.

Substituting the equipment life parameters $\alpha_{j,t}^{\text{line}}$ and $\alpha_{j,t}^{\text{trf}}$ into Eq. (13) yields the temperature-condition-dependent line failure rate $\lambda_{j,t}^{dis,line}$ and transformer failure rate $\lambda_{j,t}^{dis,trf}$, respectively. Similarly, the exponential model is used to describe the failure probability, See Eqs. (17)–(18) for an example:


$$\begin{array}{c} p_{j,t}^{\text{dis, line}}=1-exp\left(-\sum_{t\in \Omega^{T}}\lambda_{j,t}^{\text{dis, line}}\Delta t\right) \end{array}$$ (17)

$$\begin{array}{c} p_{j,t}^{\text{dis, trf}}=1-exp\left(-\sum_{t\in \Omega^{T}}\lambda_{j,t}^{\text{dis, trf}}\Delta t\right)\; \end{array}$$ (18)

In the formula: $p_{j,t}^{\text{dis, line}}$ and $p_{j,t}^{\text{dis, trf}}$ are the failure probabilities of line *j* and transformer *j* affected by extreme weather at time *t* respectively.

(2) Ice-and-wind-load-dependent line failure probability model

Since the amount of snow accretion on a line is related to its geographical location, a geographically dependent line icing growth model is first established by considering the meteorological factors of wind speed, temperature, and snowfall, See Eqs. (19)–(21) for an example:


$$\begin{array}{c} x_{j,t}^{\left(3\right),\text{line}}=\int_{\text{0}}^{t}v_{j,\tau }^{\text{ice}}d\tau \end{array}$$ (19)

$$\begin{array}{c} v_{j,t}^{\text{ice}}=\sqrt{(v_{j,t}^{\text{snow}}\rho {)}^{\text{2}}+{\left[0.26k_{\text{1}}(v_{j,t}^{\text{snow}}{)}^{0.88}\right]}^{\text{2}}}\frac{\pi \rho }{\tau \rho } \end{array}$$ (20)

$$\begin{array}{c} v_{j,t}^{\text{snow}}=v_{t}^{\text{snow, max}}exp\left(-\frac{(x_{j}^{\text{line}}-x_{t}^{\text{snow, max}}{)}^{\text{2}}}{k_{\text{2}}}+\frac{(y_{j}^{\text{line}}-y_{t}^{\text{snow, max}}{)}^{\text{2}}}{k_{\text{2}}}\right) \end{array}$$ (21)

In the formula: $x_{j,t}^{\left(3\right),\text{line}}$, $v_{j,t}^{\text{ice}}$, $v_{j,t}^{\text{snow}}$ are the ice thickness, ice accretion rate, and snowfall rate on line *j* at time *t* respectively; $k_{\text{1}}$ is the conversion coefficient between maximum wind speed and average wind speed; ρ is the density of ice; $v_{t}^{\text{snow, max}}$ and $x_{t}^{\text{snow, max}}$, $y_{t}^{\text{snow, max}}$ are the maximum snowfall rate at time *t* and *t*he coordinates of the corresponding geographic location, respectively; $x_{j}^{\text{line}}$ and $y_{j}^{\text{line}}$ are the geographic coordinates of line *j*; $k_{\text{2}}$ is the attenuation coefficient.

Secondly, considering the combined effects of ice load and wind load, a line stress model is established, See Eqs. (22)–(24) for an example:


$$\begin{array}{c} F_{j,t}^{IW}=\sqrt{(F_{j,t}^{I}{)}^{\text{2}}+(F_{j,t}^{W}{)}^{\text{2}}} \end{array}$$ (22)

$$\begin{array}{c} F_{j,t}^{I}=9.8\times 10^{-3}\rho \pi (D_{j}+x_{j,t}^{\left(3\right),\text{line}})x_{j,t}^{\left(3\right),\text{line}} \end{array}$$ (23)

$$\begin{array}{c} F_{j,t}^{W}=CS_{j}v_{j,t}^{\text{wind}}(D_{j}+2x_{j,t}^{\left(3\right),\text{line}})\; \end{array}$$ (24)

In the formula: $F_{j,t}^{IW}$, $F_{j,t}^{I}$, $F_{j,t}^{W}$ are the ice-wind load, ice load, and wind load on line *j* at time *t* respectively; $D_{j}$ and $S_{j}$ are the conductor diameter and span factor of line *j*, respectively; $v_{j,t}^{\text{wind}}$ is the wind speed on line *j* at time *t*; *C* is a constan*t*.

Finally, according to the theory of metal deformation, the line failure probability exhibits an exponential increasing trend with increasing stress. When the stress exceeds the mechanical tolerance limit, direct conductor breakage occurs, See Eq. (25) for an example:


$$\begin{array}{c} p_{j,t}^{\text{dis, line}}=\left\{ \begin{array}{cc} @ll@0, & F_{j,t}^{\text{IW}}\leq F_{j}^{\text{IW,min}}\\ exp\left(\frac{0.69(F_{j,t}^{\text{IW}}-F_{j}^{\text{IW,min}})}{F_{j}^{\text{IW, max}}-F_{j}^{\text{IW,min}}}\right), & F_{j}^{\text{IW,min}}<F_{j,t}^{\text{IW}}<F_{j}^{\text{IW, max}}\\ 1, & F_{j,t}^{\text{IW}}\geq F_{j}^{\text{IW, max}} \end{array}\right. \end{array}$$ (25)

$$\begin{array}{c} F_{j}^{\text{IW,min}}=\frac{F_{j}^{\text{W, 1}}F_{j}^{\text{I, 1}}}{\sqrt{(F_{j}^{\text{W, 1}}cos\theta_{t}{)}^{\text{2}}+(F_{j}^{\text{I, 1}}sin\theta_{t}{)}^{\text{2}}}} \end{array}$$ (26)

$$\begin{array}{c} F_{j}^{\text{IW, max}}=\frac{F_{j}^{\text{W, 2}}F_{j}^{\text{I, 5}}}{\sqrt{(F_{j}^{\text{W, 2}}cos\theta_{t}{)}^{\text{2}}+(F_{j}^{\text{I, 5}}sin\theta_{t}{)}^{\text{2}}}} \end{array}$$ (27)

In the formula: $F_{j}^{\text{IW,min}}$ and $F_{j}^{\text{IW, max}}$ are the lower and upper limits of the ice-wind load on line *j* respectively; $F_{j}^{\text{I, 1}}$ and $F_{j}^{\text{W, 2}}$ are the wind forces on the line when the wind speed is 1 times and 2 times the design wind speed of line *j* respectively; $F_{j}^{\text{I, 1}}$ and $F_{j}^{\text{I, 5}}$ are the load forces on the line due to ice accretion when the ice thickness is 1 times and 5 times the design ice thickness of line *j*, respectively; $\theta_{t}$ is the angle between the resultant force of the ice load and wind load on the line at time *t* and the vertical direction, which can be expressed as $\theta_{t}=arctan\left(F_{j}^{\text{W},\text{\textbackslash{}hspace\{0.33em}t}\}/F_{j}^{\text{I},\text{\textbackslash{}hspace\{0.33em}t}\}\right)$.

(3) Operating-state-dependent failure probability

After extreme weather causes grid faults, power flow is redistributed, which can easily lead to limit violations in line power flow, transformer loading rates, and generator voltages, thereby increasing the operating failure probability of equipment. Linear models are used to fit the operating failure probabilities of three types of equipment [36], See Eqs. (28)–(30) for an example:


$$\begin{array}{c} p_{j,t}^{op,\text{line}}=\left\{ \begin{array}{cc} @ll@0 & \;I_{j,t}^{\text{line}}<I_{j}^{\text{line, rate}}\\ \frac{I_{j}^{\text{line}}-I_{j}^{\text{line, rate}}}{I_{j}^{\text{line, set}}-I_{j}^{\text{line, rate}}} & \;I_{j}^{\text{line, rate}}\leq I_{j,t}^{\text{line}}\leq I_{j}^{\text{line, set}}\\ 1 & I_{j,t}^{\text{line}}>I_{j}^{\text{line, set}} \end{array}\right. \end{array}$$ (28)

$$\begin{array}{c} p_{j,t}^{\text{op, trf}}=\left\{ \begin{array}{cc} @ll@0 & I_{j,t}^{\text{trf}}<I_{j}^{\text{trf, rate}}\\ \frac{I_{j,t}^{\text{trf}}-I_{j}^{\text{trf, rate}}}{I_{j}^{\text{trf, set}}-I_{j}^{\text{trf, rate}}} & I_{j}^{\text{trf, rate}}\leq I_{j,t}^{\text{trf}}\leq I_{j}^{\text{trf, set}}\\ 1 & I_{j,t}^{\text{trf}}\geq I_{j}^{\text{trf, set}} \end{array}\right. \end{array}$$ (29)

$$\begin{array}{c} P_{j,t}^{\text{op, gen}}=\left\{ \begin{array}{cc} @ll@1 & U_{j,t}^{\text{gen}}<U_{j}^{\text{gen, set1}}\cup U_{j,t}^{\text{gen}}>U_{j}^{\text{gen, set2}}\\ \frac{U_{j}^{\text{gen,min}}-U_{j,t}^{\text{gen}}}{U_{j}^{\text{gen,min}}-U_{j}^{\text{gen, set1}}} & U_{j}^{\text{gen, set1}}⩽U_{j,t}^{\text{gen}}<U_{j}^{\text{gen,min}}\\ 0 & U_{j}^{\text{gen,min}}⩽U_{j,t}^{\text{gen}}⩽U_{j}^{\text{gen, max}}\\ \frac{U_{j,t}^{\text{gen}}-U_{j}^{\text{gen, max}}}{U_{j}^{\text{gen, set2}}-U_{j}^{\text{gen, max}}} & U_{j}^{\text{gen, max}}<U_{j,t}^{\text{gen}}⩽U_{j}^{\text{gen, set2}} \end{array}\right. \end{array}$$ (30)

Where: $P_{j,t}^{\text{op, gen}}$, $p_{j,t}^{op,\text{line}}$ and $p_{j,t}^{\text{op, trf}}$ are the operational failure probabilities of line *j* transformer *j*, and generator *j* at time *t*, respectively; $I_{j}^{\text{line}}$ and $I_{j,t}^{\text{trf}}$ are the load currents of line *j* and transformer *j* at time *t*, respectively; $I_{j}^{\text{line, rate}}$ and $I_{j}^{\text{trf, rate}}$ are the rated load currents of line *j* and *t*ransformer *j* respectively; $I_{j}^{\text{line, set}}$ and $I_{j}^{\text{trf, set}}$ are the set values for the overcurrent protection of line *j* and transformer *j*, respectively; $U_{j,t}^{\text{gen}}$ is the terminal voltage of generator *j* at *t*ime *t*; $U_{j}^{\text{gen, max}}$ and $U_{j}^{\text{gen,min}}$ are the upper and lower limits of the normal terminal voltage for generator *j* respectively; $U_{j}^{\text{gen, set1}}$ and $U_{j}^{\text{gen, set2}}$ are the set values for the undervoltage protection and overvoltage protection of generator *j*, respectively.

(4) Comprehensive time-varying failure probability

Considering that the above three types of factors affecting equipment failure are independent of each other, a series model is used to describe the comprehensive time-varying failure probability $P_{j,t}^{\text{unit}}$ of the three types of equipment, See Eq. (31) for an example:.


$$\begin{array}{c} P_{j,t}^{\text{unit}}=\left\{ \begin{array}{c} @l@1-(1-p_{j,t}^{\text{base}})(1-p_{j,t}^{\text{op, line}})(1-p_{j,t}^{\text{dis, line}})\\ 1-(1-p_{j,t}^{\text{base}})(1-p_{j,t}^{\text{op, trf}})(1-p_{j,t}^{\text{dis, trf}})\\ 1-(1-p_{j,t}^{\text{base}})(1-p_{j,t}^{\text{op, gen}}) \end{array}\right. \end{array}$$ (31)

Based on the comprehensive failure probability $P_{j,t}^{\text{unit}}$, the equipment status is sampled using the Monte Carlo method, See Eq. (32) for an example:. Assuming that the number of sampling iterations is M, the occurrence probability of the grid fault state is given by Eq. (33).


$$\begin{array}{c} y_{j,t}^{\left(3\right)},y_{j,t}^{\left(4\right)},y_{j,t}^{\left(5\right)}=\left\{ \begin{array}{cc} @ll@1 & p_{j,t}^{\text{unit}}>\varepsilon^{\prime }\\ 0 & p_{j,t}^{\text{unit}}⩽\varepsilon^{\prime } \end{array}\right. \end{array}$$ (32)

$$\begin{array}{c} P_{s,y_{i}^{\left(e\right)}|X}=\frac{\text{1}}{M}\text{\textbackslash{}hspace\{0.33em}\;\;\;\}e=3,4,5 \end{array}$$ (33)

Where: $y_{j,t}^{\left(3\right)},y_{j,t}^{\left(4\right)},y_{j,t}^{\left(5\right)}$ are the failure states of line j, transformer j, and thermal power unit j at time t, respectively. If a failure occurs, $y_{j,t}^{\left(e\right)}=1$; otherwise, $y_{j,t}^{\left(e\right)}=0$, for e=3,4,5. $\varepsilon^{\prime }$ is a random number that follows a uniform distribution in the interval [0,1].

### 3.3. System risk scenario generation based on autoencoder

The core concept of the variational autoencoder (VAE) is variational inference. If there exists a special mapping relationship between the real sample *x* and the latent variable z(z∈R), then, to improve the generalization ability of the model and introduce randomness into the generated scenarios, the latent variable is assumed to follow a unit normal distribution, which prevents the variance of the distribution followed by the latent variable from being zero; otherwise, the VAE model degenerates into an autoencoder (AE) model. When using the VAE to generate scenarios, the generative network is defined as $p_{\pi }(x\;|\;z)$, and the recognition network is defined as $q_{\phi }(z\;|\;x)$ [22]. where π and ϕ are network parameters. Since directly optimizing the maximum likelihood estimate is difficult, variational inference is used to optimize the evidence lower bound of the maximum likelihood function $logp_{\phi }\left(x\right)$, See Eq. (34) for an example:


$$\begin{array}{c} logp_{\phi }(x)=E_{q_{\phi }(z\;|\;x)}\left(log\left(p_{\pi }\left(x\;|\;z\right)\right)\right)-D_{KL}\left(q_{\phi }\left(z\;|\;x\right)∥p_{\pi }\left(z\right)\right) \end{array}$$ (34)

Where, $D_{KL}(•)$ denotes the function for calculating the Kullback Leibler (KL) divergence; $E_{q_{\phi }(z\;|\;x)}(•)$ represents the expectation function under the recognition network $q_{\phi }\left(z\text{|}x\right)$; $p_{\pi }\left(z\right)$ is the distribution of z.

Although VAE has achieved good performance in simple scenario generation, its ability to extract intrinsic statistical laws from historical data with complex coupling relationships and obtain posterior distributions of samples still needs improvement. To address the shortcomings of VAE, DVAE introduces noise into the latent variables and proposes a new denoising variational lower bound. DVAE adopts a variational inference denoising criterion based on a recognition network, which enables neighboring data points in a low dimensional manifold to be robust to noise in the high dimensional observation space, thereby constructing a scenario generation model with good generalization performance. Let the stochastic recognition network be $q_{\phi }\left(z\right|\tilde{x})\;N(z\left|\mu_{\phi \left(\tilde{x}\right)},\sigma_{\phi \left(\tilde{x}\right)}\right)$, where $\tilde{x}$ is the noisy data, $N(z\;|\;\mu_{\phi \left(\tilde{x}\right)},\sigma_{\phi \left(\tilde{x}\right)})$ is the normal distribution function, and $\mu_{\phi \left(\tilde{x}\right)}$ and $\sigma_{\phi \left(\tilde{x}\right)}$ are the mean and variance of the normal distribution, respectively. Then, we have:


$$\begin{array}{c} E_{p(\tilde{x}\;|\;x)}\left(q_{\phi }\left(z\;|\;\tilde{x}\right)\right)=\int_{\tilde{x}}q_{\phi }\left(z\;|\;\tilde{x}\right)p\left(\tilde{x}\;|\;x\right)d\tilde{x}\; \end{array}$$ (35)

Where, $p(\tilde{x}\;|\;x)$ denotes the noise distribution conditional on x; $E_{p(\tilde{x}\;|\;x)}(•)$ denotes the expectation function taken with respect to the distribution $p(\tilde{x}\;|\;x)$.

Each time, $\tilde{x}\sim \;p(\tilde{x}\;|\;x)$ is sampled and substituted into $q_{\phi }(z\;|\;\tilde{x})$, yielding different Gaussian distributions. Since $E_{p(\tilde{x}\;|\;x)}\left(q_{\phi }\left(z\;|\;\tilde{x}\right)\right)$ requires the introduction of a noise distribution, the estimation of the lower bound becomes difficult. Before introducing the denoising variational lower bound, a stochastic layer should first be added to $q_{\phi }(z\;|\;x)$ and the integral over the random variable should be computed, that is:


$$\begin{array}{c} q_{\phi }(z\;|\;x)=\int_{z}q_{\phi }\left(z\;|\;z^{\prime }\right)q_{\psi }\left(z^{\prime }\;|\;x\right)dz^{\prime }\; \end{array}$$ (36)

Where, $z^{\prime }\;$ denotes the introduced random variable; ϕ={φ,ψ},where, φ and ψ are the network parameters of the stochastic layers.

Let the joint generative network of *x* and *z* be $p_{\pi }\left(x,z\right)=p_{\pi }\left(x\;|\;z\right)p_{\pi }(z)$; and let the stochastic layer recognition network be ${\tilde{q}}_{\phi }(z\;|\;x)=\int q_{\phi }\left(z\;|\;\tilde{x}\right)p\left(\tilde{x}\;|\;x\right)d\tilde{x}$. Then, we obtain:


$$\begin{array}{c} logp_{\pi }(x)\geq E_{{\tilde{q}}_{\phi }(z\;|\;x)}\left(log\frac{p_{\pi }\left(x,z\right)}{q_{\phi }\left(z\;|\;x\right)}\right) \end{array}$$ (37)

Where, $E_{{\tilde{q}}_{\phi }(z\;|\;x)}$ denotes the expectation function under the distribution ${\tilde{q}}_{\phi }(z\;|\;x)$.

The denoising variational lower bound $L_{dvae}$ is given as follows:


$$\begin{array}{c} L_{\text{dvae}}=E_{{\tilde{q}}_{\phi }(z\;|\;x)}\left(log\frac{p_{\pi }\left(x,z\right)}{q_{\phi }\left(z\;|\;x\right)}\right) \end{array}$$ (38)

The maximized lower bound is:


$$\begin{array}{c} logp_{\pi }(x)\geq L_{\text{dvae}}+E_{p\left(\tilde{x}\;|\;x\right)}\left(D_{\text{KL}}\left({\tilde{q}}_{\phi }\left(z\;|\;x\right)∥p\left(z\;|\;x\right)\right)\right) \end{array}$$ (39)

Where, $p_{\pi (z\;|\;x)}$ denotes the posterior distribution.

The DVAE has a structure similar to that of the VAE, but the encoder does not directly output a latent variable; instead, it outputs a multidimensional normal distribution, from which the latent variable z is then sampled. The sampling process of the multidimensional normal distribution is approximated using Monte Carlo sampling as follows:


$$\begin{array}{c} L_{\text{dvae}}=E_{p(\tilde{x}\;|\;x)}\left(E_{q(z\;|\;\tilde{x})}\left(log\frac{p_{\pi }(x,z)}{q_{\phi }(z\;|\;\tilde{x})}\right)\right)\approx \frac{\text{1}}{\Phi \Psi }\sum_{\beta =1}^{\Phi }\sum_{\alpha =1}^{\Psi }log\frac{p_{\pi }\left(x,z^{\left(\alpha \;|\;\beta \right)}\right)}{q_{\phi }\left(z^{\left(\alpha \;|\;\beta \right)}\;|\;{\tilde{x}}^{\left(\beta \right)}\right)} \end{array}$$ (40)

Where, ${\tilde{x}}^{\left(\beta \right)}\;p(\tilde{x}\;|\;x)$ denotes the introduced noise variable samples; $z^{\left(\alpha \;|\;\beta \right)}\;q_{\phi }(z\;|\;{\tilde{x}}^{\left(\beta \right)})$ denotes the introduced latent variable samples; $q(z\;|\;\tilde{x})$ is the stochastic posterior distribution; Φ is the total number of noise variable samples; Ψ is the total number of latent variable samples.

The training process of the DVAE and the calculation of its loss function are similar to those of the VAE.


$$\begin{array}{c} logp_{\pi }(x)=E_{{\tilde{q}}_{\phi }(z\;|\;x)}\left(log\frac{p_{\pi }\left(x,z\right)}{{\tilde{q}}_{\phi }(z\;|\;x)}\right)≜L_{\text{cvae}} \end{array}$$ (41)

Where, $L_{cvae}$ denotes the lower bound to be maximized.

Compared with other scenario generation algorithms, DVAE can satisfy the requirements for generating complex scenarios. By introducing the denoising variational lower bound, it improves the flexibility of the constructed variational distribution and is more suitable for generating multidimensional scenario sets with complex coupling characteristics, such as those for wind, solar, and temperature.

## 4. Construction of risk assessment index system and calculation model for supply demand imbalance

Based on the generated risk scenarios, this chapter constructs a system operation risk assessment index system from the three dimensions of adequacy, flexibility, and security and stability, and establishes a time series power supply–demand balance optimization model under extreme weather conditions, thereby providing physical constraints and evaluation targets for subsequent deep learning methods.

### 4.1. Supply-demand imbalance risk assessment indicators

#### 4.1.1. System operational adequacy index.

Adequacy refers to the power generation, transmission, and supply capacity [37] of the system after considering factors such as partial equipment outages or insufficient output. As the proportion of renewable energy in the planned grid gradually increases, and load peaks in summer and winter, events such as high temperatures with no wind or blizzards may lead to insufficient renewable energy output, sharp load growth, and high equipment failure rates, exposing the system to the risk of inadequacy. Therefore, two consequence indicators, namely loss of power and loss of energy, are used to evaluate the adequacy of the planning scheme, See Eqs. (42)–(43) for an example:


$$\begin{array}{c} C_{s,1,t}=\sum_{i\in \Omega^{N}}\frac{P_{i,t}^{\text{L, cut}}}{P_{i,t}^{N}},\text{\textbackslash{}hspace\{0.33em}\;\;\;\}\forall t\in \Omega^{T} \end{array}$$ (42)

$$\begin{array}{c} C_{s,2,t}=\sum_{t\in \Omega^{T}}\sum_{i\in \Omega^{N}}P_{i,t}^{\text{L, cut}} \end{array}$$ (43)

Where, $C_{s,1,t}$ denotes the unsupplied power of the system at time t under risk scenario s; $\Omega^{N}$ denotes the set of nodes; $P_{i,t}^{\text{L, cut}}$ denotes the load curtailment at node i at time t; $P_{i,t}^{N}$ denotes the total load demand at node i at time t; $C_{s,2,t}$ denotes the unsupplied energy of the system at time t under risk scenario s.

#### 4.1.2. System operational flexibility index.

Flexibility refers to the ability of the system to respond quickly to disturbance events [37] In traditional power systems, controllable regulation resources are relatively abundant, and the risk of insufficient flexibility is not severe. However, in planned grids, conventional units are gradually being replaced by renewable energy units, leading to a decline in regulation capability. When extreme events such as high temperatures with no wind or blizzards increase the system’s flexibility demand, even the full output of controllable power sources may fail to meet the load demand, exposing the system to the risk of insufficient flexibility. Therefore, the flexibility indicator is incorporated into the evaluation framework, and the insufficient spinning reserve consequence indicator is used to assess the flexibility of the planning scheme, See Eq. (44) for an example:


$$\begin{array}{c} C_{s,3,t}=\left\{ \begin{array}{cc} @ll@\sum_{i\in \Omega^{N}}\frac{P_{i,t}^{N}-(\theta_{i,t}^{G}+P_{i,t}^{G})}{P_{i,t}^{N}} & \sum_{i\in \Omega^{N}}\theta_{i,t}^{G}+P_{i,t}^{G}<P_{i,t}^{N}\\ 0 & \sum_{i\in \Omega^{N}}\theta_{i,t}^{G}+P_{i,t}^{G}\geq P_{i,t}^{N} \end{array}\right. \end{array}$$ (44)

Where, $\forall t\in \Omega^{T}$; ${\text{C}}_{\text{s,3,t}}$ denotes the spinning reserve deficit of the system at time t under risk scenario s; $\theta_{i,t}^{G}$ denotes the spinning reserve of conventional generating units at node i at time t; $P_{i,t}^{G}$ denotes the total output of all types of generating units at node i at time t.

#### 4.1.3. System operation safety and stability index.

Security and stability refer to the ability of the system to avoid loss of control and large scale blackouts under contingency conditions [37]. The planned grid exhibits significant “dual-high” characteristics (high penetration of renewable energy and high proportion of power electronics), which not only leads to a sharp increase in the number and scale of equipment failures under blizzard conditions, but also reduces the system’s disturbance rejection capability. After a system fault, frequency and voltage fluctuations may occur, further triggering more severe events such as grid disconnection and cascading failures [37], exposing the system to the risk of insufficient security and stability [38]. Therefore, indicators including voltage violation, power flow violation, transformer loading rate violation, and frequency violation are used to evaluate the security and stability of the planning scheme, See Eqs. (45)–(48) for an example:


$$\begin{array}{c} C_{s,4,t}=\left\{ \begin{array}{cc} @ll@0 & V^{min}\leq {\hat{V}}_{i,t}\leq V^{max}\\ \sum_{i\in \Omega^{N}}|{\hat{V}}_{i,t}-V^{\text{lim}}| & otherwise \end{array}\right. \end{array}$$ (45)

$$\begin{array}{c} C_{s,5,t}=\left\{ \begin{array}{cc} @ll@\sum_{\left(i,j\right)\in \Omega^{B}}({\hat{P}}_{\left(i,j\right),t}-P^{\text{max}}) & {\hat{P}}_{\left(i,j\right),t}>P^{\text{max}}\\ 0 & otherwise \end{array}\right. \end{array}$$ (46)

$$\begin{array}{c} C_{s,6,t}=\left\{ \begin{array}{cc} @ll@\sum_{i\in \Omega^{N}}({\hat{Z}}_{i,t}-Z^{\text{max}}) & {\hat{Z}}_{i,t}>Z^{\text{max}}\\ 0 & otherwise \end{array}\right. \end{array}$$ (47)

$$\begin{array}{c} C_{s,7,t}=\left\{ \begin{array}{cc} @ll@{\hat{f}}_{t}-f^{\text{max}} & {\hat{f}}_{t}>f^{\text{max}}\\ 0 & otherwise \end{array}\right. \end{array}$$ (48)

Where, $\forall t\in \Omega^{T}$; $C_{s,4,t}$, $C_{s,5,t}$, $C_{s,6,t}$, $C_{s,7,t}$ denote the voltage violation amount, power flow violation amount, transformer load rate violation amount, and system frequency decline rate violation amount at time t under risk scenario s, respectively; ${\hat{V}}_{i,t}$ denotes the perunit voltage at node i at time t; $V^{\text{max}}$ and $V^{\text{min}}$ denote the upper and lower limits of node voltage, respectively; $V^{\text{lim}}$ denotes the voltage limit, where $V^{\text{lim}}$ takes the value of $V^{max}$ when ${\hat{V}}_{i,t}>V^{max}$,and takes the value of $V^{min}$ when ${\hat{V}}_{i,t}<V_{min}$; $\Omega^{B}$ denotes the set of branches; ${\hat{P}}_{\left(i,j\right),t}$ denotes the perunit power flow on line (i,j) at time t; $P^{\text{max}}$ denotes the upper limit of line power flow; ${\hat{Z}}_{i,t}$ denotes the load rate of the transformer at node i at time t; $Z^{\text{max}}$ denotes the upper limit of the transformer load rate; ${\hat{f}}_{t}$ denotes the perunit value of the maximum system frequency decline rate at time t; $f^{\text{max}}$ denotes the limit of the system frequency decline rate.

### 4.2. Supply-demand imbalance risk assessment model

Eqs. (49)–(56) construct a long term, time series power supply-demand balance model under persistent stagnant weather conditions. Specifically, Eq. (50) calculates the injected power at each network node; Eq. (51) represents the corresponding nodal power balance constraint; Eq. (52) calculates the power flow distribution of the entire network; Eq. (53) defines the upper and lower output limits of conventional thermal power units; Eq. (54) specifies the upper and lower output limits of renewable energy under extreme stagnant weather conditions; Eq. (55) gives the upper and lower limits of power flow in the network; and Eq. (56) sets the upper limit of load shedding power.


$$\begin{array}{c} p_{\varepsilon_{\delta },t}^{\text{rd}}={\left[p_{\varepsilon_{\delta },t}^{\text{rd, coal}},p_{\varepsilon_{\delta },t}^{\text{rd, res}}\right]}^{T} \end{array}$$ (49)

$$\begin{array}{c} p_{\varepsilon_{\delta },t}^{\text{rd,in}}=\left|p_{\varepsilon_{\delta },t}^{\text{rd}}\right|-(d_{\varepsilon_{\delta },t}^{\text{rd}}-d_{\varepsilon_{\delta },t}^{\text{rd, cur}}) \end{array}$$ (50)

$$\begin{array}{c} {\text{1}}^{T}p_{\varepsilon_{\delta },t}^{\text{rd}}=0 \end{array}$$ (51)

$$\begin{array}{c} f_{\varepsilon_{\delta },t}^{\text{rd}}=\varpi p_{\varepsilon_{\delta },t}^{\text{rd,in}} \end{array}$$ (52)

$$\begin{array}{c} 0\leq p_{\varepsilon_{\delta },t}^{\text{rd, coal}}\leq u^{\text{coal}} \end{array}$$ (53)

$$\begin{array}{c} 0\leq p_{\varepsilon_{\delta },t}^{\text{rd, res}}\leq u^{\text{res}}\pi_{\varepsilon_{\delta },t}^{\text{rd}} \end{array}$$ (54)

$$\begin{array}{c} \underset{―}{f}\leq f_{\varepsilon_{\delta },t}\leq \overset{―}{f} \end{array}$$ (55)

$$\begin{array}{c} 0\leq {\underset{―}{d}}_{\varepsilon_{\delta },t}^{\text{rd, cur}}\leq {\overset{―}{d}}_{\varepsilon_{\delta },t}^{\text{rd}} \end{array}$$ (56)

Where, the superscript rd denotes the low output scenarios of renewable energy sources under extreme static stability weather conditions; $p_{\varepsilon_{\delta },t}^{\text{rd}}$ denotes the output power vector of generating units at time interval t in the $\varepsilon_{\delta }-th$ scenario; $p_{\varepsilon_{\delta },t}^{\text{rd, coal}}$ and $p_{\varepsilon_{\delta },t}^{\text{rd, res}}$ denote the output power of coal-fired units and renewable energy generating units at time interval t in the $\varepsilon_{\delta }-th$ scenario, respectively; $u^{\text{coal}}$ denotes the installed capacity of conventional coal-fired units; $u^{\text{res}}$ denotes the installed capacity of renewable energy units; $p_{\varepsilon_{\delta },t}^{\text{rd,in}}$ denotes the injection power of all nodes in the network at time interval t in the $\varepsilon_{\delta }-th$ scenario; $d_{\varepsilon_{\delta },t}^{\text{rd}}$ and $d_{\varepsilon_{\delta },t}^{\text{rd, cur}}$ denote the load demand energy and curtailed load energy at time interval t in the $\varepsilon_{\delta }-th$ scenario, respectively; ϖ denotes the generation shifted distribution factor (GSDF) of the system; $f_{\varepsilon_{\delta },t}^{\text{rd}}$ denotes the network power flow at time interval t in the $\varepsilon_{\delta }-th$ scenario; $\pi_{\varepsilon_{\delta },t}^{\text{rd}}$ denotes the renewable energy output fluctuation at time interval t in the $\varepsilon_{\delta }-th$ scenario; $\overset{―}{f}$ and f denote the upper and lower limits of the power flow in the power network, respectively.

### 4.3. Risk assessment process of supply-demand imbalance

The overall assessment process of this paper is shown in Fig 2. It consists of three parts: generating risk scenarios and calculating their occurrence probabilities, calculating the system state based on the planning platform, and calculating various system risk indicators. The specific steps are as follows:

**Fig 2:**
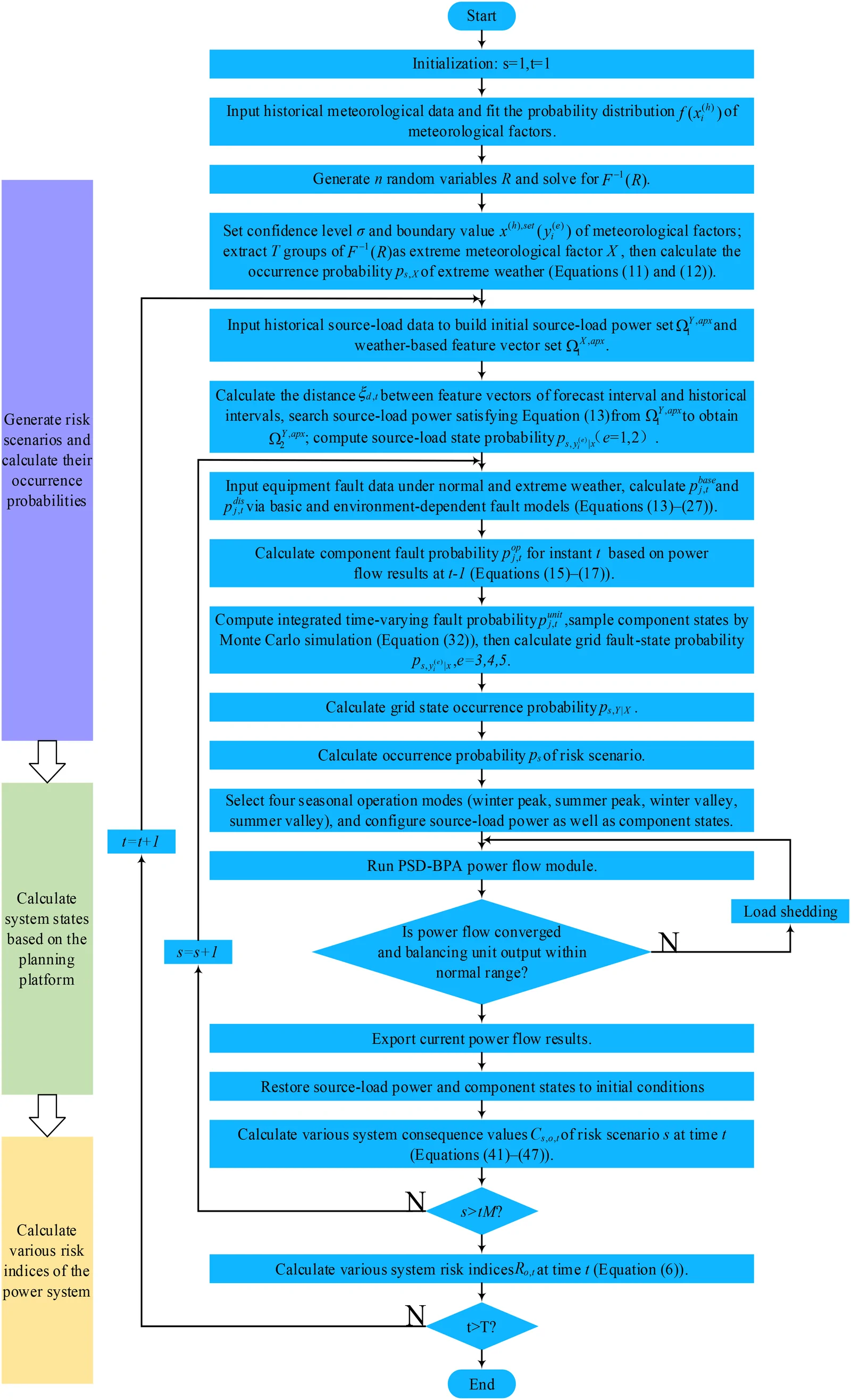
Flow chart of risk assessment for power grid based on planning platform.

1) Initialize the number of risk scenarios s and the assessment time step t.

2) Generate extreme weather conditions for risk scenarios: input historical weather data and fit the probability density function $f\;\left(x_{t}^{\text{(}h\text{)}}\right)$ of meteorological factors; generate n random variables R and compute $F^{-1}\left(R\right)$; under the confidence value σ set the boundary values of meteorological factors as $x^{\left(h\right),set}\left({y_{t}}^{\left(e\right)}\right)$, select *T* groups of values satisfying the extreme weather conditions from $F^{-1}\left(R\right)$ as the meteorological factors *X*, and calculate the occurrence probability of extreme weather ${\text{p}}_{\text{s},X}$.
3) Generate equipment states in risk scenarios: input historical equipment failure data under normal weather and the generated extreme weather data, calculate the basic failure probability ${\text{p}}_{j,t}^{base}$ and the environment dependent failure probability $p_{j,t}^{dis}$; based on the power flow results at time t−1, estimate the failure probability $p_{j,t}^{op}$ considering the system state at time *t*, calculate the comprehensive time varying failure probability $p_{j,t}^{unit}$, and use the Monte Carlo method to obtain equipment states $\left(y_{j\text{,}t}^{\left(3\right)},y_{\;j,t}^{\left(4\right)},y_{\;j,t}^{\left(5\right)}\right)$, and calculate the grid fault s*t*ate probability $p_{s,y_{t}^{\left(e\right)}|X}\;(e=3,4,5)$.
4) Calculate the occurrence probability of the grid state $p_{s,Y|X}$ according to Equation (10), and calculate the occurrence probability of the risk scenario $p_{s}$ ccording to Equation (36).
5) Set up the planning platform: under the selected system operating mode, the ratios are set as $y_{d,t}^{\left(1\right),apx}/\;y_{d,t}^{\left(1\right),orig}\text{,}y_{d,t}^{\left(2\right),apx}/\;y_{d,t}^{\left(2\right),orig}$ (which $\;y_{d,t}^{\left(1\right),orig}\text{,}y_{d,t}^{\left(2\right),orig}$ represent the initial renewable energy power value and initial load power value of the platform, respectively). The fault equipment states are sequentially set according to the sampling results of step 4).
6) Power flow calculation: use the power flow calculation module PSD‑BPA to compute the power flow, adjust the load ratio until convergence, export the calculation results, and restore the platform to its initial state.
7) Risk consequence calculation: substitute the power flow calculation results $\text{(}\Delta P_{i,t}^{L,pred},P_{i,t}^{G},{\hat{V}}_{i,t},{\hat{P}}_{\left(i,j\right)},t,{\hat{Z}}_{i,t},{\hat{f}}_{t}\text{)}$ into the consequence indicator formulas to complete the risk consequence calculation for the risk scenario at that time step.
8) Risk indicator calculation: traverse the consequence indicators of all scenarios at that time step to complete the calculation of various system risk indicators at that time step.

## 5. Deep learning solution method coupled with attention mechanism

### 5.1. Basis for MDP modeling and DDPG selection

This paper formulates the power balance risk assessment under extreme weather as a Markov Decision Process (MDP), whose rationality is elaborated as follows:

(1) The evolution of extreme weather bears prominent temporal cumulative effects. Equipment aging, ice accretion and cumulative load response all constitute continuous-time processes, and the risk state at the current moment depends on meteorological conditions and system operation trajectories of preceding time steps.
(2) Risk control is essentially a sequential decision-making task. Dispatchers need to dynamically adjust emergency plans according to risk evolution trends, rather than making one-off static decisions.
(3) The “offline training – online inference” paradigm of reinforcement learning perfectly satisfies the cross-scenario transfer requirement from historical meteorological-operation data to real-time rapid risk identification.

Although supervised learning can directly learn the mapping from system states to risk indicators, it fails to explicitly model the temporal dependency of risk evolution and reward feedback mechanisms. Therefore, this paper adopts Deep Deterministic Policy Gradient (DDPG) as the basic framework, and embeds an attention mechanism to strengthen its capability of extracting critical features within high-dimensional state spaces.

The Markov Decision Process is defined as:


$$\begin{array}{c} M=\text{(}S\text{,}A\text{, P,}r\text{,}\gamma \text{)} \end{array}$$ (57)

Where ***S*** denotes the state space, ***A*** represents the continuous action space, P stands for the state transition law over time-series scenarios, and *r* is the reward function, with γ being the discount factor. The state at time step *t* is defined as:


$$\begin{array}{c} S_{t}=[x_{t}^{\text{T}},y_{t-1}^{\text{T}},\Delta x_{t}^{\text{T}}{]}^{\text{T}} \end{array}$$ (58)

where $x_{t}$ consists of normalized meteorological variables, renewable energy output, load variables, equipment failure probabilities or states, and power grid operating features; $y_{t-1}$ denotes the estimated vector of risk indicators at the previous time step; $\Delta x_{t}$ represents the first-order temporal difference.

The action is defined as a K-dimensional continuous normalized risk estimation vector:


$$\begin{array}{c} A_{t}={\hat{y}}_{t}=[{\hat{R}}_{1,t},...,{\hat{R}}_{k,t}{]}^{\text{T}}\in [0,1{]}^{K} \end{array}$$ (59)

Each component of the action corresponds to the adequacy, flexibility, security and stability indicators defined in Section 4. After an action is generated, the environment proceeds to the next hour of the same scenario, and each episode terminates after T = 72 steps. DDPG-Attention, vanilla DDPG, SAC, PPO and DQN adopt identical definitions of states, actions, state transitions and reward functions. For DQN, each continuous indicator is discretized via the same binning scheme used in implementation.

The reward function is composed of the deviation between the predicted risk vector and the PSD‑BPA labels, as well as penalties for temporal consistency and physical constraints.


$$\begin{array}{c} r_{t}=-\sum_{K=1}^{K}\lambda_{k}({\hat{\text{R}}}_{K,t}-R_{K,t}^{\text{BPA}}{)}^{\text{2}}-\lambda_{s}{\|A_{t}-A_{t-1}\|}_{\text{2}}^{\text{2}}-\lambda_{c}\Omega (A_{t}) \end{array}$$ (60)

In Eq. (60), $R_{K,t}^{\text{BPA}}$ denotes the *K*-th risk label calculated by PSD-BPA; $\lambda_{k}$ is the weight coefficient to balance indicators with different dimensions and scales; $\lambda_{s}$ serves as the penalty factor for unreasonable hourly abrupt fluctuations; $\lambda_{c}$ denotes the physical constraint penalty function, which imposes penalties on outputs that exceed the normalized range or violate preset indicator constraints; Ω(•) is the penalty operator that penalizes outputs exceeding the normalized interval and violating preset indicator constraints. All coefficients must be fixed prior to testing and reported in Table 2.

**Table 2:** Values for each type of operating parameter.

ParameterValueApplicable Models	ParameterValueApplicable Models	ParameterValueApplicable Models
Training episodes1000All reinforcement learning baselines	Training episodes1000All reinforcement learning baselines	Training episodes1000All reinforcement learning baselines
Episode length/ time step	72 h/ 1 h	All reinforcement learning baselines
Discount factor γ	0.99	All reinforcement learning baselines
Reward weights λ_k_、λ_s_、λ_c_	λ_k_ = 1.0 for each normalized risk indicator; λ_s_ = 0.05 for temporal consistency; λc = 0.10 for physical constraints	All reinforcement learning baselines
Actor/Critic learning rate	DDPG: 1 × 10^−4^; DDPG-Attention: 1 × 10^−3^; SAC: 3 × 10^−4^/ 3 × 10^−4^	DDPG、DDPG-Attention、SAC
Batch size/ Replay buffer capacity	256/ 1 × 10⁶ transition samples	DDPG、DDPG-Attention、SAC、DQN
Soft update coefficient τ for target network	0.005	DDPG、DDPG-Attention、SAC
Exploration noise process and scale	Ornstein–Uhlenbeck noise: θ = 0.15; Σ linearly decays from 0.20 to 0.05	DDPG、DDPG-Attention
MLP hidden layers, activation function and optimizer	Layer size: 256–128–64; ReLU activation; Adam optimizer with learning rate 1 × 10^−3^; L2 regularization 1 × 10^−5^; early stopping patience = 30	Supervised MLP
Discretization and training hyperparameters of DQN	11 equally spaced bins for each risk indictor (0–1 with step 0.1); learning rate1 × 10^−4^; ε decays from1.0 to 0.05	DQN
Training hyperparameters of PPO	Learning rate 3 × 10^−4^; coefficient = 0.20; GAE λ = 0.95; update for 10 iterations per mini-batch; entropy coefficient = 0.01	PPO
Entropy parameter of SAC	Automatically adjustable temperature coefficient; target entropy =−K; temperature learning rate 3 × 10^−4^	SAC
Attention module configuration	5 types of feature tokens (meteorology, power sources, power grid, load, historical risk); token dimension = 64; single-head scaled dot-product attention; Dropout rate = 0.10	DDPG-Attention
Dataset scenario partition	1120 training samples, 240 validation samples, 240 independent test samples (70%/ 15%/ 15%)	All models
Composition of weather conditions	Training set: 560 winter snowstorm scenarios, 560 high-temperature no-wind scenarios; Validation set: 120/ 120; Test set: 120/ 120. Stratified balanced distribution of peak/valley operation modes and power fluctuation ratios σ=−20%、 − 10%、0%、10%、20% in each subset	All models
Random seed for scenario-level partitioning	2026 (fixed for dataset split)	All models
Random seeds for training and evaluation	2021–2030 (10 independent runs)	All models
Normalization method	Min–Max normalization mapped to [0,1] using statistical metrics of the training set; the same normalization parameters are applied to validation and test sets	All models

Let $\pi_{\theta }$ denote the Actor network parameterized by θ. The policy optimization objective is defined as the expected discounted cumulative reward over the state distribution:


$$\begin{array}{c} J\left(\theta \right)={\text{E}}_{\pi \theta }\left[\sum_{t=0}^{\text{T}-1}\gamma^{t}r_{t}\right] \end{array}$$ (61)

Where T = 72 represents the total time steps of the evaluation period, and $r_{t}\in \left(0,1\right]$ is the discount factor. $r_{t}$ is defined in Equation (60). By maximizing J(θ), the Actor network is trained to output risk assessment values with minimal deviation from PSD-BPA simulation results while satisfying physical constraints, and meanwhile guarantee the temporal smoothness of outputs between adjacent time instants.

DDPG is selected because the actions consist of continuous multi-indicator vectors, and the 72-hour scenarios constitute a sequential estimation problem. This paper does not presuppose that DDPG outperforms supervised learning by default; instead, its effectiveness is evaluated through empirical comparisons with the MLP baseline on an independent test set.

### 5.2. Actor network with introduced attention mechanism

If the attention score is merely a scalar, the Softmax operation will always yield trivial weights of 1. Therefore, this paper divides the hidden representations into m feature tokens, such as feature groups corresponding to meteorology, power sources, power grids, loads, and risks at the previous time step. The token matrix is defined as:


$$\begin{array}{c} H_{t}\;=reshape\left(\phi \left(W_{s}\;S_{t}+b_{s}\right)\;,\text{m},\text{d}\right) \end{array}$$ (62)

Where $H_{t}\;\in {\mathbb{R}}^{\text{m}\times \text{d}}$, $W_{s}$ represents the weight matrix for linear transformation from states to hidden layers, $b_{s}$ denotes the bias vector, m is the number of feature tokens, and d is the token dimension.

The query, key and value matrices are defined as:


$$\begin{array}{c} \left\{ \begin{array}{c} @l@Q=H_{t}W_{Q}\\ K=H_{t}W_{K}\\ V=H_{t}W_{V} \end{array}\right. \end{array}$$ (63)

The attention weight matrix is computed row-wise:


$$\begin{array}{c} A_{t}\;=\text{softmax(}\frac{QK^{\text{T}}}{\sqrt{d_{k}}}\text{),}\sum_{j=1}^{\text{m}}A_{t,ij}=1 \end{array}$$ (64)

Accordingly, $A_{t}\;\in {\mathbb{R}}^{\text{m}\times \text{m}}$ is an attention weight matrix rather than a scalar, and the weights in each row sum to 1.The residual attention representation is expressed as:


$$\begin{array}{c} H_{t}^{att}=A_{t}V+H_{t} \end{array}$$ (65)

After vectorization, the Actor network outputs the risk vector:


$$\begin{array}{c} a_{t}=\pi_{\theta }\left(S_{t}\right)=g_{out}\left(W_{o}vec\left(H_{t}^{att}\right)+b_{o}\right) \end{array}$$ (66)

Where $\pi_{\theta }$ denotes the policy network with parameter θ; $S_{t}$ is the state vector at time step *t*; $a_{t}\in {\mathbb{R}}^{d_{a}}$ represents the action vector at time step *t*, namely the comprehensive risk assessment value, where $d_{a}$ dnotes *t*he number of risk indicator categories; vec(•) refers to the matrix vectorization operator that fla*tt*ens $H_{t}^{att}$ into a one-dimensional vector; $W_{o\;}\in {\mathbb{R}}^{d_{a}\times (m\cdot d)}$ and $b_{o\;}\in {\mathbb{R}}^{d_{a}}$ are the weight matrix and bias vector of the output layer, respectively; $g_{out}(•)$ is the activation function of the output layer. In this paper, the Sigmoid function is adopted to map outputs to the interval [0,1].


$$\begin{array}{c} \nabla_{\theta }\;J\left(\theta \right)\approx E\left[\nabla_{a}\;Q_{\psi }\;\left(s,a)\;|a=\pi \theta \;\left(s\right)\;\cdot \nabla_{\theta }\;\pi_{\theta }\;(s\right)\right] \end{array}$$ (67)

Where $\nabla_{\theta }\;J\left(\theta \right)$ represents the gradient of the objective function with respect to policy network parameter θ; $Q_{\psi }\;\left(s,a\right)$ denotes the Critic network. ψ denotes the network parameter for evaluating the value of state-acion pairs; $\nabla_{a}\;Q_{\psi }\;\left(s,a\right)$ is the partial derivative of the Critic network output with respect to action a, which charcterizes the sensitivity of the value function to action adjustments; $\nabla_{\theta }\;\pi_{\theta }\;\left(s\right)$ refers to the partial derivative of the policy network output with respect to its paramter θ.The Actor network maximizes the objective function by updating parameters along the gradient direction, formulated as $\theta \leftarrow \theta +\alpha \nabla_{\theta }\;J\left(\theta \right)$, where α is the learning rate of the Actor network.

Experience replay and target networks are employed to realize stable off-policy training. To guarantee a fair comparison, all reinforcement learning baseline algorithms adopt the same scenario partitioning scheme, reward coefficients, training episodes and termination criteria. The irreproducible implementation parameters in the current manuscript have been marked in Table 2, which should be supplemented based on the original source codes or rerunning logs.

### 5.3. Supervised learning baselines and evaluation metrics on independent test set

The non-reinforcement learning baseline adopts feedforward MLP and is directly trained with identical PSD-BPA labels:


$$\begin{array}{c} {\hat{y}}_{t}=f_{MLP}(s_{t};\sigma ) \end{array}$$ (68)

Where ${\hat{y}}_{t}\in {\mathbb{R}}^{K}$ denotes the predicted risk vector at time step *t*; *K* represents the number of risk indicator ca*t*egories (*K* = 6 in this paper); $f_{MLP}(•)$ is the mapping function implemented by the multi-layer perceptron; $s_{t}$ stands for the state vector at time step *t*; σ refers to the trainable parameters of the MLP network, including weight matrices and bias vectors of all fully connected layers, which are updated by op*t*imizing the loss function of supervised learning.

The loss function for supervised learning is defined as:


$$\begin{array}{c} L_{sup}=\frac{\text{1}}{N}\sum_{n=1}^{N}{\|{\hat{y}}_{n}-y_{n}^{\text{BPA}}\|}_{\text{2}}^{\text{2}} \end{array}$$ (69)

Where $L_{sup}$ denotes the loss value of supervised learning; *N* is the total number of training set samples; ${\hat{y}}_{n}$ represents the model-predicted risk vector of the *n*-th sample; $y_{n}^{\text{BPA}}$ is the PSD-BPA simulated risk label corrsponding to the *n*-th sample; ${\|•\|}_{\text{2}}^{\text{2}}$ stands for the squared Euclidean norm, which calculates the sum of squared element-wise errors between predicted values and reference labels. The training objective is to minimize this loss function.

To prevent temporal information leakage, the dataset must be split into mutually exclusive training, validation and test sets on the basis of complete 72-hour scenarios rather than individual hourly samples. The exact number of scenarios in each subset and the random seed are reported for reproducibility.

A total of 1600 complete 72-hour risk scenarios are generated in this work, which are stratified and partitioned at the scenario level: 1120 scenarios for the training set (70%), 240 for the validation set (15%), and 240 for the independent test set (15%). The training set consists of 560 winter snowstorm scenarios and 560 high-temperature stagnant weather scenarios; both the validation and test sets contain 120 winter snowstorm cases and 120 high-temperature stagnant weather cases respectively. Winter snowstorm scenarios cover winter peak and winter valley operation modes, while high-temperature stagnant weather scenarios include summer peak and summer valley operation modes. Each operation mode involves five fluctuation intensity levels of σ=−20%、 − 10%、0%、10%、20% and the stratified proportion is kept consistent across the three datasets. The random seed for dataset partitioning is set to 2026.

Prediction accuracy is quantified by the error between the model-estimated risk values and PSD-BPA reference labels on the independent test set. The main evaluation metrics are listed as follows:


$$\begin{array}{c} \text{MAE}=\frac{\text{1}}{NK}\sum_{n=1}^{N}\sum_{k=1}^{K}\left|{\hat{y}}_{n,k}-y_{n,k}\right| \end{array}$$ (70)

Where MAE stands for Mean Absolute Error, which quantifies the average deviation magnitude between predicted values and ground-truth values. *N* is the total number of scenarios in the test set (*N* = 240) in this paper); *K* denotes the number of risk indicator categories (*K* = 6 in this paper). ${\hat{y}}_{n,k}$ represents the predicted value of the *k*-th type of risk for the *n*-th scenario, and $y_{n,k}$ is the corresponding PSD-BPA reference label. MAE assigns equal weights to all errors and reflects the overall prediction performance of the model; a value closer to 0 indicates higher prediction accuracy.


$$\begin{array}{c} \text{RMSE}=\sqrt{\frac{\text{1}}{NK}\sum_{n=1}^{N}\sum_{k=1}^{K}({\hat{y}}_{n,k}-y_{n,k}{)}^{\text{2}}} \end{array}$$ (71)

Where RMSE refers to Root Mean Square Error. It amplifies the weight of large errors via squaring operation and is more sensitive to outliers. If RMSE is remarkably larger than MAE, it indicates that the model yields a small number of predictions with substantial deviations and the error distribution is non-uniform. This metric is adopted to evaluate the model performance under scenarios with extreme prediction biases.


$$\begin{array}{c} R^{\text{2}}=1-\frac{\sum_{n=1}^{N}\sum_{k=1}^{K}(y_{n,k}-{\hat{y}}_{n,k}{)}^{\text{2}}}{\sum_{n=1}^{N}\sum_{K=1}^{K}(y_{n,k}-{\overset{―}{y}}_{k}{)}^{\text{2}}} \end{array}$$ (72)

Where *R*^2^ denotes the Coefficient of Determination, which measures the degree to which the model explains the total variance of the dataset. The numerator $\sum_{n=1}^{N}\sum_{k=1}^{K}(y_{n,k}-{\hat{y}}_{n,k}{)}^{\text{2}}$ is the Sum of Squared Residuals (SSR), representing the unexplained variance component by the model. The denominator $\sum_{n=1}^{N}\sum_{K=1}^{K}(y_{n,k}-{\overset{―}{y}}_{k}{)}^{\text{2}}$ refers to the Total Sum of Squares (SST), which characterizes the total data variance referenced to the sample mean. $\overset{―}{y}k\;=\frac{\text{1}}{N}\;\sum_{n=1}^{N}y_{n,k}$ is the mean value of the *k*-th risk indicator on the test set. The value range of *R*^2^ is a A value closer to 1 indicates stronger explanatory power of the model for data variance. *R*^2^ = 0 means the model achieves equivalent prediction performance to simple mean-value prediction, while *R*^2^ < 0 implies the model performs worse than the mean baseline prediction.

The 95th percentile of absolute error (P95AE) is additionally reported to describe the tail error of model predictions:


$$\begin{array}{c} \text{P95AE}={\text{Percentile}}_{95}\left(\left\{\left|{\hat{y}}_{n,k}-y_{n,k}\right|,\forall n=1,\text{...},N,\forall k=1,\text{...},K\right\}\right) \end{array}$$ (73)

Where P95AE denotes the 95th percentile of absolute error, which quantifies tail errors and reflects the model’s prediction performance under the worst 5% cases. ${\text{Percentile}}_{95}(•)$ represents the 95th-percentile operator, which takes the value at the 95% position after sorting all *N* × *K* absolute errors in ascending order. In power system risk assessment, extreme yet rare high-risk scenarios usually lead to the most severe consequences. P95AE can effectively characterize the model’s prediction capability at “critical moments”, making it one of the core indicators for engineering safety evaluation.

Among the above metrics, MAE, RMSE, *R*^2^ and P95AE are all calculated on the independent test set. The cumulative reward is only adopted to reflect training convergence and stability, and shall not be regarded as a substitute metric for out-of-sample prediction accuracy on the independent test set.

## 6. Case analysis

### 6.1. System introduction

In a 2025 power grid planning scheme for Northeast China, there are 637 transmission lines at 500 kV and 3,478 lines at 220 kV, a total of 4,788 high voltage substations, and an installed power capacity of 277,200 MW (with conventional hydropower and pumped storage, nuclear power, thermal power, renewable energy, and energy storage accounting for 5.13%, 2.41%, 40.03%, 49.61%, and 2.83%, respectively). The forecasted peak load is 93,800 MW. The system operation boundary parameters, set with reference to current design specifications for various types of equipment and relevant literature, are shown in Table 3.

**Table 3:** Values for each type of operating parameter.

ParameterValueRemarks	ParameterValueRemarks	ParameterValueRemarks
Design wind speed for transmission lines	23.5 m/s	220kV
	27 m/s	500kV
Design ice thickness for transmission lines	10 mm	Light ice zone
	20 mm	Medium ice zone
	40 mm	Heavy ice zone
Design temperature for transmission lines	40°C/0°C	Icing temperature conditions
Design temperature for transformers	31°C	N/A
Design wind speed for wind turbines	20 m/s	Cut-out wind speed
	3 m/s	Cut-in wind speed
Design low-temperature protection value for wind turbines	−28°C	Low-temperature type
Load increment with temperature	30°C/-5°C	Load significant increase factor

### 6.2. Extreme scenario generation

The duration of extreme weather is set to 3 days, with a simulation time step of 1 hour. For each confidence value σ, $M^{\prime }\text{T}$ risk scenarios are generated corresponding to two types of extreme weather conditions, where $M^{\prime }=m_{\text{1}}+m_{\text{2}}$, and $m_{\text{1}}$ and $m_{\text{2}}$ are the numbers of states per time step under extreme weather conditions I and II, respectively. The composition of these scenarios is illustrated in Fig 3. Taking Fig 3(a) and 3(b) as examples, each column represents the temporal evolution of the system state under a given extreme weather condition, while each row represents different sampling outcomes at a specific time step due to different extreme weather conditions.

**Fig 3:**
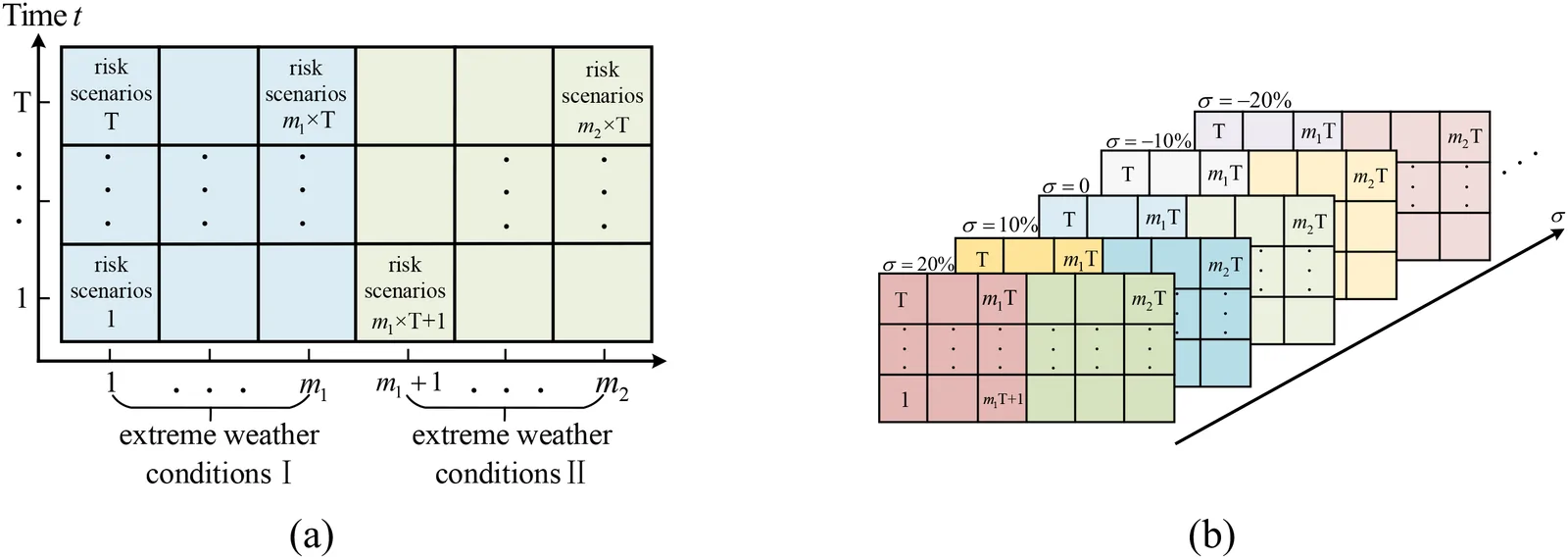
The Composition of risk scenarions.

Table 4 presents the meteorological factors when the confidence value is 10%. The corresponding 72-hour variation curves of the extreme weather conditions are shown in Fig 4. The calculated occurrence probabilities of the two types of weather events are 1.905 × 10^−4^ and 1.014 × 10^−4^, respectively. The time varying failure probabilities of the three types of equipment are illustrated in Figs 5 and 6.

**Table 4:** Values of various extreme meteorological factors.

Extreme Meteorological Conditions	Risk Scenario Set	Confidence Valueσ	Extreme Meteorological Factors		
			Temperature (°C)	Wind Speed(m/s)	Snowfall(mm)
Condition I	$\left[\text{1},\;m_{\text{1}}T\right]$	10%	<−4.5	Condition I	$\left[\text{1},\;m_{\text{1}}T\right]$
Condition II	$\left[m_{\text{1}}T+\text{1},\;m_{\text{2}}T\right]$	10%	>36	Condition II	$\left[m_{\text{1}}T+\text{1},\;m_{\text{2}}T\right]$

**Fig 4:**
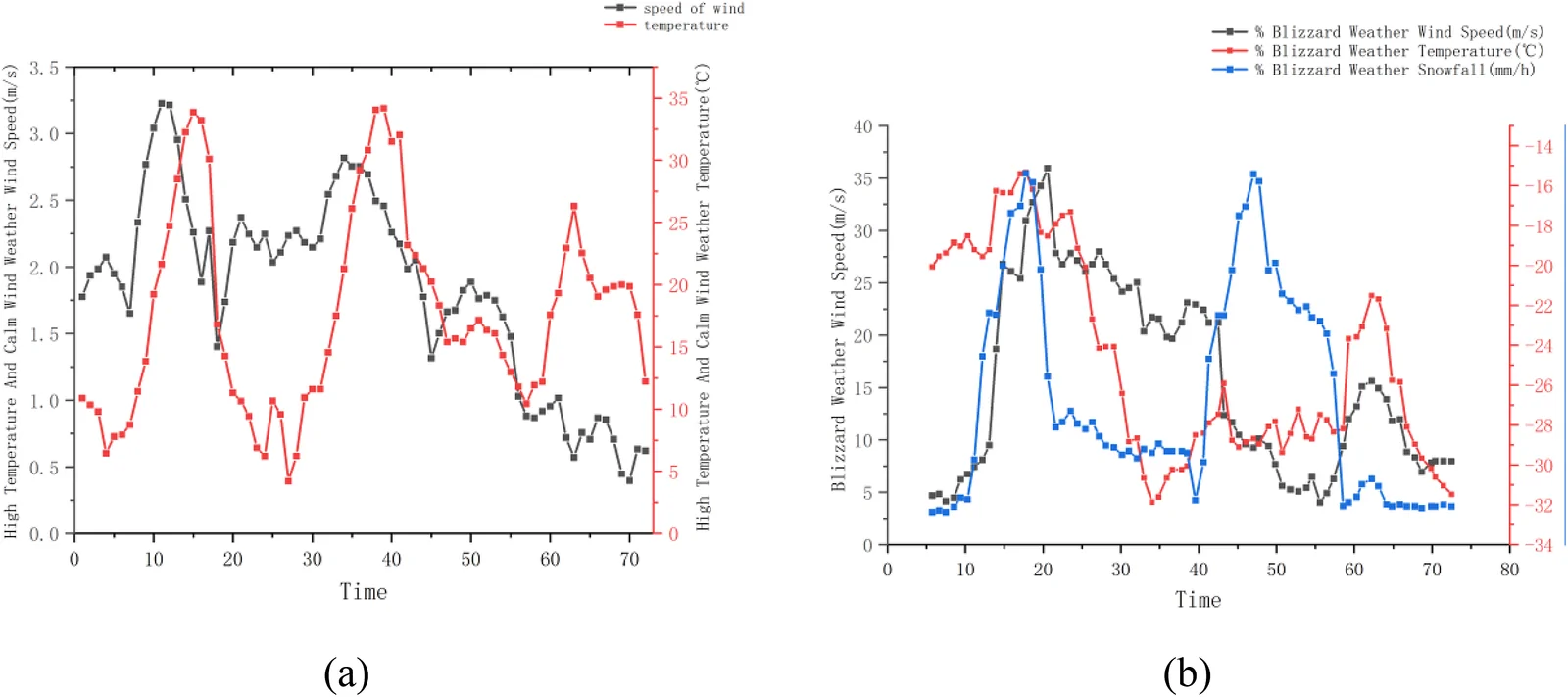
72 h extreme weather data for risk scenarios in the Northeast of China.

**Fig 5:**
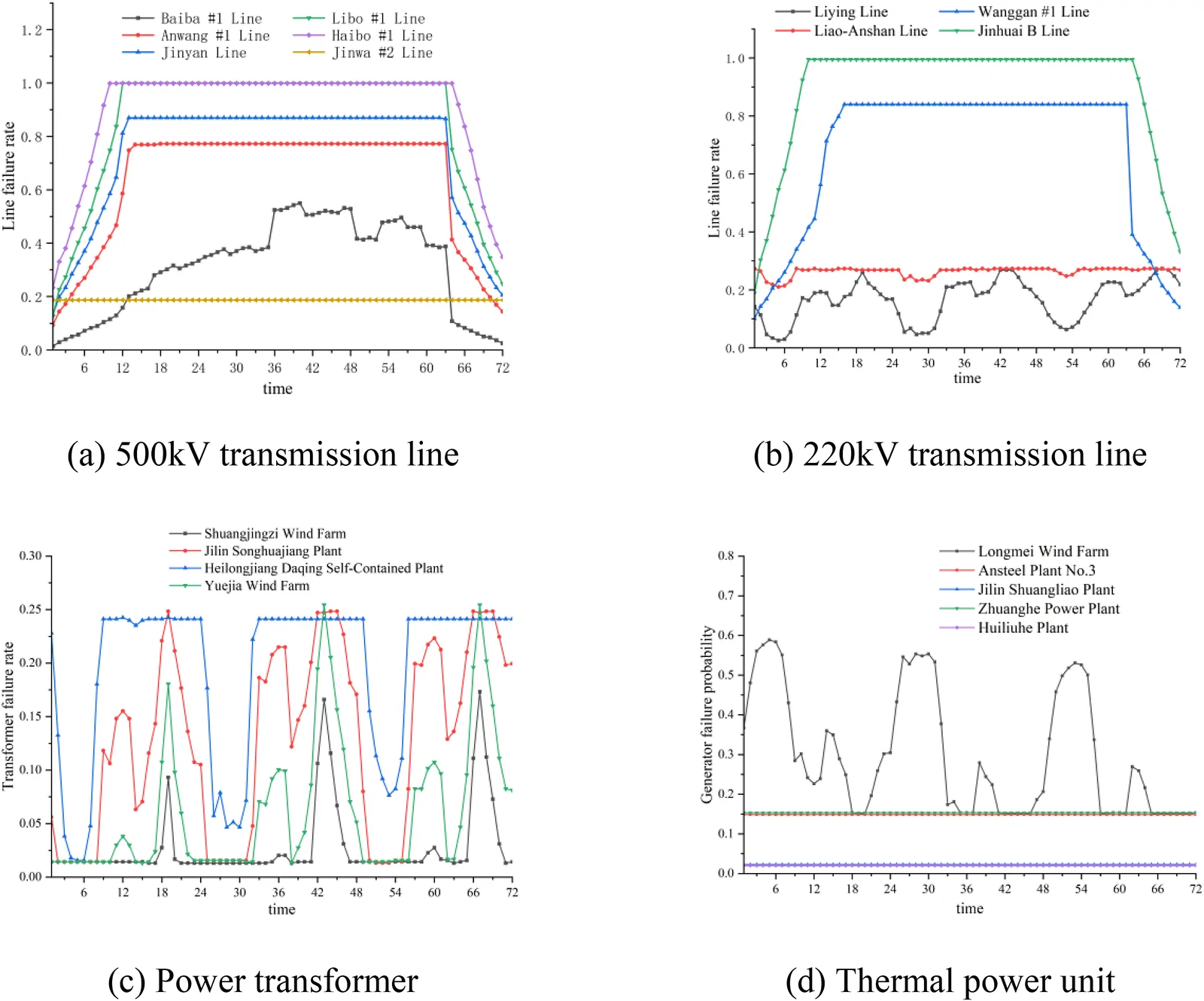
Probability of equipment failure in the Northeast Power Grid under stormy weather.

**Fig 6:**
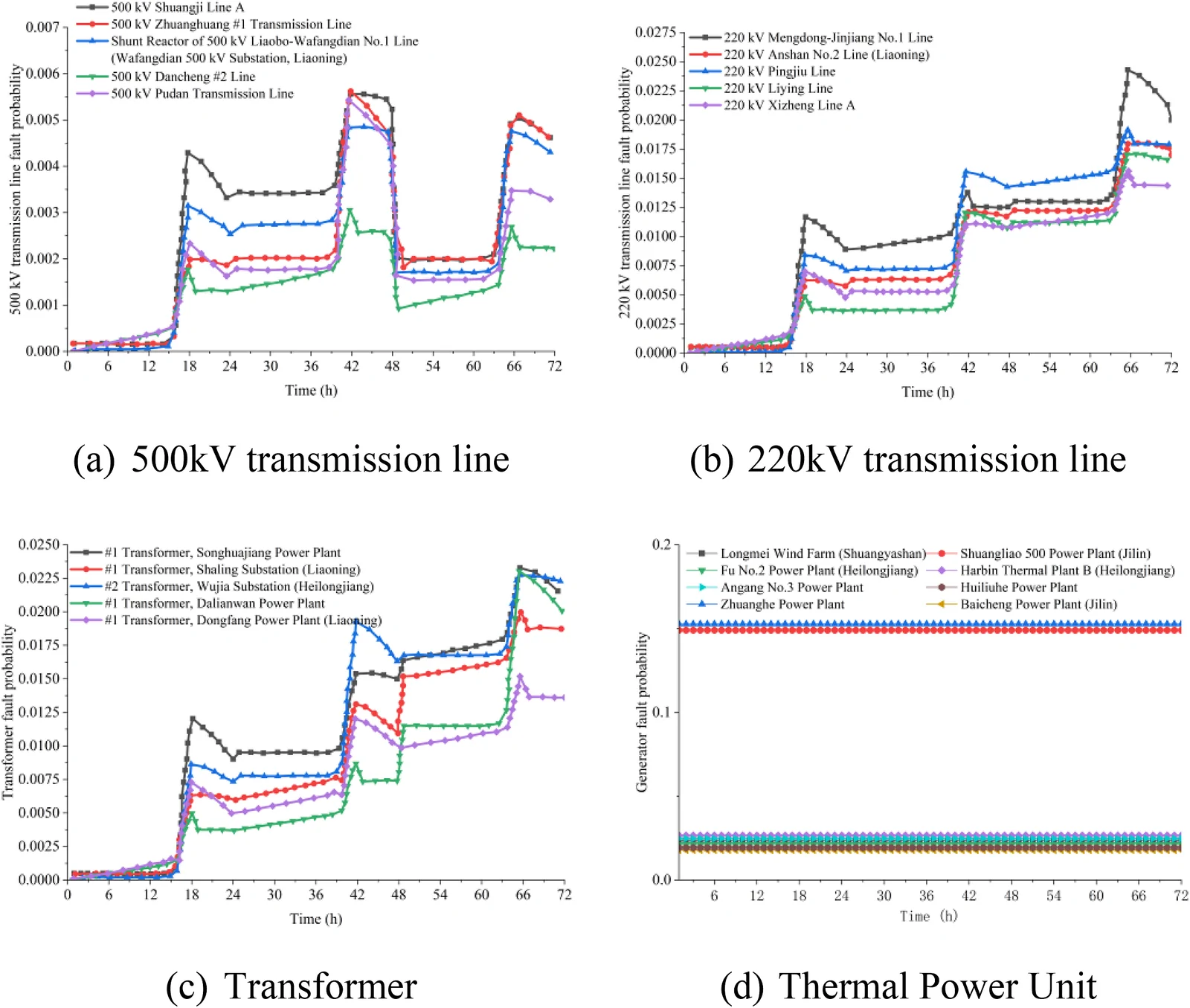
Probability of equipment failure in the Northeast Power Grid under hot-windless weather.

### 6.3. Risk Assessment result analysis

#### 6.3.1. Evaluation results of system risk level.

In this section, the risk variations of adequacy and flexibility indicators under the extreme weather conditions corresponding to a confidence value of 10% are evaluated for Heilongjiang Province, Jilin Province, Liaoning Province, and the Eastern Inner Mongolia region (hereinafter referred to as the three provinces and one region). The risk of power inadequacy is shown in Fig 7. The energy inadequacy risk values for the three provinces and one region are 1.98 × 10^−3^, 9.56 × 10^−4^, 4.8 × 10^−3^, and 9.3 × 10^−6^, respectively.

**Fig 7:**
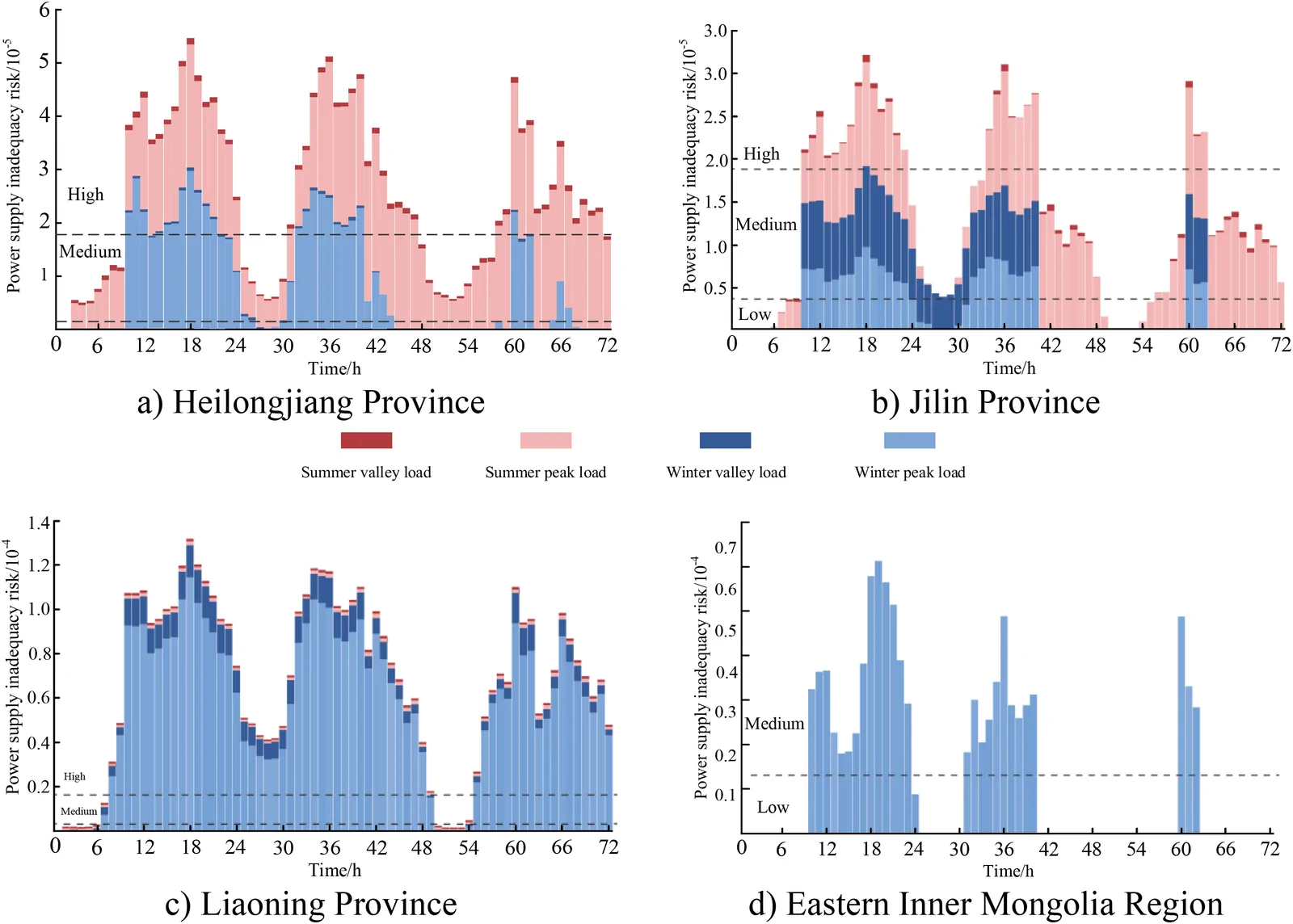
Power shortage risk of various regions of China in various system operation modes.

#### 6.3.2. Analysis of system risk level results.

According to the risk classification criteria for power systems in the literature [24], three risk levels are defined: a risk level of ${\text{R}}_{\text{o},\text{t}}>\text{1}.\text{79}\times {\text{10}}^{-\text{5}}$ is classified as high risk; $\text{2}.\text{39}\times {\text{10}}^{-\text{6}}<{\text{R}}_{\text{o},\text{t}}\leq \text{1}.\text{79}\times {\text{10}}^{-\text{5}}$ as medium risk; and ${\text{R}}_{\text{o},\text{t}}\leq \text{2}.\text{39}\times {\text{10}}^{-\text{6}}$ as low risk. Based on this, the proportions of time (referred to as the high-risk proportion, medium-risk proportion, and low-risk proportion, respectively) during which various risk values in the three provinces and one region remain at high, medium, and low risk levels throughout the duration of the extreme weather event, as well as their average values, are obtained and shown in Tables 4–6.

**Table 5:** High-risk proportion of various indicators in various regions of China.

Risk Indicator	High Risk Proportion by Region/%				Average Proportion/%
	Heilongjiang Province	Jilin Province	Liaoning Province	Eastern Inner Mongolia Region	
Power Shortage	65.27	33.33	81.94	0	45.14
Insufficient Spinning Reserve	100	88.89	100	38.89	81.94
Voltage Violation	97.22	12.5	83.5	0	48.31
Power Flow Violation	98.61	41.66	87.5	0	56.94
Transformer Load Rate Violation	98.61	38.88	90.5	0	57
Frequency Violation	30.56	4.167	41.67	0	19.1
Comprehensive Risk	82.41	37.73	81.19	6.48	

**Table 6:** Proportion of Medium Risk of Different Indicators in Each Region.

Risk Indicator	Proportion by Different Regions/%			
	Heilongjiang Province	Jilin Province	Liaoning Province	Inner MongoliaRegion
Power shortage	31.94	48.61	4.17	0
Spinning reserve	0	1.39	0	0
Overvoltage	1.39	13.89	0	0
Overcurrent	0	15.28	0	0
Transformer overload	0	16.67	0	0
Frequency limit	62.5	70.8	40.28	0
Overall risk	16.2	28	7.41	0

1) Risk region identification

To identify the risk situation of the three provinces and one region, the comprehensive regional risk is divided into three levels: a region with a high-risk proportion of the comprehensive indicator exceeding 70% is classified as a high-risk region; a proportion between 30% and 70% corresponds to a medium-risk region; and a proportion below 30% corresponds to a low-risk region.

As shown in Table 4, except for the insufficient spinning reserve risk which exhibits high values in a few time periods, all other indicators in the Eastern Inner Mongolia region remain at low risk levels, indicating good grid adequacy and security and stability. The high-risk proportion of the comprehensive risk value is only 6.48%. Therefore, this region is identified as the most robust planned grid and is classified as a low-risk region, which can be excluded from subsequent analysis. For Jilin Province, except for the insufficient spinning reserve risk, all other risk indicators remain at low-to-medium risk levels, and the high-risk proportion of the comprehensive risk value is 37.73%, corresponding to a medium-risk region. For Heilongjiang Province and Liaoning Province, most risk values in both provinces are at high risk levels, with high-risk proportions of the comprehensive risk value being 82.41% and 81.19%, respectively. Furthermore, as shown in Fig 7, all risk values in Liaoning Province are several times higher than those in Heilongjiang Province, making it the most vulnerable region in the future Northeast China power grid.

2) Comparative analysis of risk indicators

This section analyzes the magnitudes of various risk indicators. From the distribution ranges of the data in each row of Tables 6, and 7, it can be seen that for the flexibility indicator, all insufficient spinning reserve risk values in the current planned grids of the three provinces and one region exhibit periods at high risk levels, with the corresponding average high-risk proportion reaching as high as 81.94%. The problem of insufficient flexibility is severe, and planning schemes adapted to extreme scenarios should be developed taking into account the risk of system flexibility inadequacy. Regarding security and stability indicators, except for the frequency violation indicator, the average high-risk proportions of other security and stability risk values in Heilongjiang Province and Liaoning Province are all greater than 48%. For the adequacy inadequacy indicator, the average high-risk proportion of this indicator across the three provinces and one region is 45%. Therefore, insufficient system flexibility is the most common and severe risk faced by the entire Northeast China power grid, followed by insufficient security and stability, with insufficient adequacy being the lowest.

**Table 7:** Proportion of Low Risk for Different Indicators in Each Region.

Risk Indicator	Proportion by Different Regions/%			
	Heilongjiang Province	Jilin Province	Liaoning Province	Inner Mongolia Region
Power shortage	2.78	18.06	13.89	100
Spinning reserve	0	9.72	0	61.11
Overvoltage	1.39	73.61	12.5	100
Overcurrent	1.39	43.06	12.5	100
Transformer overload	1.39	44.44	12.5	100
Frequency limit	6.94	25	18.06	100
Overall risk	2.31	35.65	11.57	93.52

3) Risk evolution process and cause analysis under different extreme weather conditions

The variations of various power system risks are closely related to the evolution of extreme weather. By observing the changes in extreme weather meteorological factors shown in Fig 4, the changes in equipment failure probabilities shown in Figs 5 and 6, and Fig 7, the system performance variation during the duration of extreme weather can be analyzed. Taking the winter blizzard impact process as an example, the trend of system performance within a day can be divided into the following three stages:

(1) Stage I: In the early stage of winter blizzard impact (00:00–09:00), snowfall/wind speed is relatively low, wind turbines are not completely cut out, and the increase in electric heating load is not significant. The weather impact on the system is not severe, and the system can still meet load demand as much as possible; therefore, various risk values remain at low levels.
(2) Stage II: During the period from 10:00–21:00, as weather conditions worsen (snowfall increases from 0.2 mm/h to 1.6 mm/h, wind speed increases from 13 m/s to 34 m/s, temperature drops from −15°C to −30°C), the source–grid–load sides of the system are all affected to varying degrees (on the source side, most wind turbines are cut out, and output drops sharply; on the grid side, the failure rate of some lines increases from 0.2 to 1.0; on the load side, the electric heating load increases to 1.1 times its forecasted value). System performance is significantly degraded, and various risk values gradually increase from low-to-medium risk levels to high risk levels, peaking during high-load periods such as 10:00–13:00 and 17:00–19:00 as load fluctuates.
(3) Stage III: During the period from 22:00–24:00, load decreases and snowfall reduces, leading to lower component failure rates, and various risks decline.

In addition, the system performance variation during summer high-temperature weather is similar to the above process. In Stage II, as temperature rises from 26°C to 37°C and wind speed decreases to 0 m/s, the power deficit between the source and load sides continues to increase, and various system risks peak during high-load periods (20:00–22:00). However, because the increase in the failure rate of lines/transformers and other equipment caused by continuous high temperatures is much smaller than that under blizzard conditions, with a maximum value of only 0.0055, the number of grid faults is relatively low, and the main source of system risk depends on power supply adequacy.

4) Analysis of regional risk conditions under different operating modes

To analyze the differences in the main risks faced by the system under different operating modes, this section analyzes the risk conditions of each region under the winter peak, winter valley, summer peak, and summer valley operating modes.

Table 8 shows the capacity ratios of conventional units (thermal/hydro/nuclear/pumped storage) and wind turbine units under different operating modes in the 2025 Northeast China power grid planning scheme.

**Table 8:** Percentage of startup capacity of conventional units and wind turbines in different operation modes in various locations.

Unit Type	Operation Mode	Proportion by Different Regions/%			
		Heilongjiang Province	Jilin Province	Liaoning Province	Inner Mongolia Region
Conventional Unit	Winter Peak	77.49	51.63	82.84	75.67
	Winter Off‑peak	60.90	54.34	75.97	70.25
	Summer Peak	77.86	50.47	82.5	78.91
	Summer Off‑peak	73.35	48.69	78.5	71.8
Wind Turbine Unit	Winter Peak	22.51	41.63	17.16	25.32
	Winter Off‑peak	39.1	53.37	25.03	30.75
	Summer Peak	22.14	49.54	17.5	22.09
	Summer Off‑peak	26.65	51.31	22.8	29.2

Observing the proportion of risk values in different regions under various operating modes shown in Fig 7, it can be found that most provinces exhibit the following characteristics for various risk indicators: the risk proportions under the two types of weather conditions are comparable, and under the same weather condition, the risks corresponding to the heavy-load operating mode are much higher than those under the light-load operating mode. Taking winter as an example, the reasons for the above characteristics are as follows. In the winter peak mode, the capacity ratio of wind turbine units is only about 20%, while that of thermal power units is 50%–90%, indicating that system power supply mainly depends on thermal power. When a severe fault occurs in the system, the limited upward regulation capacity of thermal power leads to a large supply–demand deficit, and various risk levels are relatively high. In the winter valley mode, the capacity ratio of wind turbine units is 20%–60%. Although blizzard weather may cause large scale disconnection of wind turbines, the load under this mode is relatively small, and the thermal power units in the minimum operating mode can still meet most of the load demand.

In particular, the adequacy and flexibility risks of the Liaoning power grid are high in winter (86.5% in winter peak mode and 10% in winter valley mode), but low in summer (2% in summer peak mode and 1.5% in summer valley mode). This is because the main causes of system risk under the two types of extreme weather conditions are different: the former (summer high-temperature and windless weather) is caused only by insufficient power supply adequacy, while the latter (winter blizzard) is caused by the combined effects of insufficient power supply adequacy and equipment failures. Specifically, the capacity ratio of thermal power units in the Liaoning power grid under various operating modes (approximately 80%) is higher than that in other regions. Under high-temperature and windless weather, due to the low equipment failure rate, the system experiences few faults, and various risks are low. However, under blizzard weather, the weather conditions and heavy loads lead to a high equipment failure rate, causing severe system faults. If the system is in the winter valley mode, the conventional units in the minimum operating mode can barely meet the power demand. If the system is in the winter peak mode, the conventional units are already operating near full capacity, with an output ratio of 98.3%, leaving almost no upward regulation capability to meet the large load demand under blizzard weather. Therefore, the risk in the winter peak mode is high, representing the main risk among the four operating modes.

In addition, the security and stability risks, such as voltage violation, in the Jilin power grid are more prominent under the winter valley operating mode. Unlike other provinces, Jilin is relatively rich in wind power resources, and its capacity ratio of wind turbine units under the four operating modes (approximately 45%) is higher than that in other regions, reaching as high as 53% under the winter valley mode. Under these conditions, when blizzard weather causes large-scale disconnection of wind turbines, the thermal power units are in the minimum operating mode and cannot provide sufficient active and reactive power in a short time, resulting in a larger supply–demand deficit. Consequently, the security and stability risks such as voltage violation and power flow violation become more prominent in the system.

#### 6.3.3. Performance analysis of evaluation method.

To validate the proposed models and methods in this paper, the following three evaluation calculation schemes were designed, and simulations were carried out for each. The results are presented in Table 9 and Table 11. The energy inadequacy risks under different confidence values are shown in Table 10.

**Table 9:** High-risk proportion of various indicators in various regions of China with different values of in scheme 1.

Risk type	Confidence value σ/%	Proportion of high-risk areas by region/%			
		Heilongjiang	Jilin	Liaoning	Eastern Inner Mongolia
Power deficiency	−20	2.78	0	11.11	0
	−10	63.89	26.39	65.28	0
	0	65.28	33.33	68.94	0
	10	72.37	45.76	75	1.09
	20	77.41	50.28	80	3.51
Insufficient spinning reserve	−20	11.11	0	80.56	0
	−10	81.94	62.5	100	0
	0	100	88.89	100	38.89
	10	100	100	100	55.56
	20	100	100	100	58.06
Voltage violation	−20	0	0	11.11	0
	−10	62.5	0	68.06	0
	0	97.22	12.5	87.5	0
	10	98.61	50	98.61	3.09
	20	88.89	54.44	100	9.51
Power flow violation	−20	0	0	11.11	0
	−10	62.5	0	68.06	0
	0	98.61	41.67	87.5	0
	10	98.61	52.78	100	0
	20	98.83	58.78	100	0
Transformer loading violation	−20	0	0	11.11	0
	−10	62.5	0	68.06	0
	0	98.61	38.89	87.5	0
	10	98.61	65.28	100	0
	20	95.83	72.78	100	0
Frequency violation	−20	0	0	4.17	0
	−10	21.35	2.78	34.72	0
	0	30.56	4.167	41.67	0
	10	34.72	11.11	43.67	0
	20	38.44	15.11	51.75	2.81

**Table 10:** Risk values of power shortages by region at different σ.

Risk type	Confidence value σ	Heilongjiang	Jilin	Liaoning
Power deficiency	−20%	1.39 × 10−4	0	1.5 × 10−3
	−10%	2.77 × 10−3	0	7.24 × 10−3
	0	1.88 × 10−3	9.56 × 10−4	4.8 × 10−3
	10%	5.39 × 10−3	4.28 × 10−4	5.77 × 10−3
	20%	6.7 × 10−3	5.09 × 10−3	7.55 × 10−3

Scheme 1: The proposed models and methods were used to conduct a comparative analysis of the system risk levels under extreme conditions corresponding to different confidence values σ.

Scheme 2:Using historical weather data of a certain rainstorm event as input, and adopting the direct failure model of equipment under rainstorm disasters from reference [25] for the environment-dependent failure probability, while keeping the other models as those proposed in this paper, the system risk was assessed.

Scheme 3: Risk scenario uncertainty models were established by considering only the source–load power fluctuations and only the equipment failures, respectively, and the system risk under partial uncertainty was calculated accordingly.

1) Superiority analysis of the proposed assessment method

Observing Table 9, it can be seen that as the confidence value σ increases, the weather becomes more severe, and the risk values of various indicators also increase. When σ increases to 0, the system is exactly at the critical state of abnormal operation, and the high-risk proportion of each indicator increases significantly, indicating that under such extreme conditions, the system is indeed a high-risk scenario. This further verifies that the extreme weather conditions defined based on the normal operating state threshold of the grid, as proposed in this paper, can indeed provide relatively accurate criteria for risk scenario identification in power grids. Moreover, this method has profound implications: by fitting the functional relationship between meteorological elements of different severity levels and the corresponding system operational risks, the risk level of a planned power grid under future extreme weather can be estimated merely by monitoring meteorological factors. This means that rapid identification of extreme weather scenarios and early warning of risks in power grids become feasible.

2) Accuracy analysis of the proposed assessment method

Extreme rainstorms are not included in the results of risk scenario identification in this paper. Therefore, simulations were carried out using historical data of a rainstorm event, and the results are shown in Table 11. Since no fault modeling of grid equipment under rainstorm weather was performed in this paper, and this case was merely used as a control group in the numerical example, the gridside uncertainty was characterized using the grid equipment fault model for rainstorm disasters from reference [25]. From Table 11, it can be seen that the high-risk proportions of various system indicators obtained by Scheme 1 are significantly higher than those obtained by Scheme 2 by tens of times. This indicates that the two types of extreme weather identified and generated for the Northeast China region in this paper can indeed cause considerable impacts on the power system of this region, whereas the un identified rainstorm poses a relatively low threat to the regional grid. Therefore, the scenario identification method proposed in this paper is effective. By fully considering regional climate and grid characteristics, the proposed method can provide adaptive scenario data for risk assessment of the target power grid, thereby helping to improve the accuracy of each calculated risk value.

**Table 11:** Risk values of power shortages by region at different σ.

Scheme	Confidence valueσ/%	Weather data source	Sources of uncertainty		Consequence evaluation platform	Proportion of high-risk areas by region/%				
			Source and load	Grid side		Power deficiency	Insufficient spinning reserve	Voltage violation	Power flow violation	Transformer loading violation
1	0	Scenario generation	√	√	Planning platform	68.94	100	87.5	87.5	87.5
2		Historical heavy rain	√	Reference [34]		6.07	6.41	2.38	2.59	2.81
3	0	Scenario generation	√	×		66.88	98.35	79.19	80.13	78.96
			×	√		62.74	90.2	86.28	84.44	84

Compared with Scheme 1, the high-risk proportions of various system indicators in Scheme 3 are reduced to varying degrees, with a reduction range of 2% to 9%. This indicates that risk assessment results considering only source load power/grid side failure uncertainties are overly optimistic. It can be seen that comprehensively and accurately characterizing source grid load uncertainties plays an important role in risk assessment work.

### 6.4. Comprehensive analysis of algorithm accuracy and computational efficiency

To fully validate the comprehensive performance of the proposed DDPG-Attention method in terms of evaluation accuracy and computational efficiency, this section conducts analyses from two dimensions as follows: (1) Evaluation accuracy comparison with the supervised MLP baseline and other deep reinforcement learning algorithms; (2) Computational efficiency comparison with the traditional time-sequential Monte Carlo simulation implemented by PSD-BPA.The former is adopted to verify the modeling advantages of the attention mechanism and reinforcement learning framework for the risk assessment task, whereas the latter quantifies the acceleration gain of the proposed approach for real-time engineering deployment.

#### 6.4.1. Comparison with supervised learning baselines.

DDPG-Attention, DDPG, SAC, PPO, DQN and the supervised MLP baseline adopt identical normalized input features and PSD-BPA reference labels. The reinforcement learning algorithms follow the state space, action space, state transition rules and reward function defined in Section 5.1, while the MLP minimizes the loss function formulated in Equation (69). All prediction errors are computed on the same independent scenario-level test set.

Fig 8 illustrates the average cumulative reward over 1000 training episodes. The curve of DDPG-Attention rises more rapidly from approximately −8 to nearly 0.2 compared with other RL baselines. This phenomenon indicates that the proposed attention-augmented DDPG achieves a faster optimization rate under the predefined reward function. Nevertheless, the cumulative reward curve alone cannot serve as valid evidence for superior out-of-sample prediction accuracy.

**Fig 8:**
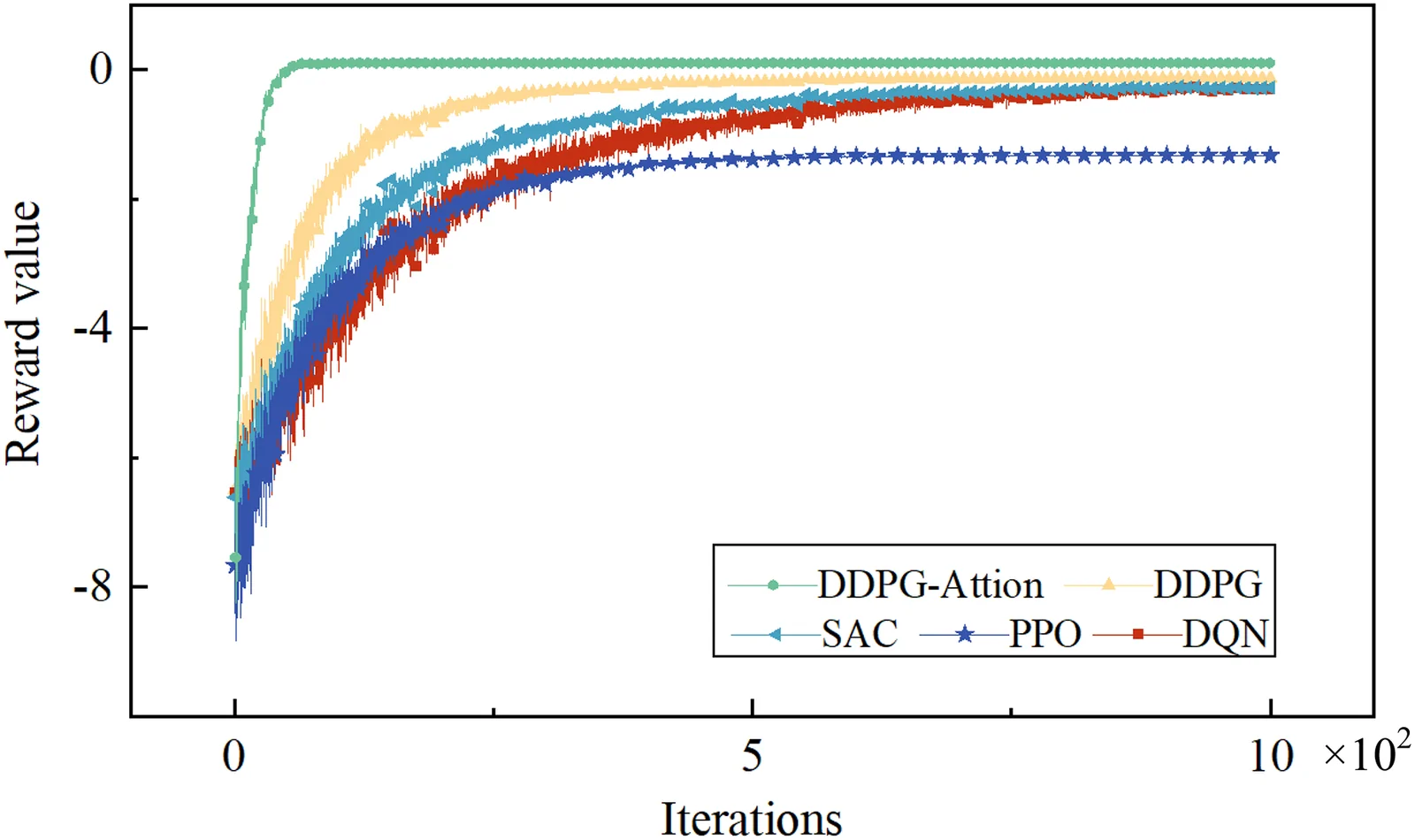
Comparative Analysis of Different Algorithms.

As shown in Fig 8, the proposed DDPG-Attention algorithm significantly outperforms the other benchmark algorithms in terms of both convergence speed and final reward value. Specifically, in the early stage of training, the average reward of DDPG-Attention rapidly increases from −8 to approximately 0.2, whereas the convergence speeds of the original DDPG, SAC, PPO, and DQN are relatively slower, and their reward values under the same number of training episodes are all lower than those of the proposed algorithm. This improvement is attributed to the introduction of the attention mechanism, which enables the agent to adaptively focus on the features most relevant to the power balance risk under extreme weather conditions in a high dimensional state space, thereby accelerating the learning process of the value function in the policy network.

The prediction errors on the independent test set are summarized in Table 12. All error metrics are calculated within the normalized risk indicator space ranging from 0 to 1, with 240 complete 72-hour scenarios adopted as the independent test dataset. The proposed DDPG-Attention achieves the minimum MAE, RMSE and P95AE values as well as the maximum *R*^2^, which demonstrates that the feature token-based attention mechanism can improve the fitting accuracy of multiple risk indicators and enhance the control ability against extreme prediction errors.

**Table 12:** Accuracy and Inference Time Comparison on Independent Test Set.

Model	MAE	RMSE	*R*²	P95AE	Average Inference Time per Single Scenario(s)
MLP	0.0396	0.0495	0.925	0.0972	0.041
DQN	0.0553	0.0692	0.853	0.1352	0.078
PPO	0.0479	0.0600	0.889	0.1177	0.113
SAC	0.0420	0.0526	0.915	0.1027	0.105
DDPG	0.0381	0.0478	0.930	0.0936	0.091
DDPG-Attention	0.0310	0.0389	0.954	0.0760	0.120

As can be observed from Table 12, the proposed DDPG-Attention outperforms all baseline algorithms significantly in all four accuracy metrics. Specifically, in terms of overall prediction accuracy, the MAE and RMSE of DDPG-Attention reach 0.0310 and 0.0389, respectively. Compared with the best-performing baseline DDPG (0.0381 and 0.0478), they are reduced by approximately 18.6% and 18.5%; compared with the supervised learning MLP (0.0396 and 0.0495), the reductions are about 21.7% and 21.4%. These results reveal that the embedded attention mechanism can effectively capture critical spatio-temporal features strongly correlated with power balance risks, while the reinforcement learning framework further strengthens the model’s capability to characterize the temporal dependencies of risk evolution.

In terms of goodness-of-fit, the *R*^2^ of DDPG-Attention is 0.954, higher than 0.930 of DDPG and 0.925 of MLP, demonstrating that the proposed method possesses stronger explanatory power for data variance than all competitors. Of particular note is the tail error metric P95AE: the value of DDPG-Attention is 0.0760, which is approximately 18.8% lower than DDPG’s 0.0936 and 21.8% lower than MLP’s 0.0972. This metric quantifies model performance on the 5% extreme samples with the largest prediction errors, and its remarkable improvement indicates that the proposed method can maintain favorable prediction reliability even under the most severe high-risk scenarios.

The above accuracy comparisons fully validate the superiority of DDPG-Attention for the risk assessment task. The integration of the attention mechanism and reinforcement learning framework statistically improves the overall prediction performance, and more importantly, does not degrade the predictive performance on risk tail events. This carries significant engineering value for power system safety evaluation.

#### 6.4.2. Comparison of computational efficiency.

To quantify the computational efficiency superiority of the proposed method, this section compares the running time of online inference based on the attention-augmented DDPG (DDPG-Attention) and the traditional time-sequential Monte Carlo simulation embedded in PSD-BPA. All timing tests are carried out on a unified hardware platform. The hardware configuration and practical time-consuming test scheme are presented in Table 13.

**Table 13:** Hardware Configuration and Actual Running Time Test Scheme.

Item	Value
CPU	Intel Xeon Gold 6248 2.50 GHz (20 cores, 40 threads)
GPU	NVIDIA Tesla V100 (32 GB, for deep learning inference acceleration; PSD-BPA simulation is GPU-independent)
Memory/ OS/ Software Version	64 GB DDR4/ CentOS 7.9/ PSD-BPA 4.2
Number of Warm-up Scenarios/ Test Scenarios for Repeated Measurement	10 warm-up scenarios/ 100 independent test scenarios
Average Single-scenario Time of PSD-BPA	8.6 s (statistical result from 100 repeated timing runs)
Average Single-scenario Inference Time of DDPG-Attention	0.12 s (forward propagation for each time step within the 72-hour horizon, offline training excluded)
Speedup Ratio (PSD-BPA/ DDPG-Attention)	5.16 × 106
Timing Scope	Online single-scenario evaluation; offline training and scenario generation are not included

(1) Single power flow calculation time consumption of PSD-BPA

The average time consumption for a single power flow calculation by PSD-BPA is approximately 8.6 seconds, counted from 100 repeated timing tests. This duration covers the formation of nodal admittance matrix, initial iteration of the P-Q decoupling method, main iterations of the Newton-Raphson method (convergence tolerance of 10^−6^ with an average of 15 iterations), and export of calculation results. The calculation scale corresponds to the planned power grid of Northeast China in 2025, with a total installed capacity of 277,200 MW, including 637 500 kV transmission lines, 3,478 220 kV transmission lines and 4,788 high-voltage substations. According to the scenario settings in Section 6.2, the duration of extreme stagnant weather is set as T = 72 hours with a 1-hour time step, and the total number of risk scenarios is $M^{\prime }$=1000. The traditional method requires sequential power flow calculation for each time step of every single scenario, with an estimated total running time of about 7.2 days.

(2) Inference speed comparison of deep learning models

The rightmost column in Table 12 lists the average inference time of single-scenario forward propagation (covering the full 72-hour timeline) for each deep learning model. All inference time is measured under identical hardware configurations. The MLP achieves the fastest inference speed of 0.041 s, as it only executes three fully-connected layers for forward propagation. The inference time of DQN is 0.078 s and that of DDPG is 0.091 s. Benefiting from stochastic policy and more sophisticated network architectures, SAC and PPO consume 0.105 s and 0.113 s for inference, respectively. The proposed DDPG-Attention model requires 0.120 s for inference. Although slightly slower than other deep models, it still falls within the sub-second level.

It should be clarified that the above inference time of deep learning models only accounts for online forward propagation, excluding the computational overhead of the offline training phase. The offline training is completed once before model deployment and thus not included in the online evaluation time.

(3) Speedup ratio analysis

Based on the above statistical results, the proposed method achieves a speedup ratio of 5.16 × 10^6^ times compared with the traditional time-sequential Monte Carlo simulation based on PSD-BPA, representing an acceleration of more than six orders of magnitude. Even under an ideal parallel computing condition with 1000-core parallelism and zero communication overhead, the total running time of the traditional method can be reduced to approximately 619.2 seconds, which still yields a speedup ratio of 5160, an improvement of three orders of magnitude.

Furthermore, although the single inference time of the proposed DDPG-Attention (0.120 s) increases by about 0.08 s compared with MLP (0.041 s), the accuracy gains are remarkable as demonstrated in the previous accuracy comparison: at the cost of roughly threefold inference latency, the model reduces MAE by 21.7%, P95AE by 21.8%. For power system security assessment, the losses caused by misjudgment and missed judgment of high-risk operating conditions far outweigh the millisecond-level computational delay. This well-balanced trade-off between accuracy and effciency possesses clear practical engineering value.

(4) Real-time performance evaluation

When extreme weather events occur in practice, dispatch operators need to obtain system risk situational awareness within minutes or even seconds. The PSD-BPA Monte Carlo method takes several days in serial computation or tens of minutes under ideal parallel conditions to finish the assessment, which fails to meet the requirements of online real-time evaluation and is only suitable for offline planning analysis. In contrast, the DDPG-Attention model realizes near-instantaneous risk assessment with an inference latency of only 0.120 s (<1s). It can fully satisfy the demands of online real-time evaluation of power balance risks under extreme weather and provide timely and accurate risk situational perception for dispatchers.

## 7. Conclusion

This paper constructs a rapid power balance risk assessment framework under extreme weather conditions by integrating meteorological sensitivity models, time-varying component failure probabilities, DVAE-based coupled scenario generation, time-series labels from PSD-BPA, and an attention-enhanced DDPG estimator. The revised approach explicitly defines the state space, continuous action space, state transition rules, reward function, feature token attention weights, supervised learning baselines, independent test metrics, and timing schemes. The main conclusions are summarized as follows:

(1) The attention mechanism operates on multiple feature tokens instead of a single scalar score. The derived attention matrix is normalized row-wisely, enabling the Actor network to adaptively reweight meteorological data, power source information, grid parameters, load profiles, and risk features from the previous time step along the evolution of the 72-hour scenarios. On the independent test set, the proposed DDPG-Attention achieves MAE of 0.0310, RMSE of 0.0389, *R*^2^ of 0.954 and P95AE of 0.0760. Compared with vanilla DDPG, its MAE and RMSE are reduced by 18.6% and 18.5%, respectively. In contrast to the supervised MLP baseline (0.0396, 0.0495, 0.925 and 0.0972), the MAE, RMSE and P95AE are decreased by approximately 21.7%, 21.4% and 21.8%, while *R*^2^ is improved by around 3.1%. Meanwhile, it exhibits a faster reward convergence rate.
(2) The measured online inference time of DDPG-Attention for evaluating a single scenario is approximately 0.12 s. For full time-sequential Monte Carlo simulations covering 1000 risk scenarios with a 72-hour horizon per scenario, the total computation time of the traditional method reaches around 619,200 seconds, corresponding to a speedup ratio of more than six orders of magnitude. Even under an idealized 1000-core parallel computing configuration for scenario decomposition, the acceleration still exceeds three orders of magnitude.
(3) Extreme weather exerts disparate impacts on three dimensions: power generation resources, power transmission facilities and electricity demand. Persistent stagnant weather primarily leads to long-duration insufficient renewable energy output and adequacy risks, whereas abrupt destructive weather may simultaneously raise component failure probabilities, tighten transmission constraints and trigger climate-sensitive load increments, resulting in aggravated security and stability risks. High-risk time windows mainly occur when heavy load, insufficient available generation capacity and weakened network transmission capability coincide.
(4) Regional evaluation results demonstrate that planning and operational countermeasures should be formulated in a differentiated manner according to local power supply structures and meteorological exposure characteristics. Prioritized measures include deploying flexible regulating resources, optimizing wind turbine protection and control parameters, implementing anti-icing schemes for transmission lines, cooling upgrades for transformers and reinforcement of vulnerable equipment, as well as coordinating preventive control and emergency control strategies.
