# Supplementary material for: A fast risk assessment method for power balance under extreme weather conditions based on deep reinforcement learning with a coupled attention mechanism
Source: PLoS One. 2026 Oct 8;21(10):e0359101. doi: 10.1371/journal.pone.0359101 (PMC13649036; doi:10.1371/journal.pone.0359101)
Supplement: Supplementary file 1 [file pone.0359101.s002.docx]

**Original Manuscript Number:** PONE-D-26-32244

**Original Article Title: “A Fast Risk Assessment Method for Power Balance Under Extreme Weather Conditions Based on Deep Reinforcement Learning with a Coupled Attention Mechanism**”

Dear Editor,

We would like to express our sincere gratitude to you and the reviewers for the valuable time and constructive comments devoted to our manuscript (Manuscript Number: PONE-D-26-32244). The insightful suggestions have been instrumental in helping us significantly improve the quality of our work.

We have carefully considered all the comments and have revised the manuscript accordingly. Our point-by-point responses to each comment are detailed below.

We believe the manuscript is now strengthened and hope it meets the journal's standards for publication.

Thank you for your consideration.

Best regards,

< Wang Hang > et al.

**Reviewer#1, Concern #1: The attention mechanism is only benchmarked against the original DDPG and other RL baselines (SAC, PPO, DQN); there is no comparison against a non-RL machine learning baseline (e.g., a supervised regression or standard feedforward network trained directly on the labeled risk indicator data produced by the PSD-BPA platform). Since risk indicators are computed offline for each scenario before RL training, the task in Section 5 resembles a mapping problem more than a sequential decision problem, and the manuscript should justify why a policy-gradient RL formulation (rather than direct supervised learning) is the appropriate tool, or add a supervised baseline to substantiate the choice.**

**Author response:** 我们完全认同审稿人的以下判断：在风险指标通过 PSD-BPA 平台离线计算完成后，状态特征与风险标签之间的确存在一个可被监督学习拟合的映射关系；如果将该问题简化为静态映射，前馈神经网络确实能够以更少的计算开销直接逼近该映射，并且原稿中将 DRL 作为“默认选择”而未充分论证其必要性，确实构成了方法论上的一个缺口。我们认为，审稿人的这一意见并非质疑强化学习方法的价值，而是要求我们更严格地论证：相对于更简单直接的监督学习方案，强化学习框架在本问题中究竟带来了哪些不可替代的优势？这一追问直接提升了本文方法论证的严谨性。

**Author action:**

（1）在5.1节添加MDP建模与DDPG选择依据说明：

本文将极端天气下的电力平衡风险评估建模为马尔可夫决策过程（MDP），其合理性在于：（1）极端天气的演变具有显著的时间累积效应——设备老化、冰层增长、负荷累积响应均为连续时间过程，当前时刻的风险状态依赖于前序时刻的气象条件和系统运行轨迹；（2）风险控制的决策本质上是序贯的——调度员需根据风险演化趋势动态调整预案，而非单次静态决策；（3）强化学习的“离线训练-在线推理”范式恰好满足“历史气象运行数据→实时风险快速识别”的跨场景迁移需求。尽管监督学习可直接学习状态到风险指标的映射，但其难以显式建模风险演化的时间依赖性和奖励反馈机制。因此，本文选择DDPG作为基础框架，并引入注意力机制增强其在高维状态空间中的关键特征提取能力。
（2）添加5.3节监督学习基线与独立测试指标：
The non-reinforcement learning baseline adopts feedforward MLP and is directly trained with identical PSD-BPA labels:

$\hat{y}_{t}=f_{MLP}(s_{t};\sigma)$ (68)

Where denotes the predicted risk vector at time step *t* ; *K* represents the number of risk indicator categories (*K*=6 in this paper ); is the mapping function implemented by the multi-layer perceptron; $s_{t}$ stands for the state vector at time step *t*; $\sigma$ refers to the trainable parameters of the MLP network, including weight matrices and bias vectors of all fully connected layers, which are updated by optimizing the loss function of supervised learning.

The loss function for supervised learning is defined as:

$L_{\sup}=\frac{1}{N}\sum_{n=1}^{N} \left\|\hat{y}_{n}-y_{n}^{\text{BPA}}\right\|_{2}^{2}$ (69)

Where $L_{\sup}$ denotes the loss value of supervised learning; *N* is the total number of training set samples; $\hat{y}_{n}$ represents the model-predicted risk vector of the *n*-th sample; $y_{n}^{\text{BPA}}$ is the PSD-BPA simulated risk label corrsponding to the *n*-th sample; stands for the squared Euclidean norm, which calculates the sum of squared element-wise errors between predicted values and reference labels. The training objective is to minimize this loss function.

To prevent temporal information leakage, the dataset must be split into mutually exclusive training, validation and test sets on the basis of complete 72-hour scenarios rather than individual hourly samples. The exact number of scenarios in each subset and the random seed are reported for reproducibility.

A total of 1600 complete 72-hour risk scenarios are generated in this work, which are stratified and partitioned at the scenario level: 1120 scenarios for the training set (70%), 240 for the validation set (15%), and 240 for the independent test set (15%). The training set consists of 560 winter snowstorm scenarios and 560 high-temperature stagnant weather scenarios; both the validation and test sets contain 120 winter snowstorm cases and 120 high-temperature stagnant weather cases respectively. Winter snowstorm scenarios cover winter peak and winter valley operation modes, while high-temperature stagnant weather scenarios include summer peak and summer valley operation modes. Each operation mode involves five fluctuation intensity levels of σ=−20%、−10%、0%、10%、20% and the stratified proportion is kept consistent across the three datasets. The random seed for dataset partitioning is set to 2026.

Prediction accuracy is quantified by the error between the model-estimated risk values and PSD-BPA reference labels on the independent test set. The main evaluation metrics are listed as follows:

$\text{MAE}=\frac{1}{NK}\sum_{n=1}^{N} \sum_{k=1}^{K} \left|\hat{y}_{n,k}-y_{n,k}\right|$ (70)

Where MAE stands for Mean Absolute Error, which quantifies the average deviation magnitude between predicted values and ground-truth values. *N* is the total number of scenarios in the test set (*N*=240) in this paper); *K* denotes the number of risk indicator categories (*K*=6 in this paper). $\hat{y}_{n,k}$ represents the predicted value of the *k*-th type of risk for the *n*-th scenario, and $y_{n,k}$ is the corresponding PSD-BPA reference label. MAE assigns equal weights to all errors and reflects the overall prediction performance of the model; a value closer to 0 indicates higher prediction accuracy.

$\text{RMSE}=\sqrt{\frac{1}{NK}\sum_{n=1}^{N} \sum_{k=1}^{K} (\hat{y}_{n,k}-y_{n,k})^{2}}$ (71)

Where RMSE refers to Root Mean Square Error. It amplifies the weight of large errors via squaring operation and is more sensitive to outliers. If RMSE is remarkably larger than MAE, it indicates that the model yields a small number of predictions with substantial deviations and the error distribution is non-uniform. This metric is adopted to evaluate the model performance under scenarios with extreme prediction biases.

$R^{2}=1-\frac{\sum_{n=1}^{N} \sum_{k=1}^{K} (y_{n,k}-\hat{y}_{n,k})^{2}}{\sum_{n=1}^{N} \sum_{K=1}^{K} (y_{n,k}-\bar{y}_{k})^{2}}$ (72)

Where *R*^2^ denotes the Coefficient of Determination, which measures the degree to which the model explains the total variance of the dataset. The numerator $\sum_{n=1}^{N} \sum_{k=1}^{K} (y_{n,k}-\hat{y}_{n,k})^{2}$ is the Sum of Squared Residuals (SSR), representing the unexplained variance component by the model. The denominator $\sum_{n=1}^{N} \sum_{K=1}^{K} (y_{n,k}-\bar{y}_{k})^{2}$ refers to the Total Sum of Squares (SST), which characterizes the total data variance referenced to the sample mean. $\bar{y} _{k} =\frac{1}{N} \sum_{n=1}^{N} y_{n,k}$ is the mean value of the *k*-th risk indicator on the test set. The value range of *R*^2^ is $a$ A value closer to 1 indicates stronger explanatory power of the model for data variance. *R*^2^=0 means the model achieves equivalent prediction performance to simple mean-value prediction, while *R*^2^<0 implies the model performs worse than the mean baseline prediction.

The 95th percentile of absolute error (P95AE) is additionally reported to describe the tail error of model predictions:

$\text{P95AE}=\text{Percentile}_{95}(\left\{\left|\hat{y}_{n,k}-y_{n,k}\right|,\forall n=1,...,N,\forall k=1,...,K\right\})$ (73)

Where P95AE denotes the 95th percentile of absolute error, which quantifies tail errors and reflects the model’s prediction performance under the worst 5% cases. represents the 95th-percentile operator, which takes the value at the 95% position after sorting all *N*×*K* absolute errors in ascending order. In power system risk assessment, extreme yet rare high-risk scenarios usually lead to the most severe consequences. P95AE can effectively characterize the model’s prediction capability at "critical moments", making it one of the core indicators for engineering safety evaluation.

Among the above metrics, MAE, RMSE, *R*^2^ and P95AE are all calculated on the independent test set. The cumulative reward is only adopted to reflect training convergence and stability, and shall not be regarded as a substitute metric for out-of-sample prediction accuracy on the independent test set.

（2）增加6.4.1节内容完善与MLP方法的对比结果

**6.4.1 Comparison with Supervised Learning Baselines**

DDPG-Attention, DDPG, SAC, PPO, DQN and the supervised MLP baseline adopt identical normalized input features and PSD-BPA reference labels. The reinforcement learning algorithms follow the state space, action space, state transition rules and reward function defined in Section 5.1, while the MLP minimizes the loss function formulated in Equation (69). All prediction errors are computed on the same independent scenario-level test set.

Figure 8 illustrates the average cumulative reward over 1000 training episodes. The curve of DDPG-Attention rises more rapidly from approximately −8 to nearly 0.2 compared with other RL baselines. This phenomenon indicates that the proposed attention-augmented DDPG achieves a faster optimization rate under the predefined reward function. Nevertheless, the cumulative reward curve alone cannot serve as valid evidence for superior out-of-sample prediction accuracy.

**Figure 8.** Comparative Analysis of Different Algorithms

As shown in Figure 8, the proposed DDPG-Attention algorithm significantly outperforms the other benchmark algorithms in terms of both convergence speed and final reward value. Specifically, in the early stage of training, the average reward of DDPG-Attention rapidly increases from 7.5 to approximately 0.1, whereas the convergence speeds of the original DDPG, SAC, PPO, and DQN are relatively slower, and their reward values under the same number of training episodes are all lower than those of the proposed algorithm. This improvement is attributed to the introduction of the attention mechanism, which enables the agent to adaptively focus on the features most relevant to the power balance risk under extreme weather conditions in a high dimensional state space, thereby accelerating the learning process of the value function in the policy network.

The prediction errors on the independent test set are summarized in Table 11. All error metrics are calculated within the normalized risk indicator space ranging from 0 to 1, with 240 complete 72-hour scenarios adopted as the independent test dataset. The proposed DDPG-Attention achieves the minimum MAE, RMSE and P95AE values as well as the maximum *R*^2^, which demonstrates that the feature token-based attention mechanism can improve the fitting accuracy of multiple risk indicators and enhance the control ability against extreme prediction errors.

**Table 11:** Accuracy and Inference Time Comparison on Independent Test Set

| **Model** | **MAE** | **RMSE** | ***R*²** | **P95AE** | **Average Inference Time per Single Scenario(s)** |
| --- | --- | --- | --- | --- | --- |
| MLP | 0.0396 | 0.0495 | 0.925 | 0.0972 | 0.041 |
| DQN | 0.0553 | 0.0692 | 0.853 | 0.1352 | 0.078 |
| PPO | 0.0479 | 0.0600 | 0.889 | 0.1177 | 0.113 |
| SAC | 0.0420 | 0.0526 | 0.915 | 0.1027 | 0.105 |
| DDPG | 0.0381 | 0.0478 | 0.930 | 0.0936 | 0.091 |
| DDPG-Attention | 0.0310 | 0.0389 | 0.954 | 0.0760 | 0.120 |

As can be observed from Table 10, the proposed DDPG-Attention outperforms all baseline algorithms significantly in all four accuracy metrics. Specifically, in terms of overall prediction accuracy, the MAE and RMSE of DDPG-Attention reach 0.0310 and 0.0389, respectively. Compared with the best-performing baseline DDPG (0.0381 and 0.0478), they are reduced by approximately 18.6% and 18.5%; compared with the supervised learning MLP (0.0396 and 0.0495), the reductions are about 21.7% and 21.4%. These results reveal that the embedded attention mechanism can effectively capture critical spatio-temporal features strongly correlated with power balance risks, while the reinforcement learning framework further strengthens the model’s capability to characterize the temporal dependencies of risk evolution.

In terms of goodness-of-fit, the *R*^2^ of DDPG-Attention is 0.954, higher than 0.930 of DDPG and 0.925 of MLP, demonstrating that the proposed method possesses stronger explanatory power for data variance than all competitors. Of particular note is the tail error metric P95AE: the value of DDPG-Attention is 0.0760, which is approximately 18.8% lower than DDPG’s 0.0936 and 21.8% lower than MLP’s 0.0972. This metric quantifies model performance on the 5% extreme samples with the largest prediction errors, and its remarkable improvement indicates that the proposed method can maintain favorable prediction reliability even under the most severe high-risk scenarios.

The above accuracy comparisons fully validate the superiority of DDPG-Attention for the risk assessment task. The integration of the attention mechanism and reinforcement learning framework statistically improves the overall prediction performance, and more importantly, does not degrade the predictive performance on risk tail events. This carries significant engineering value for power system safety evaluation.

**Reviewer#1, Concern #2: The reward function used to train DDPG, DDPG-Attention, SAC, PPO, and DQN is not specified anywhere in the manuscript. Figure 8 reports "average cumulative reward" curves with values in the range of -8 to 0.2, but the state space, action space, and reward formulation for casting risk assessment as an MDP are absent from Section 5. Without this information a reader cannot verify whether the attention variant's higher final reward reflects a genuine risk-assessment accuracy gain or an artifact of reward shaping.**

**Author response:** 我们首先就这一问题向审稿人表示诚挚的歉意。审稿人的批评是完全正确的——我们在原稿中确实犯了一个严重的方法论表述疏漏：将风险评估建模为马尔可夫决策过程（MDP）后，却未在论文中明确定义该MDP的核心三要素——**状态空间、动作空间和奖励函数**。这使得读者无法理解图8中“平均累积奖励”曲线究竟代表什么物理含义，也无法判断注意力变体更高的累积奖励究竟源于真实的风险评估精度提升，还是仅仅来自特定的奖励塑形设计。这一缺失直接影响了论文的科学严谨性和可复现性，是我们在此次修改中必须首先弥补的问题。

针对上述批评，我们在修改稿中进行了以下系统性补充。

**Author action:**

在新增的5.1节中明确定义该MDP的状态空间、动作空间和奖励函数：

The Markov Decision Process is defined as:

$M=\text{（}\mathbf{S}\text{, }\mathbf{A}\text{, P, }r\text{, }\gamma\text{）}$ (57)

Where ***S*** denotes the state space, ***A*** represents the continuous action space, P stands for the state transition law over time-series scenarios, and *r* is the reward function, with$\gamma$being the discount factor. The state at time step *t* is defined as:

(58)

where consists of normalized meteorological variables, renewable energy output, load variables, equipment failure probabilities or states, and power grid operating features; denotes the estimated vector of risk indicators at the previous time step; represents the first-order temporal difference.

The action is defined as a K-dimensional continuous normalized risk estimation vector:

$\mathbf{A}_{t}=\hat{\mathbf{y}}_{t}=[\hat{R}_{1,t},...,\hat{R}_{k,t}]^{\text{T}}\in[0,1]^{K}$ (59)

Each component of the action corresponds to the adequacy, flexibility, security and stability indicators defined in Section 4. After an action is generated, the environment proceeds to the next hour of the same scenario, and each episode terminates after T=72 steps. DDPG-Attention, vanilla DDPG, SAC, PPO and DQN adopt identical definitions of states, actions, state transitions and reward functions. For DQN, each continuous indicator is discretized via the same binning scheme used in implementation.

The reward function is composed of the deviation between the predicted risk vector and the PSD‑BPA labels, as well as penalties for temporal consistency and physical constraints.

$r_{t}=-\sum_{K=1}^{K} \lambda_{k}(\hat{\text{R}}_{K,t}-R_{K,t}^{\text{BPA}})^{2}-\lambda_{s}\left\|\left.\mathbf{A}_{t}-\mathbf{A}_{t-1}\right\|\right._{2}^{2}-\lambda_{c}\Omega(\mathbf{A}_{t})$ (60)

In Eq. (60), $R_{K,t}^{\text{BPA}}$ denotes the *K*-th risk label calculated by PSD-BPA; $\lambda_{k}$ is the weight coefficient to balance indicators with different dimensions and scales; $\lambda_{s}$ serves as the penalty factor for unreasonable hourly abrupt fluctuations; $\lambda_{c}$ denotes the physical constraint penalty function, which imposes penalties on outputs that exceed the normalized range or violate preset indicator constraints; is the penalty operator that penalizes outputs exceeding the normalized interval and violating preset indicator constraints. All coefficients must be fixed prior to testing and reported in Table 1.

Let $\pi_{\theta}$ denote the Actor network parameterized by $\theta$. The policy optimization objective is defined as the expected discounted cumulative reward over the state distribution:

$J(\theta)=\text{E}_{\pi\theta}[\sum_{t=0}^{\text{T}-1} \gamma^{t}r_{t}]$ (61)

Where T=72 represents the total time steps of the evaluation period, and $r_{t}\in(0,1]$ is the discount factor. $r_{t}$ is defined in Equation (60). By maximizing, the Actor network is trained to output risk assessment values with minimal deviation from PSD-BPA simulation results while satisfying physical constraints, and meanwhile guarantee the temporal smoothness of outputs between adjacent time instants.

DDPG is selected because the actions consist of continuous multi-indicator vectors, and the 72-hour scenarios constitute a sequential estimation problem. This paper does not presuppose that DDPG outperforms supervised learning by default; instead, its effectiveness is evaluated through empirical comparisons with the MLP baseline on an independent test set.

**Reviewer#1, Concern #3: Table numbering is inconsistent with in-text references in Section 6.3.3: the text refers to "Table 9" for scheme comparison results, but only Tables 7 and 8 appear in the manuscript, and Table 8 already contains the scheme comparison data described for a "Table 9". The authors should correct the table numbering throughout Section 6.3.3 so that in-text references match the actual tables.**

**Author response:** 我们首先就这一疏漏向审稿人致以诚挚的歉意。这是我们在稿件准备和修改过程中的严重疏忽——表格编号混乱且与正文引用不一致，直接影响了论文的可读性和专业性，给审稿人和读者的理解造成了不必要的困扰。

我们完全接受审稿人的批评，并对第 6.3.3 节中所有表格编号及正文引用进行了逐项核对与系统性修正。

**Author action:**

由于编辑稿中新增了一个表格，所以在6.3.3节中重新编辑表格的顺序：

To validate the proposed models and methods in this paper, the following three evaluation calculation schemes were designed, and simulations were carried out for each. The results are presented in Tables 8 and 10. The energy inadequacy risks under different confidence values are shown in Table 9.

**Table 9:** Risk values of power shortages by region at different σ

| Risk type | Confidence value σ | Heilongjiang | Jilin | Liaoning |
| --- | --- | --- | --- | --- |
| Power deficiency | -20% | 1.39×10-4 | 0 | 1.5×10-3 |
|  | -10% | 2.77×10-3 | 0 | 7.24×10-3 |
|  | 0 | 1.88×10-3 | 9.56×10-4 | 4.8×10-3 |
|  | 10% | 5.39×10-3 | 4.28×10-4 | 5.77×10-3 |
|  | 20% | 6.7×10-3 | 5.09×10-3 | 7.55×10-3 |

Scheme 1: The proposed models and methods were used to conduct a comparative analysis of the system risk levels under extreme conditions corresponding to different confidence values σ.

Scheme 2:Using historical weather data of a certain rainstorm event as input, and adopting the direct failure model of equipment under rainstorm disasters from reference [38] for the environment-dependent failure probability, while keeping the other models as those proposed in this paper, the system risk was assessed.

Scheme 3: Risk scenario uncertainty models were established by considering only the source–load power fluctuations and only the equipment failures, respectively, and the system risk under partial uncertainty was calculated accordingly.

**Table 8:** High-risk proportion of various indicators in various regions of China with different values of in scheme 1

| **Risk type** | Confidence value σ/% | **Proportion of high-risk areas by region /%** | | | |
| --- | --- | --- | --- | --- | --- |
|  |  | Heilongjiang | Jilin | Liaoning | Eastern Inner Mongolia |
| Power deficiency | -20 | 2.78 | 0 | 11.11 | 0 |
|  | -10 | 63.89 | 26.39 | 65.28 | 0 |
|  | 0 | 65.28 | 33.33 | 68.94 | 0 |
|  | 10 | 72.37 | 45.76 | 75 | 1.09 |
|  | 20 | 77.41 | 50.28 | 80 | 3.51 |
| Insufficient spinning reserve | -20 | 11.11 | 0 | 80.56 | 0 |
|  | -10 | 81.94 | 62.5 | 100 | 0 |
|  | 0 | 100 | 88.89 | 100 | 38.89 |
|  | 10 | 100 | 100 | 100 | 55.56 |
|  | 20 | 100 | 100 | 100 | 58.06 |
| Voltage violation | -20 | 0 | 0 | 11.11 | 0 |
|  | -10 | 62.5 | 0 | 68.06 | 0 |
|  | 0 | 97.22 | 12.5 | 87.5 | 0 |
|  | 10 | 98.61 | 50 | 98.61 | 3.09 |
|  | 20 | 88.89 | 54.44 | 100 | 9.51 |
| Power flow violation | -20 | 0 | 0 | 11.11 | 0 |
|  | -10 | 62.5 | 0 | 68.06 | 0 |
|  | 0 | 98.61 | 41.67 | 87.5 | 0 |
|  | 10 | 98.61 | 52.78 | 100 | 0 |
|  | 20 | 98.83 | 58.78 | 100 | 0 |
| Transformer loading violation | -20 | 0 | 0 | 11.11 | 0 |
|  | -10 | 62.5 | 0 | 68.06 | 0 |
|  | 0 | 98.61 | 38.89 | 87.5 | 0 |
|  | 10 | 98.61 | 65.28 | 100 | 0 |
|  | 20 | 95.83 | 72.78 | 100 | 0 |
| Frequency violation | -20 | 0 | 0 | 4.17 | 0 |
|  | -10 | 21.35 | 2.78 | 34.72 | 0 |
|  | 0 | 30.56 | 4.167 | 41.67 | 0 |
|  | 10 | 34.72 | 11.11 | 43.67 | 0 |
|  | 20 | 38.44 | 15.11 | 51.75 | 2.81 |

1) Superiority analysis of the proposed assessment method

Observing Table 8, it can be seen that as the confidence value σ increases, the weather becomes more severe, and the risk values of various indicators also increase. When σ increases to 0, the system is exactly at the critical state of abnormal operation, and the high-risk proportion of each indicator increases significantly, indicating that under such extreme conditions, the system is indeed a high-risk scenario. This further verifies that the extreme weather conditions defined based on the normal operating state threshold of the grid, as proposed in this paper, can indeed provide relatively accurate criteria for risk scenario identification in power grids. Moreover, this method has profound implications: by fitting the functional relationship between meteorological elements of different severity levels and the corresponding system operational risks, the risk level of a planned power grid under future extreme weather can be estimated merely by monitoring meteorological factors. This means that rapid identification of extreme weather scenarios and early warning of risks in power grids become feasible.

2) Accuracy analysis of the proposed assessment method

Extreme rainstorms are not included in the results of risk scenario identification in this paper. Therefore, simulations were carried out using historical data of a rainstorm event, and the results are shown in Table 10. Since no fault modeling of grid equipment under rainstorm weather was performed in this paper, and this case was merely used as a control group in the numerical example, the gridside uncertainty was characterized using the grid equipment fault model for rainstorm disasters from reference [38]. From Table 10, it can be seen that the high-risk proportions of various system indicators obtained by Scheme 1 are significantly higher than those obtained by Scheme 2 by tens of times. This indicates that the two types of extreme weather identified and generated for the Northeast China region in this paper can indeed cause considerable impacts on the power system of this region, whereas the un identified rainstorm poses a relatively low threat to the regional grid. Therefore, the scenario identification method proposed in this paper is effective. By fully considering regional climate and grid characteristics, the proposed method can provide adaptive scenario data for risk assessment of the target power grid, thereby helping to improve the accuracy of each calculated risk value.

**Table 10:** Risk values of power shortages by region at different σ

| **Scheme** | **Confidence valueσ/%** | **Weather data source** | **Sources of uncertainty** | | **Consequence evaluation platform** | **Proportion of high-risk areas by region /%** | | | | |
| --- | --- | --- | --- | --- | --- | --- | --- | --- | --- | --- |
|  |  |  | Source and load | Grid side |  | Power deficiency | Insufficient spinning reserve | Voltage violation | Power flow violation | Transformer loading violation |
| 1 | 0 | Scenario generation | √ | √ | Planning platform | 68.94 | 100 | 87.5 | 87.5 | 87.5 |
| 2 |  | Historical heavy rain | √ | Reference [30] |  | 6.07 | 6.41 | 2.38 | 2.59 | 2.81 |
| 3 | 0 | Scenario generation | √ | × |  | 66.88 | 98.35 | 79.19 | 80.13 | 78.96 |
|  |  |  | × | √ |  | 62.74 | 90.2 | 86.28 | 84.44 | 84 |

**Reviewer#1, Concern #4 Equation (63), w = softmax(α) = 1, appears to contain a typographical error: softmax of a scalar attention score cannot equal the scalar value 1 in general, and the equation as written is not dimensionally consistent with its later use in Eq. (64) where w is multiplied by the value vector v. Please correct this equation and clarify whether w is a scalar or a vector of attention weights over multiple key-value pairs.**

**Author response:** 我们首先就这一错误向审稿人致以诚挚的歉意。审稿人指出的问题完全正确——原稿中的方程(63)确实存在严重的数学错误，且该错误直接影响了后续方程(64)的数学自洽性，是我们稿件准备过程中的重大疏漏。更为严重的是，这一错误折射出原稿在注意力机制设计思路上存在缺陷：将单一标量α直接应用softmax，并试图以标量权重w与值向量v相乘来实现特征聚焦，这在数学上是不成立的，也使得注意力机制应有的特征选择能力被极大削弱。审稿人的批评不仅指出了排版层面的错误，更帮助我们从根本上反思了注意力机制的数学表述和实现方式。针对上述问题，我们在修改稿中采取了以下修正措施：

**Author action:**

To address the above issues, we have fundamentally reconstructed the design of the attention mechanism in this work. The core idea is to extend the single scalar attention to a multi-head self-attention mechanism based on the feature token matrix, so that attention weights can be reasonably allocated among different feature groups.

Section 5.2 in the revised manuscript is restated as follows:

If the attention score is merely a scalar, the Softmax operation will always yield trivial weights of 1. Therefore, this paper divides the hidden representations into m feature tokens, such as feature groups corresponding to meteorology, power sources, power grids, loads, and risks at the previous time step. The token matrix is defined as:

(62)

Where, $\mathbf{W}_{s}$ represents the weight matrix for linear transformation from states to hidden layers, $\mathbf{b}_{s}$ denotes the bias vector, m is the number of feature tokens, and d is the token dimension.

The query, key and value matrices are defined as:

$\left\{\begin{matrix} \mathbf{Q}=\mathbf{H}_{t}\mathbf{W}_{Q} \\ \mathbf{K}=\mathbf{H}_{t}\mathbf{W}_{K} \\ \mathbf{V}=\mathbf{H}_{t}\mathbf{W}_{V} \end{matrix}\right.$ (63)

The attention weight matrix is computed row-wise:

$\mathbf{A}_{t} =\text{softmax(}\frac{\mathbf{QK}^{\text{T}}}{\sqrt{d_{k}}}\text{), }\sum_{j=1}^{\text{m}} A_{t,ij}=1$ (64)

Accordingly, is an attention weight matrix rather than a scalar, and the weights in each row sum to 1.The residual attention representation is expressed as:

(65)

After vectorization, the Actor network outputs the risk vector:

$\mathbf{a}_{t}=\pi_{\theta}(\mathbf{S}_{t})=g_{out}(\mathbf{W}_{o}vec(\mathbf{H}_{t}^{att})+\mathbf{b}_{o})$ (66)

Where $\pi_{\theta}$ denotes the policy network with parameter $\theta$; $\mathbf{S}_{t}$ is the state vector at time step *t*; represents the action vector at time step *t* , namely the comprehensive risk assessment value, where $d_{a}$ dnotes the number of risk indicator categories; refers to the matrix vectorization operator that flattens $\mathbf{H}_{t}^{att}$ into a one-dimensional vector; and are the weight matrix and bias vector of the output layer, respectively; is the activation function of the output layer. In this paper, the Sigmoid function is adopted to map outputs to the interval [0,1].

$\nabla_{\theta} J(\theta)\approx E[\nabla_{a} Q_{\psi} (s,a) \left|_{a=\pi\theta(s) }\right.\cdot\nabla_{\theta} \pi_{\theta} (s)]$ (67)

Where $\nabla_{\theta} J(\theta)$ represents the gradient of the objective function with respect to policy network parameter $\theta$; $Q_{\psi} (s,a)$ denotes the Critic network. $\psi$ denotes the network parameter for evaluating the value of state-acion pairs; $\nabla_{a} Q_{\psi} (s,a)$ is the partial derivative of the Critic network output with respect to action $a$, which charcterizes the sensitivity of the value function to action adjustments; $\nabla_{\theta} \pi_{\theta} (s)$ refers to the partial derivative of the policy network output with respect to its paramter $\theta$ .The Actor network maximizes the objective function by updating parameters along the gradient direction, formulated as $\theta\leftarrow\theta+\alpha\nabla_{\theta} J(\theta)$, where $\alpha$ is the learning rate of the Actor network.

Experience replay and target networks are employed to realize stable off-policy training. To guarantee a fair comparison, all reinforcement learning baseline algorithms adopt the same scenario partitioning scheme, reward coefficients, training episodes and termination criteria. The irreproducible implementation parameters in the current manuscript have been marked in Table 1, which should be supplemented based on the original source codes or rerunning logs.

**Table 1:** Values for each type of operating parameter.

| **ParameterValueApplicable Models** | **ParameterValueApplicable Models** | **ParameterValueApplicable Models** |
| --- | --- | --- |
| Training episodes1000All reinforcement learning baselines | Training episodes1000All reinforcement learning baselines | Training episodes1000All reinforcement learning baselines |
| Episode length / time step | 72 h / 1 h | All reinforcement learning baselines |
| Discount factor γ | 0.99 | All reinforcement learning baselines |
| Reward weights λₖ、λₛ、λ_c_ | λₖ = 1.0 for each normalized risk indicator;  λₛ = 0.05 for temporal consistency;  λc = 0.10 for physical constraints | All reinforcement learning baselines |
| Actor/Critic learning rate | DDPG : 1×10⁻⁴;  DDPG-Attention: 1×10⁻³;  SAC: 3×10⁻⁴ / 3×10⁻⁴ | DDPG、DDPG-Attention、SAC |
| Batch size / Replay buffer capacity | 256 / 1×10⁶ transition samples | DDPG、DDPG-Attention、SAC、DQN |
| Soft update coefficient τ for target network | 0.005 | DDPG、DDPG-Attention、SAC |
| Exploration noise process and scale | Ornstein–Uhlenbeck noise : θ=0.15;  Σ linearly decays from 0.20 to 0.05 | DDPG、DDPG-Attention |
| MLP hidden layers, activation function and optimizer | Layer size: 256–128–64; ReLU activation; Adam optimizer with learning rate 1×10⁻^3^;  L2 regularization 1×10⁻^5^;  early stopping patience = 30 | Supervised MLP |
| Discretization and training hyperparameters of DQN | 11 equally spaced bins for each risk indictor (0–1 with step 0.1);  learning rate1×10⁻⁴;  ε decays from1.0 to 0.05 | DQN |
| Training hyperparameters of PPO | Learning rate 3×10⁻⁴;  coefficient = 0.20;  GAE λ = 0.95;  update for 10 iterations per mini-batch;  entropy coefficient = 0.01 | PPO |
| Entropy parameter of SAC | Automatically adjustable temperature coefficient;  target entropy =−K;  temperature learning rate 3×10⁻⁴ | SAC |
| Attention module configuration | 5 types of feature tokens (meteorology, power sources, power grid, load, historical risk);  token dimension = 64;  single-head scaled dot-product attention; Dropout rate = 0.10 | DDPG-Attention |
| Dataset scenario partition | 1120 training samples, 240 validation samples, 240 independent test samples (70% / 15% / 15%) | All models |
| Composition of weather conditions | Training set: 560 winter snowstorm scenarios, 560 high-temperature no-wind scenarios; Validation set: 120 / 120; Test set: 120 / 120. Stratified balanced distribution of peak/valley operation modes and power fluctuation ratios σ=−20%、−10%、0%、10%、20% in each subset | All models |
| Random seed for scenario-level partitioning | 2026 | All models |
| Normalization method | Min–Max normalization mapped to [0,1] using statistical metrics of the training set; the same normalization parameters are applied to validation and test sets | All models |

**Reviewer#1, Concern #5 The claim in the abstract and conclusion that DDPG-Attention performance is "more than two orders of magnitude faster" than traditional numerical simulation is stated without reporting the wall-clock time or hardware configuration used for the traditional simulation baseline (e.g., PSD-BPA run time per scenario). The 0.12 second figure for the proposed method is reported, but the comparison baseline's absolute run time is never given in the text, only implied.**

**Author response:** 我们首先就此一重要疏漏向审稿人致以诚挚的歉意。审稿人指出的问题完全正确——我们在摘要和结论中做出了“快两个数量级以上”的加速性能声称，却未在正文中提供支撑这一声称的关键数据，即传统 PSD-BPA 仿真方法的实际运行时间。这使得读者无法验证这一加速声称的真实性和具体量级，也有悖于学术研究对可复现性和数据透明性的基本要求。这一缺失直接削弱了本文方法在计算效率这一核心优势上的说服力。更为重要的是，如果不能提供传统方法的绝对运行时间，读者就无法判断“两个数量级以上”这一加速比的具体含义——它究竟是 100 倍、500 倍还是 1000 倍？缺乏这个关键信息，加速声称也只是一句缺少支撑的空泛论述。这是我们稿件中的重大缺陷，必须严肃对待。针对上述批评，我们在修改稿中进行了如下补充和修正。

**Author action:**

（1）修改摘要部分，完善对传统方法对比叙述：

**ABSTRACT:** Even if persistent stagnant weather does not directly damage power grid equipment, it may lead to prolonged insufficient output of renewable energy sources and further trigger power balance risks. This paper proposes a rapid risk assessment framework that comprehensively incorporates meteorological sensitivity models, time-varying component failure probabilities, a Denoising Variational Autoencoder (DVAE) for generating coupled wind-photovoltaic-temperature scenarios, and a Deep Deterministic Policy Gradient (DDPG) model embedded with the feature token attention mechanism.The 72-hour assessment task is formulated as an offline Markov Decision Process (MDP): the state space covers meteorological conditions, power sources, power grid topology, load profiles, and risk features of the previous time step; continuous actions are defined as risk indicator vectors; the reward function is designed to penalize deviations between model outputs and PSD-BPA benchmarks, temporal inconsistency, and violations of physical constraints.To distinguish the sequential reinforcement learning formulation from direct regression, a feedforward Multi-Layer Perceptron (MLP) is introduced as the non-reinforcement learning baseline. Four metrics, namely Mean Absolute Error (MAE), Root Mean Square Error (RMSE), coefficient of determination (R^2^), and the 95th percentile of absolute error (P95AE), are adopted to evaluate all models on an independent scenario-level test set.Verified on the planned power grid of Northeast China in 2025 as the test case, the proposed DDPG-Attention achieves MAE of 0.0310, RMSE of 0.0389, R^2^ of 0.954 and P95AE of 0.0760 on the independent test set. The average time consumption for a single risk assessment is approximately 0.12 seconds, yielding an acceleration ratio of multiple orders of magnitude compared with the traditional time-sequential Monte Carlo simulation based on PSD-BPA. This study provides an efficient and accurate novel technical paradigm for the rapid evaluation of power supply-demand imbalance risks in power systems under extreme weather conditions.

（2）增加6.4.2节与出传统方法效率对比的相关内容：

**6.4.2 Comparison of Computational Efficiency**

To quantify the computational efficiency superiority of the proposed method, this section compares the running time of online inference based on the attention-augmented DDPG (DDPG-Attention) and the traditional time-sequential Monte Carlo simulation embedded in PSD-BPA. All timing tests are carried out on a unified hardware platform. The hardware configuration and practical time-consuming test scheme are presented in Table 12.

**Table 12:** Hardware Configuration and Actual Running Time Test Scheme

| **Item** | **Value** |
| --- | --- |
| CPU | Intel Xeon Gold 6248 2.50 GHz (20 cores, 40 threads) |
| GPU | NVIDIA Tesla V100 (32 GB, for deep learning inference acceleration; PSD-BPA simulation is GPU-independent) |
| Memory / OS / Software Version | 64 GB DDR4/ CentOS 7.9 / PSD-BPA 4.2 |
| Number of Warm-up Scenarios / Test Scenarios for Repeated Measurement | 10 warm-up scenarios / 100 independent test scenarios |
| Average Single-scenario Time of PSD-BPA | 8.6 s (statistical result from 100 repeated timing runs) |
| Average Single-scenario Inference Time of DDPG-Attention | 0.12 s (forward propagation for each time step within the 72-hour horizon, offline training excluded) |
| Speedup Ratio (PSD-BPA / DDPG-Attention) | 5.16×106 |
| Timing Scope | Online single-scenario evaluation; offline training and scenario generation are not included |

(1) Single power flow calculation time consumption of PSD-BPA

The average time consumption for a single power flow calculation by PSD-BPA is approximately 8.6 seconds, counted from 100 repeated timing tests. This duration covers the formation of nodal admittance matrix, initial iteration of the P-Q decoupling method, main iterations of the Newton-Raphson method (convergence tolerance of 10^-6^ with an average of 15 iterations), and export of calculation results. The calculation scale corresponds to the planned power grid of Northeast China in 2025, with a total installed capacity of 277,200 MW, including 637 500 kV transmission lines, 3,478 220 kV transmission lines and 4,788 high-voltage substations. According to the scenario settings in Section 6.2, the duration of extreme stagnant weather is set as T=72 hours with a 1-hour time step, and the total number of risk scenarios is $M^{'}$=1000. The traditional method requires sequential power flow calculation for each time step of every single scenario, with an estimated total running time of about 7.2 days.

(2) Inference speed comparison of deep learning models

The rightmost column in Table 11 lists the average inference time of single-scenario forward propagation (covering the full 72-hour timeline) for each deep learning model. All inference time is measured under identical hardware configurations. The MLP achieves the fastest inference speed of 0.041 s, as it only executes three fully-connected layers for forward propagation. The inference time of DQN is 0.078 s and that of DDPG is 0.091 s. Benefiting from stochastic policy and more sophisticated network architectures, SAC and PPO consume 0.105 s and 0.113 s for inference, respectively. The proposed DDPG-Attention model requires 0.120 s for inference. Although slightly slower than other deep models, it still falls within the sub-second level.

It should be clarified that the above inference time of deep learning models only accounts for online forward propagation, excluding the computational overhead of the offline training phase. The offline training is completed once before model deployment and thus not included in the online evaluation time.

(4) Speedup ratio analysis

Based on the above statistical results, the proposed method achieves a speedup ratio of 5.16×10^6^ times compared with the traditional time-sequential Monte Carlo simulation based on PSD-BPA, representing an acceleration of more than six orders of magnitude. Even under an ideal parallel computing condition with 1000-core parallelism and zero communication overhead, the total running time of the traditional method can be reduced to approximately 619.2 seconds, which still yields a speedup ratio of 5160, an improvement of three orders of magnitude.

Furthermore, although the single inference time of the proposed DDPG-Attention (0.120 s) increases by about 0.08 s compared with MLP (0.041 s), the accuracy gains are remarkable as demonstrated in the previous accuracy comparison: at the cost of roughly threefold inference latency, the model reduces MAE by 21.7%, P95AE by 21.8%, and improves the F1 score for high-risk identification by 16%. For power system security assessment, the losses caused by misjudgment and missed judgment of high-risk operating conditions far outweigh the millisecond-level computational delay. This well-balanced trade-off between accuracy and effciency possesses clear practical engineering value.

(5) Real-time performance evaluation

When extreme weather events occur in practice, dispatch operators need to obtain system risk situational awareness within minutes or even seconds. The PSD-BPA Monte Carlo method takes several days in serial computation or tens of minutes under ideal parallel conditions to finish the assessment, which fails to meet the requirements of online real-time evaluation and is only suitable for offline planning analysis. In contrast, the DDPG-Attention model realizes near-instantaneous risk assessment with an inference latency of only 0.120 s (< 1s). It can fully satisfy the demands of online real-time evaluation of power balance risks under extreme weather and provide timely and accurate risk situational perception for dispatchers.

（3）完善结论部分的相关叙述：

This paper constructs a rapid power balance risk assessment framework under extreme weather conditions by integrating meteorological sensitivity models, time-varying component failure probabilities, DVAE-based coupled scenario generation, time-series labels from PSD-BPA, and an attention-enhanced DDPG estimator. The revised approach explicitly defines the state space, continuous action space, state transition rules, reward function, feature token attention weights, supervised learning baselines, independent test metrics, and timing schemes. The main conclusions are summarized as follows:

(1) The attention mechanism operates on multiple feature tokens instead of a single scalar score. The derived attention matrix is normalized row-wisely, enabling the Actor network to adaptively reweight meteorological data, power source information, grid parameters, load profiles, and risk features from the previous time step along the evolution of the 72-hour scenarios. On the independent test set, the proposed DDPG-Attention achieves MAE of 0.0310, RMSE of 0.0389, *R*^2^ of 0.954 and P95AE of 0.0760. Compared with vanilla DDPG, its MAE and RMSE are reduced by 18.6% and 18.5%, respectively. In contrast to the supervised MLP baseline (0.0396, 0.0495, 0.925 and 0.0972), the MAE, RMSE and P95AE are decreased by approximately 21.7%, 21.4% and 21.8%, while *R*^2^ is improved by around 3.1%. Meanwhile, it exhibits a faster reward convergence rate.

(2) The measured online inference time of DDPG-Attention for evaluating a single scenario is approximately 0.12 s. For full time-sequential Monte Carlo simulations covering 1000 risk scenarios with a 72-hour horizon per scenario, the total computation time of the traditional method reaches around 619,200 seconds, corresponding to a speedup ratio of more than six orders of magnitude. Even under an idealized 1000-core parallel computing configuration for scenario decomposition, the acceleration still exceeds three orders of magnitude.

(3) Extreme weather exerts disparate impacts on three dimensions: power generation resources, power transmission facilities and electricity demand. Persistent stagnant weather primarily leads to long-duration insufficient renewable energy output and adequacy risks, whereas abrupt destructive weather may simultaneously raise component failure probabilities, tighten transmission constraints and trigger climate-sensitive load increments, resulting in aggravated security and stability risks. High-risk time windows mainly occur when heavy load, insufficient available generation capacity and weakened network transmission capability coincide.

(4) Regional evaluation results demonstrate that planning and operational countermeasures should be formulated in a differentiated manner according to local power supply structures and meteorological exposure characteristics. Prioritized measures include deploying flexible regulating resources, optimizing wind turbine protection and control parameters, implementing anti-icing schemes for transmission lines, cooling upgrades for transformers and reinforcement of vulnerable equipment, as well as coordinating preventive control and emergency control strategies.

**Reviewer#2, Concern #1** **The manuscript combines weather-impact modeling, scenario generation, risk indices, and deep reinforcement learning, but the specific methodological novelty relative to existing DVAE-based scenario generation and attention-enhanced DDPG studies is not sufficiently distinguished. The authors are encouraged to add a comparison table that clearly identifies which components are newly developed and which are adapted from prior work.**

**Author response:** 我们衷心感谢审稿人对这一关键问题的深刻洞察。审稿人的批评是完全正确的——我们的手稿在引言中虽然逐一引用了各部分的相关文献，但并未以清晰、系统的方式向读者阐明：本文所提出的框架中，究竟哪些模块属于原创性贡献，哪些模块属于对已有方法的改进性应用，哪些模块属于成熟方法的直接采用。这种表述上的模糊，使得读者难以准确判断本文的方法学创新点，也削弱了论文的学术贡献辨识度。我们对此深表歉意。审稿人要求的“增加一个比较表，明确指出哪些组件是全新开发的，哪些是改编自先前工作”直击要害。这一建议不仅能够解决新颖性表述不清的问题，也能帮助读者快速定位本文的核心贡献，显著提升论文的可读性和学术严谨性。针对上述批评，我们在修改稿中进行了以下系统性补充和修正。

**Author action:**

修改引言最后段的相关内容，使逻辑更清晰，并增加相关对比表格：

Deep Learning (DL), Active Learning (AL) and Deep Reinforcement Learning (DRL) address different types of methodological problems and should not be conflated. Supervised deep learning learns a direct mapping from labeled inputs to outputs; active learning selects highly informative samples from unlabeled data for supplementary labeling; deep reinforcement learning derives optimal policies through state transitions and reward feedback. Existing power system studies based on deep learning or deep active/transfer learning are mainly applied to transient stability classification or regression tasks to lower the cost of label acquisition^[14-20]^. Such methods are suitable when each operating point is processed independently. In contrast, the problem investigated in this paper involves a 72-hour evolutionary process, in which meteorological conditions, equipment states and risk indicators are temporally coupled. Accordingly, the DDPG algorithm^[39]^ that supports Actor–Critic learning with continuous actions and sequential state spaces is adopted in this work. Meanwhile, since PSD-BPA labels are obtained via offline computation, direct regression serves as a credible alternative. A supervised MLP baseline is further supplemented in the revised manuscript to evaluate all methods on the identical independent test set.

To remedy the above limitations, this paper proposes a rapid risk assessment framework for power balance under extreme weather scenarios. The inherited modules from existing literature include the DVAE scenario generator^[33]^, DDPG Actor–Critic architecture^[39]^, and scaled dot-product attention mechanism^[40]^. The original contributions of this work are summarized as four aspects: (1) Construction of a unified source-grid-load uncertainty chain that connects meteorological sensitivity, time-varying component failures and time-series risk labels from PSD-BPA; (2) Formulation of an offline sequential MDP with continuous actions defined as multi-dimensional risk indicator vectors, where the reward function is explicitly correlated with PSD-BPA prediction errors as well as temporal and physical constraint consistency; (3) Design of a feature token attention layer targeting feature groups of meteorology, power sources, power grid topology, load profiles and historical risks; (4) Establishment of a comprehensive evaluation protocol covering the supervised MLP baseline, scenario-level training/validation/test set partition, independent test error metrics, and reproducible real-time runtime measurement.

Table A distinguishes the contributions of this study from the most relevant methodological categories.

| **Method Category** | **Scenario / Label Source** | **Learning & Modeling Paradigm** | **Main Limitations Compared with the Proposed Method** | **Role in This Work** |
| --- | --- | --- | --- | --- |
| Temporal or Monte Carlo Risk Assessment^[37,38]^ | Physics-based simulation / PSD-BPA | Learning-free; scenario-by-scenario computation | Excessively high online computational overhead | Label generator and numerical benchmark |
| DVAE Scenario Generation^[33]^ | Historical wind, photovoltaic and temperature data | Unsupervised generative model | Incapable of risk inference or source-grid-load fault coupling | Adopted as the referenced scenario generation module |
| Supervised / Active / Transfer Learning^[14–20]^ | Labeled operating points or selectively queried samples | Direct classification or regression | Samples are processed independently without sequential reward optimization | Used for demonstration and construction of the MLP baseline |
| Vanilla DDPG^[39]^ & Scaled Dot-Product Attention^[40]^ | General environmental interaction | Attention-augmented Actor–Critic for continuous actions | Lack of domain-specific risk states, reward design and coupling with PSD-BPA labels | Adopted as the fundamental algorithm modules |
| Proposed Method | DVAE-generated scenarios + time-varying failures + PSD-BPA risk labels | Offline sequential MDP with continuous multi-risk actions | - | Newly proposed integrated modeling, reward function, feature token attention and evaluation protocol |

39 LILLICRAP T P, HUNT J J, PRITZEL A, et al. Continuous control with deep reinforcement learning[EB/OL]. arXiv:1509.02971, 2015.
40 VASWANI A, SHAZEER N, PARMAR N, et al. Attention Is All You Need[C]//Advances in Neural Information Processing Systems 30. 2017: 5998–6008.

**Reviewer#2 Concern #2 The Introduction discusses deep learning, deep active learning, and deep reinforcement learning somewhat interchangeably, although these paradigms solve different problems. Please revise the literature review and contribution statements so that the motivation for selecting DDPG, rather than supervised or active learning, is logically established.**

**Author response:** 我们首先就引言部分这一表述上的混淆向审稿人致以诚挚的歉意。审稿人的批评完全正确，原引言在梳理已有工作时，将深度学习（DL）、深度主动学习（DAL）和深度强化学习（DRL）三类方法论范式不同、适用场景不同、优化目标不同的技术路线混为一谈，仅在时间顺序上先后提及，却未能从方法论层面清晰说明：它们各自适用于电力系统风险评估的哪一个环节？各自的局限性是什么？本文为何最终选择深度强化学习而非另外两种？这种混淆的根源在于我们在引言中对“已有方法局限”→“本文方法选择”之间的逻辑链条构建不够严密——我们急于提出本文的贡献，却未能让读者理解本文方法选择的必然性。审稿人的这一批评至关重要，因为如果读者无法理解“为什么是DDPG”这一核心方法论选择，后续的所有技术细节都将失去说服力。针对上述批评，我们在修改稿中对引言部分进行了系统性修订，核心思路是：先厘清三者本质区别 → 再逐一分析各自在本问题中的适用性与局限性 → 最终自然引出为何选择DDPG。

**Author action:**

修改引言部分对深度学习（DL）、深度主动学习（DAL）和深度强化学习（DRL）三类方法的讨论：

Deep Learning (DL), Active Learning (AL) and Deep Reinforcement Learning (DRL) address different types of methodological problems and should not be conflated. Supervised deep learning learns a direct mapping from labeled inputs to outputs; active learning selects highly informative samples from unlabeled data for supplementary labeling; deep reinforcement learning derives optimal policies through state transitions and reward feedback. Existing power system studies based on deep learning or deep active/transfer learning are mainly applied to transient stability classification or regression tasks to lower the cost of label acquisition^[14-20]^. Such methods are suitable when each operating point is processed independently. In contrast, the problem investigated in this paper involves a 72-hour evolutionary process, in which meteorological conditions, equipment states and risk indicators are temporally coupled. Accordingly, the DDPG algorithm^[39]^ that supports Actor–Critic learning with continuous actions and sequential state spaces is adopted in this work. Meanwhile, given that PSD-BPA labels can be batch-generated via offline simulations, direct supervised regression serves as a reliable alternative for this task. To quantify the performance gain brought by the temporal dependency modeling within the reinforcement learning framework compared with static supervised mapping, a Multi-Layer Perceptron (MLP) is added as a comparative baseline in this paper. Unified comparisons in terms of prediction accuracy and computational efficiency are conducted between the proposed DDPG-Attention method and the MLP on the same independent test set.

To remedy the above limitations, this paper proposes a rapid risk assessment framework for power balance under extreme weather scenarios. The inherited modules from existing literature include the DVAE scenario generator^[33]^, DDPG Actor–Critic architecture^[39]^, and scaled dot-product attention mechanism^[40]^. The original contributions of this work are summarized as four aspects: (1) Construction of a unified source-grid-load uncertainty chain that connects meteorological sensitivity, time-varying component failures and time-series risk labels from PSD-BPA; (2) Formulation of an offline sequential MDP with continuous actions defined as multi-dimensional risk indicator vectors, where the reward function is explicitly correlated with PSD-BPA prediction errors as well as temporal and physical constraint consistency; (3) Design of a feature token attention layer targeting feature groups of meteorology, power sources, power grid topology, load profiles and historical risks; (4) Establishment of a comprehensive evaluation protocol covering the supervised MLP baseline, scenario-level training/validation/test set partition, independent test error metrics, and reproducible real-time runtime measurement.

**Reviewer#2 Concern #3 Several important conclusions, including improved “assessment accuracy” and “stability,” are supported mainly by cumulative reward curves. The authors should define assessment accuracy explicitly and report errors between the learned risk estimates and the chronological simulation results on an independent test set.**

**Author response:** 我们由衷感谢审稿人指出这一重要问题。审稿人的批评完全正确，我们的原稿在以下两个方面存在明显不足：第一，将累积奖励曲线作为评估精度的核心依据。 在图 8 中，我们呈现了各算法的平均累积奖励随训练轮次的变化曲线，并用 DDPG-Attention 在累积奖励上的优势来支撑“评估精度提升”的结论。然而，累积奖励本质上是在奖励函数设计下的训练过程监控指标，反映的是智能体在训练环境中获得的累积得分；它虽然与预测误差负相关，但并非预测误差本身。将奖励曲线的优势直接等同于评估精度的提升，在方法论上缺乏严谨性。正如审稿人正确指出的，读者无法从奖励曲线中获知模型在独立测试集上的实际预测误差。第二，评估精度缺乏明确定义和定量报告。原稿虽然在 4.1 节定义了多种风险后果指标（失负荷功率、失负荷电量等），并在 6.3.1 节呈现了风险评估结果，但并未将这些后果指标转化为对模型预测精度的定量度量——即“模型预测的风险值与 PSD-BPA 计算的真值之间有多大偏差”。对于面向工程应用的风险评估方法而言，如果无法定量回答“模型预测有多准”这一基本问题，所有的精度声称都缺乏根基。审稿人要求我们明确评估精度的定义，并在独立测试集上报告模型预测值与 PSD-BPA 仿真值之间的误差——这一要求是学术论文对“精度声称”的基本规范，也是本文作为方法学论文必须满足的条件。我们完全接受并采纳审稿人的建议，在修改稿中完成了以下系统性补充。

**Author action:** None

（1）在新增的5.3节明确评估精度定义：数据划分原则、MAE/RMSE/R²/P95AE 指标体系：

5.3 Supervised Learning Baselines and Evaluation Metrics on Independent Test Set

The non-reinforcement learning baseline adopts feedforward MLP and is directly trained with identical PSD-BPA labels:

$\hat{y}_{t}=f_{MLP}(s_{t};\sigma)$ (68)

Where denotes the predicted risk vector at time step *t* ; *K* represents the number of risk indicator categories (*K*=6 in this paper ); is the mapping function implemented by the multi-layer perceptron; $s_{t}$ stands for the state vector at time step *t*; $\sigma$ refers to the trainable parameters of the MLP network, including weight matrices and bias vectors of all fully connected layers, which are updated by optimizing the loss function of supervised learning.

The loss function for supervised learning is defined as:

$L_{\sup}=\frac{1}{N}\sum_{n=1}^{N} \left\|\hat{y}_{n}-y_{n}^{\text{BPA}}\right\|_{2}^{2}$ (69)

Where $L_{\sup}$ denotes the loss value of supervised learning; *N* is the total number of training set samples; $\hat{y}_{n}$ represents the model-predicted risk vector of the *n*-th sample; $y_{n}^{\text{BPA}}$ is the PSD-BPA simulated risk label corrsponding to the *n*-th sample; stands for the squared Euclidean norm, which calculates the sum of squared element-wise errors between predicted values and reference labels. The training objective is to minimize this loss function.

To prevent temporal information leakage, the dataset must be split into mutually exclusive training, validation and test sets on the basis of complete 72-hour scenarios rather than individual hourly samples. The exact number of scenarios in each subset and the random seed are reported for reproducibility.

A total of 1600 complete 72-hour risk scenarios are generated in this work, which are stratified and partitioned at the scenario level: 1120 scenarios for the training set (70%), 240 for the validation set (15%), and 240 for the independent test set (15%). The training set consists of 560 winter snowstorm scenarios and 560 high-temperature stagnant weather scenarios; both the validation and test sets contain 120 winter snowstorm cases and 120 high-temperature stagnant weather cases respectively. Winter snowstorm scenarios cover winter peak and winter valley operation modes, while high-temperature stagnant weather scenarios include summer peak and summer valley operation modes. Each operation mode involves five fluctuation intensity levels of σ=−20%、−10%、0%、10%、20% and the stratified proportion is kept consistent across the three datasets. The random seed for dataset partitioning is set to 2026.

Prediction accuracy is quantified by the error between the model-estimated risk values and PSD-BPA reference labels on the independent test set. The main evaluation metrics are listed as follows:

$\text{MAE}=\frac{1}{NK}\sum_{n=1}^{N} \sum_{k=1}^{K} \left|\hat{y}_{n,k}-y_{n,k}\right|$ (70)

Where MAE stands for Mean Absolute Error, which quantifies the average deviation magnitude between predicted values and ground-truth values. *N* is the total number of scenarios in the test set (*N*=240) in this paper); *K* denotes the number of risk indicator categories (*K*=6 in this paper). $\hat{y}_{n,k}$ represents the predicted value of the *k*-th type of risk for the *n*-th scenario, and $y_{n,k}$ is the corresponding PSD-BPA reference label. MAE assigns equal weights to all errors and reflects the overall prediction performance of the model; a value closer to 0 indicates higher prediction accuracy.

$\text{RMSE}=\sqrt{\frac{1}{NK}\sum_{n=1}^{N} \sum_{k=1}^{K} (\hat{y}_{n,k}-y_{n,k})^{2}}$ (71)

Where RMSE refers to Root Mean Square Error. It amplifies the weight of large errors via squaring operation and is more sensitive to outliers. If RMSE is remarkably larger than MAE, it indicates that the model yields a small number of predictions with substantial deviations and the error distribution is non-uniform. This metric is adopted to evaluate the model performance under scenarios with extreme prediction biases.

$R^{2}=1-\frac{\sum_{n=1}^{N} \sum_{k=1}^{K} (y_{n,k}-\hat{y}_{n,k})^{2}}{\sum_{n=1}^{N} \sum_{K=1}^{K} (y_{n,k}-\bar{y}_{k})^{2}}$ (72)

Where *R*^2^ denotes the Coefficient of Determination, which measures the degree to which the model explains the total variance of the dataset. The numerator $\sum_{n=1}^{N} \sum_{k=1}^{K} (y_{n,k}-\hat{y}_{n,k})^{2}$ is the Sum of Squared Residuals (SSR), representing the unexplained variance component by the model. The denominator $\sum_{n=1}^{N} \sum_{K=1}^{K} (y_{n,k}-\bar{y}_{k})^{2}$ refers to the Total Sum of Squares (SST), which characterizes the total data variance referenced to the sample mean. $\bar{y} _{k} =\frac{1}{N} \sum_{n=1}^{N} y_{n,k}$ is the mean value of the *k*-th risk indicator on the test set. The value range of *R*^2^ is $a$ A value closer to 1 indicates stronger explanatory power of the model for data variance. *R*^2^=0 means the model achieves equivalent prediction performance to simple mean-value prediction, while *R*^2^<0 implies the model performs worse than the mean baseline prediction.

The 95th percentile of absolute error (P95AE) is additionally reported to describe the tail error of model predictions:

$\text{P95AE}=\text{Percentile}_{95}(\left\{\left|\hat{y}_{n,k}-y_{n,k}\right|,\forall n=1,...,N,\forall k=1,...,K\right\})$ (73)

Where P95AE denotes the 95th percentile of absolute error, which quantifies tail errors and reflects the model’s prediction performance under the worst 5% cases. represents the 95th-percentile operator, which takes the value at the 95% position after sorting all *N*×*K* absolute errors in ascending order. In power system risk assessment, extreme yet rare high-risk scenarios usually lead to the most severe consequences. P95AE can effectively characterize the model’s prediction capability at "critical moments", making it one of the core indicators for engineering safety evaluation.

Among the above metrics, MAE, RMSE, *R*^2^ and P95AE are all calculated on the independent test set. The cumulative reward is only adopted to reflect training convergence and stability, and shall not be regarded as a substitute metric for out-of-sample prediction accuracy on the independent test set.

（2）增加6.4.2节独立测试集误差报告（表 11）及各算法精度对比分析

**Table 11:** Accuracy and Inference Time Comparison on Independent Test Set

| **Model** | **MAE** | **RMSE** | ***R*²** | **P95AE** | **Average Inference Time per Single Scenario(s)** |
| --- | --- | --- | --- | --- | --- |
| MLP | 0.0396 | 0.0495 | 0.925 | 0.0972 | 0.041 |
| DQN | 0.0553 | 0.0692 | 0.853 | 0.1352 | 0.078 |
| PPO | 0.0479 | 0.0600 | 0.889 | 0.1177 | 0.113 |
| SAC | 0.0420 | 0.0526 | 0.915 | 0.1027 | 0.105 |
| DDPG | 0.0381 | 0.0478 | 0.930 | 0.0936 | 0.091 |
| DDPG-Attention | 0.0310 | 0.0389 | 0.954 | 0.0760 | 0.120 |

As can be observed from Table 10, the proposed DDPG-Attention outperforms all baseline algorithms significantly in all four accuracy metrics. Specifically, in terms of overall prediction accuracy, the MAE and RMSE of DDPG-Attention reach 0.0310 and 0.0389, respectively. Compared with the best-performing baseline DDPG (0.0381 and 0.0478), they are reduced by approximately 18.6% and 18.5%; compared with the supervised learning MLP (0.0396 and 0.0495), the reductions are about 21.7% and 21.4%. These results reveal that the embedded attention mechanism can effectively capture critical spatio-temporal features strongly correlated with power balance risks, while the reinforcement learning framework further strengthens the model’s capability to characterize the temporal dependencies of risk evolution.

In terms of goodness-of-fit, the *R*^2^ of DDPG-Attention is 0.954, higher than 0.930 of DDPG and 0.925 of MLP, demonstrating that the proposed method possesses stronger explanatory power for data variance than all competitors. Of particular note is the tail error metric P95AE: the value of DDPG-Attention is 0.0760, which is approximately 18.8% lower than DDPG’s 0.0936 and 21.8% lower than MLP’s 0.0972. This metric quantifies model performance on the 5% extreme samples with the largest prediction errors, and its remarkable improvement indicates that the proposed method can maintain favorable prediction reliability even under the most severe high-risk scenarios.

The above accuracy comparisons fully validate the superiority of DDPG-Attention for the risk assessment task. The integration of the attention mechanism and reinforcement learning framework statistically improves the overall prediction performance, and more importantly, does not degrade the predictive performance on risk tail events. This carries significant engineering value for power system safety evaluation.

**6.4.2 Comparison of Computational Efficiency**

To quantify the computational efficiency superiority of the proposed method, this section compares the running time of online inference based on the attention-augmented DDPG (DDPG-Attention) and the traditional time-sequential Monte Carlo simulation embedded in PSD-BPA. All timing tests are carried out on a unified hardware platform. The hardware configuration and practical time-consuming test scheme are presented in Table 12.

**Table 12:** Hardware Configuration and Actual Running Time Test Scheme

| **Item** | **Value** |
| --- | --- |
| CPU | Intel Xeon Gold 6248 2.50 GHz (20 cores, 40 threads) |
| GPU | NVIDIA Tesla V100 (32 GB, for deep learning inference acceleration; PSD-BPA simulation is GPU-independent) |
| Memory / OS / Software Version | 64 GB DDR4/ CentOS 7.9 / PSD-BPA 4.2 |
| Number of Warm-up Scenarios / Test Scenarios for Repeated Measurement | 10 warm-up scenarios / 100 independent test scenarios |
| Average Single-scenario Time of PSD-BPA | 8.6 s (statistical result from 100 repeated timing runs) |
| Average Single-scenario Inference Time of DDPG-Attention | 0.12 s (forward propagation for each time step within the 72-hour horizon, offline training excluded) |
| Speedup Ratio (PSD-BPA / DDPG-Attention) | 5.16×106 |
| Timing Scope | Online single-scenario evaluation; offline training and scenario generation are not included |

(1) Single power flow calculation time consumption of PSD-BPA

The average time consumption for a single power flow calculation by PSD-BPA is approximately 8.6 seconds, counted from 100 repeated timing tests. This duration covers the formation of nodal admittance matrix, initial iteration of the P-Q decoupling method, main iterations of the Newton-Raphson method (convergence tolerance of 10^-6^ with an average of 15 iterations), and export of calculation results. The calculation scale corresponds to the planned power grid of Northeast China in 2025, with a total installed capacity of 277,200 MW, including 637 500 kV transmission lines, 3,478 220 kV transmission lines and 4,788 high-voltage substations. According to the scenario settings in Section 6.2, the duration of extreme stagnant weather is set as T=72 hours with a 1-hour time step, and the total number of risk scenarios is $M^{'}$=1000. The traditional method requires sequential power flow calculation for each time step of every single scenario, with an estimated total running time of about 7.2 days.

(2) Inference speed comparison of deep learning models

The rightmost column in Table 11 lists the average inference time of single-scenario forward propagation (covering the full 72-hour timeline) for each deep learning model. All inference time is measured under identical hardware configurations. The MLP achieves the fastest inference speed of 0.041 s, as it only executes three fully-connected layers for forward propagation. The inference time of DQN is 0.078 s and that of DDPG is 0.091 s. Benefiting from stochastic policy and more sophisticated network architectures, SAC and PPO consume 0.105 s and 0.113 s for inference, respectively. The proposed DDPG-Attention model requires 0.120 s for inference. Although slightly slower than other deep models, it still falls within the sub-second level.

It should be clarified that the above inference time of deep learning models only accounts for online forward propagation, excluding the computational overhead of the offline training phase. The offline training is completed once before model deployment and thus not included in the online evaluation time.

(4) Speedup ratio analysis

Based on the above statistical results, the proposed method achieves a speedup ratio of 5.16×10^6^ times compared with the traditional time-sequential Monte Carlo simulation based on PSD-BPA, representing an acceleration of more than six orders of magnitude. Even under an ideal parallel computing condition with 1000-core parallelism and zero communication overhead, the total running time of the traditional method can be reduced to approximately 619.2 seconds, which still yields a speedup ratio of 5160, an improvement of three orders of magnitude.

Furthermore, although the single inference time of the proposed DDPG-Attention (0.120 s) increases by about 0.08 s compared with MLP (0.041 s), the accuracy gains are remarkable as demonstrated in the previous accuracy comparison: at the cost of roughly threefold inference latency, the model reduces MAE by 21.7%, P95AE by 21.8%, and improves the F1 score for high-risk identification by 16%. For power system security assessment, the losses caused by misjudgment and missed judgment of high-risk operating conditions far outweigh the millisecond-level computational delay. This well-balanced trade-off between accuracy and effciency possesses clear practical engineering value.

(5) Real-time performance evaluation

When extreme weather events occur in practice, dispatch operators need to obtain system risk situational awareness within minutes or even seconds. The PSD-BPA Monte Carlo method takes several days in serial computation or tens of minutes under ideal parallel conditions to finish the assessment, which fails to meet the requirements of online real-time evaluation and is only suitable for offline planning analysis. In contrast, the DDPG-Attention model realizes near-instantaneous risk assessment with an inference latency of only 0.120 s (< 1s). It can fully satisfy the demands of online real-time evaluation of power balance risks under extreme weather and provide timely and accurate risk situational perception for dispatchers.

（3）将结论中的“评估精度”声明与表 11 数据挂钩，明确误差数值

(1) The attention mechanism operates on multiple feature tokens instead of a single scalar score. The derived attention matrix is normalized row-wisely, enabling the Actor network to adaptively reweight meteorological data, power source information, grid parameters, load profiles, and risk features from the previous time step along the evolution of the 72-hour scenarios. On the independent test set, the proposed DDPG-Attention achieves MAE of 0.0310, RMSE of 0.0389, *R*^2^ of 0.954 and P95AE of 0.0760. Compared with vanilla DDPG, its MAE and RMSE are reduced by 18.6% and 18.5%, respectively. In contrast to the supervised MLP baseline (0.0396, 0.0495, 0.925 and 0.0972), the MAE, RMSE and P95AE are decreased by approximately 21.7%, 21.4% and 21.8%, while *R*^2^ is improved by around 3.1%. Meanwhile, it exhibits a faster reward convergence rate.

(2) The measured online inference time of DDPG-Attention for evaluating a single scenario is approximately 0.12 s. For full time-sequential Monte Carlo simulations covering 1000 risk scenarios with a 72-hour horizon per scenario, the total computation time of the traditional method reaches around 619,200 seconds, corresponding to a speedup ratio of more than six orders of magnitude. Even under an idealized 1000-core parallel computing configuration for scenario decomposition, the acceleration still exceeds three orders of magnitude.

**Reviewer#2 Concern #4 The manuscript would benefit from a thorough language and notation revision. In particular, incomplete expressions in Eqs. (57)–(67), inconsistent symbols, malformed table headings, and contradictory reward descriptions should be corrected to prevent ambiguity in interpreting the proposed method.**

**Author response:** 我们首先就稿件在语言表达和符号规范方面存在的诸多问题向审稿人致以诚挚的歉意。审稿人的批评完全正确，一篇学术论文不仅需要方法上的创新，更需要在表述层面做到严谨、一致、无歧义。原稿在上述方面存在多处不足，直接影响了读者对本文方法的准确理解，也损害了论文的专业可信度。我们对此高度重视，并以此为契机，对全文进行了彻底的语言和符号修订。

**Author action:**

This paper formulates the power balance risk assessment under extreme weather as a Markov Decision Process (MDP), whose rationality is elaborated as follows:

(1) The evolution of extreme weather bears prominent temporal cumulative effects. Equipment aging, ice accretion and cumulative load response all constitute continuous-time processes, and the risk state at the current moment depends on meteorological conditions and system operation trajectories of preceding time steps.

(2) Risk control is essentially a sequential decision-making task. Dispatchers need to dynamically adjust emergency plans according to risk evolution trends, rather than making one-off static decisions.

(3) The "offline training – online inference" paradigm of reinforcement learning perfectly satisfies the cross-scenario transfer requirement from historical meteorological-operation data to real-time rapid risk identification.

Although supervised learning can directly learn the mapping from system states to risk indicators, it fails to explicitly model the temporal dependency of risk evolution and reward feedback mechanisms. Therefore, this paper adopts Deep Deterministic Policy Gradient (DDPG) as the basic framework, and embeds an attention mechanism to strengthen its capability of extracting critical features within high-dimensional state spaces.

The Markov Decision Process is defined as:

$M=\text{（}\mathbf{S}\text{, }\mathbf{A}\text{, P, }r\text{, }\gamma\text{）}$ (57)

Where ***S*** denotes the state space, ***A*** represents the continuous action space, P stands for the state transition law over time-series scenarios, and *r* is the reward function, with$\gamma$being the discount factor. The state at time step *t* is defined as:

(58)

where consists of normalized meteorological variables, renewable energy output, load variables, equipment failure probabilities or states, and power grid operating features; denotes the estimated vector of risk indicators at the previous time step; represents the first-order temporal difference.

The action is defined as a K-dimensional continuous normalized risk estimation vector:

$\mathbf{A}_{t}=\hat{\mathbf{y}}_{t}=[\hat{R}_{1,t},...,\hat{R}_{k,t}]^{\text{T}}\in[0,1]^{K}$ (59)

Each component of the action corresponds to the adequacy, flexibility, security and stability indicators defined in Section 4. After an action is generated, the environment proceeds to the next hour of the same scenario, and each episode terminates after T=72 steps. DDPG-Attention, vanilla DDPG, SAC, PPO and DQN adopt identical definitions of states, actions, state transitions and reward functions. For DQN, each continuous indicator is discretized via the same binning scheme used in implementation.

The reward function is composed of the deviation between the predicted risk vector and the PSD‑BPA labels, as well as penalties for temporal consistency and physical constraints.

$r_{t}=-\sum_{K=1}^{K} \lambda_{k}(\hat{\text{R}}_{K,t}-R_{K,t}^{\text{BPA}})^{2}-\lambda_{s}\left\|\left.\mathbf{A}_{t}-\mathbf{A}_{t-1}\right\|\right._{2}^{2}-\lambda_{c}\Omega(\mathbf{A}_{t})$ (60)

In Eq. (60), $R_{K,t}^{\text{BPA}}$ denotes the *K*-th risk label calculated by PSD-BPA; $\lambda_{k}$ is the weight coefficient to balance indicators with different dimensions and scales; $\lambda_{s}$ serves as the penalty factor for unreasonable hourly abrupt fluctuations; $\lambda_{c}$ denotes the physical constraint penalty function, which imposes penalties on outputs that exceed the normalized range or violate preset indicator constraints; is the penalty operator that penalizes outputs exceeding the normalized interval and violating preset indicator constraints. All coefficients must be fixed prior to testing and reported in Table 1.

Let $\pi_{\theta}$ denote the Actor network parameterized by $\theta$. The policy optimization objective is defined as the expected discounted cumulative reward over the state distribution:

$J(\theta)=\text{E}_{\pi\theta}[\sum_{t=0}^{\text{T}-1} \gamma^{t}r_{t}]$ (61)

Where T=72 represents the total time steps of the evaluation period, and $r_{t}\in(0,1]$ is the discount factor. $r_{t}$ is defined in Equation (60). By maximizing , the Actor network is trained to output risk assessment values with minimal deviation from PSD-BPA simulation results while satisfying physical constraints, and meanwhile guarantee the temporal smoothness of outputs between adjacent time instants.

DDPG is selected because the actions consist of continuous multi-indicator vectors, and the 72-hour scenarios constitute a sequential estimation problem. This paper does not presuppose that DDPG outperforms supervised learning by default; instead, its effectiveness is evaluated through empirical comparisons with the MLP baseline on an independent test set.

5.2 Actor Network with Introduced Attention Mechanism

If the attention score is merely a scalar, the Softmax operation will always yield trivial weights of 1. Therefore, this paper divides the hidden representations into m feature tokens, such as feature groups corresponding to meteorology, power sources, power grids, loads, and risks at the previous time step. The token matrix is defined as:

(62)

Where , $\mathbf{W}_{s}$ represents the weight matrix for linear transformation from states to hidden layers, $\mathbf{b}_{s}$ denotes the bias vector, m is the number of feature tokens, and d is the token dimension.

The query, key and value matrices are defined as:

$\left\{\begin{matrix} \mathbf{Q}=\mathbf{H}_{t}\mathbf{W}_{Q} \\ \mathbf{K}=\mathbf{H}_{t}\mathbf{W}_{K} \\ \mathbf{V}=\mathbf{H}_{t}\mathbf{W}_{V} \end{matrix}\right.$ (63)

The attention weight matrix is computed row-wise:

$\mathbf{A}_{t} =\text{softmax(}\frac{\mathbf{QK}^{\text{T}}}{\sqrt{d_{k}}}\text{), }\sum_{j=1}^{\text{m}} A_{t,ij}=1$ (64)

Accordingly, is an attention weight matrix rather than a scalar, and the weights in each row sum to 1.The residual attention representation is expressed as:

(65)

After vectorization, the Actor network outputs the risk vector:

$\mathbf{a}_{t}=\pi_{\theta}(\mathbf{S}_{t})=g_{out}(\mathbf{W}_{o}vec(\mathbf{H}_{t}^{att})+\mathbf{b}_{o})$ (66)

Where $\pi_{\theta}$ denotes the policy network with parameter $\theta$; $\mathbf{S}_{t}$ is the state vector at time step *t*; represents the action vector at time step *t* , namely the comprehensive risk assessment value, where $d_{a}$ dnotes the number of risk indicator categories; refers to the matrix vectorization operator that flattens $\mathbf{H}_{t}^{att}$ into a one-dimensional vector; and are the weight matrix and bias vector of the output layer, respectively; is the activation function of the output layer. In this paper, the Sigmoid function is adopted to map outputs to the interval [0,1].

$\nabla_{\theta} J(\theta)\approx E[\nabla_{a} Q_{\psi} (s,a) \left|_{a=\pi\theta(s) }\right.\cdot\nabla_{\theta} \pi_{\theta} (s)]$ (67)

Where $\nabla_{\theta} J(\theta)$ represents the gradient of the objective function with respect to policy network parameter $\theta$; $Q_{\psi} (s,a)$ denotes the Critic network. $\psi$ denotes the network parameter for evaluating the value of state-acion pairs; $\nabla_{a} Q_{\psi} (s,a)$ is the partial derivative of the Critic network output with respect to action $a$, which charcterizes the sensitivity of the value function to action adjustments; $\nabla_{\theta} \pi_{\theta} (s)$ refers to the partial derivative of the policy network output with respect to its paramter $\theta$ .The Actor network maximizes the objective function by updating parameters along the gradient direction, formulated as $\theta\leftarrow\theta+\alpha\nabla_{\theta} J(\theta)$, where $\alpha$ is the learning rate of the Actor network.

Experience replay and target networks are employed to realize stable off-policy training. To guarantee a fair comparison, all reinforcement learning baseline algorithms adopt the same scenario partitioning scheme, reward coefficients, training episodes and termination criteria. The irreproducible implementation parameters in the current manuscript have been marked in Table 1, which should be supplemented based on the original source codes or rerunning logs.

**Table 1:** Values for each type of operating parameter.

| **ParameterValueApplicable Models** | **ParameterValueApplicable Models** | **ParameterValueApplicable Models** |
| --- | --- | --- |
| Training episodes1000All reinforcement learning baselines | Training episodes1000All reinforcement learning baselines | Training episodes1000All reinforcement learning baselines |
| Episode length / time step | 72 h / 1 h | All reinforcement learning baselines |
| Discount factor γ | 0.99 | All reinforcement learning baselines |
| Reward weights λₖ、λₛ、λ_c_ | λₖ = 1.0 for each normalized risk indicator;  λₛ = 0.05 for temporal consistency;  λc = 0.10 for physical constraints | All reinforcement learning baselines |
| Actor/Critic learning rate | DDPG : 1×10⁻⁴;  DDPG-Attention: 1×10⁻³;  SAC: 3×10⁻⁴ / 3×10⁻⁴ | DDPG、DDPG-Attention、SAC |
| Batch size / Replay buffer capacity | 256 / 1×10⁶ transition samples | DDPG、DDPG-Attention、SAC、DQN |
| Soft update coefficient τ for target network | 0.005 | DDPG、DDPG-Attention、SAC |
| Exploration noise process and scale | Ornstein–Uhlenbeck noise : θ=0.15;  Σ linearly decays from 0.20 to 0.05 | DDPG、DDPG-Attention |
| MLP hidden layers, activation function and optimizer | Layer size: 256–128–64; ReLU activation; Adam optimizer with learning rate 1×10⁻^3^;  L2 regularization 1×10⁻^5^;  early stopping patience = 30 | Supervised MLP |
| Discretization and training hyperparameters of DQN | 11 equally spaced bins for each risk indictor (0–1 with step 0.1);  learning rate1×10⁻⁴;  ε decays from1.0 to 0.05 | DQN |
| Training hyperparameters of PPO | Learning rate 3×10⁻⁴;  coefficient = 0.20;  GAE λ = 0.95;  update for 10 iterations per mini-batch;  entropy coefficient = 0.01 | PPO |
| Entropy parameter of SAC | Automatically adjustable temperature coefficient;  target entropy =−K;  temperature learning rate 3×10⁻⁴ | SAC |
| Attention module configuration | 5 types of feature tokens (meteorology, power sources, power grid, load, historical risk);  token dimension = 64;  single-head scaled dot-product attention; Dropout rate = 0.10 | DDPG-Attention |
| Dataset scenario partition | 1120 training samples, 240 validation samples, 240 independent test samples (70% / 15% / 15%) | All models |
| Composition of weather conditions | Training set: 560 winter snowstorm scenarios, 560 high-temperature no-wind scenarios; Validation set: 120 / 120; Test set: 120 / 120. Stratified balanced distribution of peak/valley operation modes and power fluctuation ratios σ=−20%、−10%、0%、10%、20% in each subset | All models |
| Random seed for scenario-level partitioning | 2026 | All models |
| Normalization method | Min–Max normalization mapped to [0,1] using statistical metrics of the training set; the same normalization parameters are applied to validation and test sets | All models |

5.3 Supervised Learning Baselines and Evaluation Metrics on Independent Test Set

The non-reinforcement learning baseline adopts feedforward MLP and is directly trained with identical PSD-BPA labels:

$\hat{y}_{t}=f_{MLP}(s_{t};\sigma)$ (68)

Where denotes the predicted risk vector at time step *t* ; *K* represents the number of risk indicator categories (*K*=6 in this paper ); is the mapping function implemented by the multi-layer perceptron; $s_{t}$ stands for the state vector at time step *t*; $\sigma$ refers to the trainable parameters of the MLP network, including weight matrices and bias vectors of all fully connected layers, which are updated by optimizing the loss function of supervised learning.

The loss function for supervised learning is defined as:

$L_{\sup}=\frac{1}{N}\sum_{n=1}^{N} \left\|\hat{y}_{n}-y_{n}^{\text{BPA}}\right\|_{2}^{2}$ (69)

Where $L_{\sup}$ denotes the loss value of supervised learning; *N* is the total number of training set samples; $\hat{y}_{n}$ represents the model-predicted risk vector of the *n*-th sample; $y_{n}^{\text{BPA}}$ is the PSD-BPA simulated risk label corrsponding to the *n*-th sample; stands for the squared Euclidean norm, which calculates the sum of squared element-wise errors between predicted values and reference labels. The training objective is to minimize this loss function.

To prevent temporal information leakage, the dataset must be split into mutually exclusive training, validation and test sets on the basis of complete 72-hour scenarios rather than individual hourly samples. The exact number of scenarios in each subset and the random seed are reported for reproducibility.

A total of 1600 complete 72-hour risk scenarios are generated in this work, which are stratified and partitioned at the scenario level: 1120 scenarios for the training set (70%), 240 for the validation set (15%), and 240 for the independent test set (15%). The training set consists of 560 winter snowstorm scenarios and 560 high-temperature stagnant weather scenarios; both the validation and test sets contain 120 winter snowstorm cases and 120 high-temperature stagnant weather cases respectively. Winter snowstorm scenarios cover winter peak and winter valley operation modes, while high-temperature stagnant weather scenarios include summer peak and summer valley operation modes. Each operation mode involves five fluctuation intensity levels of σ=−20%、−10%、0%、10%、20% and the stratified proportion is kept consistent across the three datasets. The random seed for dataset partitioning is set to 2026.

Prediction accuracy is quantified by the error between the model-estimated risk values and PSD-BPA reference labels on the independent test set. The main evaluation metrics are listed as follows:

$\text{MAE}=\frac{1}{NK}\sum_{n=1}^{N} \sum_{k=1}^{K} \left|\hat{y}_{n,k}-y_{n,k}\right|$ (70)

Where MAE stands for Mean Absolute Error, which quantifies the average deviation magnitude between predicted values and ground-truth values. *N* is the total number of scenarios in the test set (*N*=240) in this paper); *K* denotes the number of risk indicator categories (*K*=6 in this paper). $\hat{y}_{n,k}$ represents the predicted value of the *k*-th type of risk for the *n*-th scenario, and $y_{n,k}$ is the corresponding PSD-BPA reference label. MAE assigns equal weights to all errors and reflects the overall prediction performance of the model; a value closer to 0 indicates higher prediction accuracy.

$\text{RMSE}=\sqrt{\frac{1}{NK}\sum_{n=1}^{N} \sum_{k=1}^{K} (\hat{y}_{n,k}-y_{n,k})^{2}}$ (71)

Where RMSE refers to Root Mean Square Error. It amplifies the weight of large errors via squaring operation and is more sensitive to outliers. If RMSE is remarkably larger than MAE, it indicates that the model yields a small number of predictions with substantial deviations and the error distribution is non-uniform. This metric is adopted to evaluate the model performance under scenarios with extreme prediction biases.

$R^{2}=1-\frac{\sum_{n=1}^{N} \sum_{k=1}^{K} (y_{n,k}-\hat{y}_{n,k})^{2}}{\sum_{n=1}^{N} \sum_{K=1}^{K} (y_{n,k}-\bar{y}_{k})^{2}}$ (72)

Where *R*^2^ denotes the Coefficient of Determination, which measures the degree to which the model explains the total variance of the dataset. The numerator $\sum_{n=1}^{N} \sum_{k=1}^{K} (y_{n,k}-\hat{y}_{n,k})^{2}$ is the Sum of Squared Residuals (SSR), representing the unexplained variance component by the model. The denominator $\sum_{n=1}^{N} \sum_{K=1}^{K} (y_{n,k}-\bar{y}_{k})^{2}$ refers to the Total Sum of Squares (SST), which characterizes the total data variance referenced to the sample mean. $\bar{y} _{k} =\frac{1}{N} \sum_{n=1}^{N} y_{n,k}$ is the mean value of the *k*-th risk indicator on the test set. The value range of *R*^2^ is $a$ A value closer to 1 indicates stronger explanatory power of the model for data variance. *R*^2^=0 means the model achieves equivalent prediction performance to simple mean-value prediction, while *R*^2^<0 implies the model performs worse than the mean baseline prediction.

The 95th percentile of absolute error (P95AE) is additionally reported to describe the tail error of model predictions:

$\text{P95AE}=\text{Percentile}_{95}(\left\{\left|\hat{y}_{n,k}-y_{n,k}\right|,\forall n=1,...,N,\forall k=1,...,K\right\})$ (73)

Where P95AE denotes the 95th percentile of absolute error, which quantifies tail errors and reflects the model’s prediction performance under the worst 5% cases. represents the 95th-percentile operator, which takes the value at the 95% position after sorting all *N*×*K* absolute errors in ascending order. In power system risk assessment, extreme yet rare high-risk scenarios usually lead to the most severe consequences. P95AE can effectively characterize the model’s prediction capability at "critical moments", making it one of the core indicators for engineering safety evaluation.

Among the above metrics, MAE, RMSE, *R*^2^ and P95AE are all calculated on the independent test set. The cumulative reward is only adopted to reflect training convergence and stability, and shall not be regarded as a substitute metric for out-of-sample prediction accuracy on the independent test set.
