# Supplementary material for: A fast risk assessment method for power balance under extreme weather conditions based on deep reinforcement learning with a coupled attention mechanism
Source: PLoS One. 2026 Oct 8;21(10):e0359101. doi: 10.1371/journal.pone.0359101 (PMC13649036; doi:10.1371/journal.pone.0359101)
Supplement: Supplementary file 2 [file pone.0359101.s003.docx]

**Original Manuscript Number:** PONE-D-26-32244

**Original Article Title: “A Fast Risk Assessment Method for Power Balance Under Extreme Weather Conditions Based on Deep Reinforcement Learning with a Coupled Attention Mechanism**”

Dear Editor,

We would like to express our sincere gratitude to you and the reviewers for the valuable time and constructive comments devoted to our manuscript (Manuscript Number: PONE-D-26-32244). The insightful suggestions have been instrumental in helping us significantly improve the quality of our work.

We have carefully considered all the comments and have revised the manuscript accordingly. Our point-by-point responses to each comment are detailed below.

We believe the manuscript is now strengthened and hope it meets the journal's standards for publication.

Thank you for your consideration.

Best regards,

< Wang Hang > et al.

**Reviewer#1, Concern #1: The submission contains two manuscript files that are not equivalent in content. The file labeled as the clean manuscript ends at Section 6.4 "Algorithm Comparison Results" (53 pages) and does not include the supervised MLP baseline comparison, Tables 9 through 12, the hardware/inference timing analysis, or the revised Conclusion that appear in the file labeled "Revised Manuscript with Track Changes" (60 pages). The two files also differ in the reported reward trajectory in Figure 8's description ("increases from 7.5 to approximately 0.1" in the shorter file versus "increases from -8 to approximately 0.2" in the longer file) and in the author list and corresponding-author contact (the shorter file has six authors and a QQ.com email; the longer file adds a seventh author, HeXiang Niu, and lists a different corresponding email). A single manuscript file containing all of this content should be provided, with one author list, one corresponding address, and one reward trajectory in Figure 8, before the paper can be evaluated as one document, since the file intended for production cannot omit content that the paper's own conclusions rely on.**

**Author response:** 非常感谢您指出我们在稿件提交过程中的这一严重疏忽。我们完全同意您的意见，提交两个内容不等同的稿件文件是非常不专业的做法，给您和编辑部的审稿工作带来了极大的困扰和额外的负担。对此，我们深表歉意，并在此向您致以最诚挚的歉意。

在收到您的意见后，我们立即对全部文件进行了仔细的核对和审查，现已确认问题的根源，并采取了以下修正措施：

**Author action:**

（1）文件整合与内容统一

我们确认，标注为“Revised Manuscript with Track Changes.docx”的较长版本（60页）是完整、正确且最终版的稿件。该版本包含了您在意见中提到的所有内容，包括监督式MLP基线比较、表9至表12、硬件推理时间分析以及全面修订后的结论。之前提交的较短版本“Manuscript”（53页）是我们第一次修改前的初始版本。“Revised Manuscript with Track Changes.docx”是在“Manuscript”的基础上，根据第一次审稿人修改意见基础上修改增加内容后的版本。这次的第二修改，我们将“Revised Manuscript with Track Changes.docx”命名为Manuscript（2）.docx；将第二次修改后的文件命名为“Revised Manuscript with Track Changes（2）.docx”。

（2）作者列表与通讯邮箱统一

作者列表现已统一为较长版本中的全部七位作者。新增的第七位作者何香牛（HeXiang Niu） 是东北电力大学能源与动力工程学院的科研人员，在本研究的数据处理和模型验证环节做出了重要贡献，其署名是合理且必要的。通讯作者邮箱统一更正为 nhxneepu@163.com，这是王航博士在东北电力大学的官方工作邮箱。此前出现的QQ邮箱为个人备用邮箱，现已全部删除，以避免后续混淆。

（3）图8描述校正

感谢您敏锐地发现了两份文件中对图8奖励轨迹描述的不一致。经重新核查实验原始记录，我们确认图8中展示的奖励曲线起点约为-8，最终收敛至约0.2。较短版本中“从7.5增加到约0.1”的描述属于早期草稿中的笔误，与图中实际数据不符。我们已在统一后的稿件中对此进行了修正，确保文字描述与图表数据完全一致。

现已将全部内容整合为一份完整、统一的“清洁稿件”，不再保留任何不完整或过时的版本命名为“Revised Manuscript with Track Changes（2）.docx”。

**Reviewer#1, Concern #2: Within the longer manuscript file, Table 9 (risk values of power shortage by confidence value) and Table 10 (risk values of power shortage under the rainstorm control scheme) share an identical, truncated caption, "Risk values of power shortages by region at different." The captions do not state what varies across rows in each table (confidence level in Table 9, uncertainty source/scheme in Table 10), and the in-text sentence preceding the tables states results are "presented in Tables 8 and 10" while a separate sentence states the confidence-value results are "shown in Table 9," which is confusing on first read. Both captions should be completed and made distinct.**

**Author response:** 非常感谢您再次指出我们在表格标注和正文指引方面的疏漏。表9和表10标题相同且被截断的问题，以及正文中表格引用混乱的现象，确实给您的阅读带来了困扰。这是我们准备稿件时不够细致所致，对此我们深表歉意。我们已对相关问题进行了逐一修正，具体如下：

**Author action:**

将原表10标题的修正为: High risk proportion of various indicators in Liaoning Power Grid of China in various schemes；将各个表格的顺序重新调整如下所示：

To verify the proposed model and method, three evaluation and calculation schemes are designed and simulated in this section, and the corresponding results are presented in Tables 8 and 10. Among them, the risk of power shortage under different confidence levels is listed in Table 9.

Scheme 1: The proposed models and methods were used to conduct a comparative analysis of the system risk levels under extreme conditions corresponding to different confidence values σ.

**Table 8:** High-risk proportion of various indicators in various regions of China with different values of in scheme 1

| **Risk type** | **Confidence value σ/%** | **Proportion of high-risk areas by region /%** | | | |
| --- | --- | --- | --- | --- | --- |
|  |  | Heilongjiang | Jilin | Liaoning | Eastern Inner Mongolia |
| Power deficiency | -20 | 2.78 | 0 | 11.11 | 0 |
|  | -10 | 63.89 | 26.39 | 65.28 | 0 |
|  | 0 | 65.28 | 33.33 | 68.94 | 0 |
|  | 10 | 72.37 | 45.76 | 75 | 1.09 |
|  | 20 | 77.41 | 50.28 | 80 | 3.51 |
| Insufficient spinning reserve | -20 | 11.11 | 0 | 80.56 | 0 |
|  | -10 | 81.94 | 62.5 | 100 | 0 |
|  | 0 | 100 | 88.89 | 100 | 38.89 |
|  | 10 | 100 | 100 | 100 | 55.56 |
|  | 20 | 100 | 100 | 100 | 58.06 |
| Voltage violation | -20 | 0 | 0 | 11.11 | 0 |
|  | -10 | 62.5 | 0 | 68.06 | 0 |
|  | 0 | 97.22 | 12.5 | 87.5 | 0 |
|  | 10 | 98.61 | 50 | 98.61 | 3.09 |
|  | 20 | 88.89 | 54.44 | 100 | 9.51 |
| Power flow violation | -20 | 0 | 0 | 11.11 | 0 |
|  | -10 | 62.5 | 0 | 68.06 | 0 |
|  | 0 | 98.61 | 41.67 | 87.5 | 0 |
|  | 10 | 98.61 | 52.78 | 100 | 0 |
|  | 20 | 98.83 | 58.78 | 100 | 0 |
| Transformer loading violation | -20 | 0 | 0 | 11.11 | 0 |
|  | -10 | 62.5 | 0 | 68.06 | 0 |
|  | 0 | 98.61 | 38.89 | 87.5 | 0 |
|  | 10 | 98.61 | 65.28 | 100 | 0 |
|  | 20 | 95.83 | 72.78 | 100 | 0 |
| Frequency violation | -20 | 0 | 0 | 4.17 | 0 |
|  | -10 | 21.35 | 2.78 | 34.72 | 0 |
|  | 0 | 30.56 | 4.167 | 41.67 | 0 |
|  | 10 | 34.72 | 11.11 | 43.67 | 0 |
|  | 20 | 38.44 | 15.11 | 51.75 | 2.81 |

**Table 9:** Risk values of power shortages by region at different σ

| **Risk type** | **Confidence value σ** | **Heilongjiang** | **Jilin** | **Liaoning** |
| --- | --- | --- | --- | --- |
| Power deficiency | -20% | 1.39×10-4 | 0 | 1.5×10-3 |
|  | -10% | 2.77×10-3 | 0 | 7.24×10-3 |
|  | 0 | 1.88×10-3 | 9.56×10-4 | 4.8×10-3 |
|  | 10% | 5.39×10-3 | 4.28×10-4 | 5.77×10-3 |
|  | 20% | 6.7×10-3 | 5.09×10-3 | 7.55×10-3 |

Scheme 2:Using historical weather data of a certain rainstorm event as input, and adopting the direct failure model of equipment under rainstorm disasters from reference [38] for the environment-dependent failure probability, while keeping the other models as those proposed in this paper, the system risk was assessed.

Scheme 3: Risk scenario uncertainty models were established by considering only the source–load power fluctuations and only the equipment failures, respectively, and the system risk under partial uncertainty was calculated accordingly.

1) Superiority analysis of the proposed assessment method

Observing Table 8, it can be seen that as the confidence value σ increases, the weather becomes more severe, and the risk values of various indicators also increase. When σ increases to 0, the system is exactly at the critical state of abnormal operation, and the high-risk proportion of each indicator increases significantly, indicating that under such extreme conditions, the system is indeed a high-risk scenario. This further verifies that the extreme weather conditions defined based on the normal operating state threshold of the grid, as proposed in this paper, can indeed provide relatively accurate criteria for risk scenario identification in power grids. Moreover, this method has profound implications: by fitting the functional relationship between meteorological elements of different severity levels and the corresponding system operational risks, the risk level of a planned power grid under future extreme weather can be estimated merely by monitoring meteorological factors. This means that rapid identification of extreme weather scenarios and early warning of risks in power grids become feasible.

2) Accuracy analysis of the proposed assessment method

Extreme rainstorms are not included in the results of risk scenario identification in this paper. Therefore, simulations were carried out using historical data of a rainstorm event, and the results are shown in Table 10. Since no fault modeling of grid equipment under rainstorm weather was performed in this paper, and this case was merely used as a control group in the numerical example, the gridside uncertainty was characterized using the grid equipment fault model for rainstorm disasters from reference [38]. From Table 10, it can be seen that the high-risk proportions of various system indicators obtained by Scheme 1 are significantly higher than those obtained by Scheme 2 by tens of times. This indicates that the two types of extreme weather identified and generated for the Northeast China region in this paper can indeed cause considerable impacts on the power system of this region, whereas the un identified rainstorm poses a relatively low threat to the regional grid. Therefore, the scenario identification method proposed in this paper is effective. By fully considering regional climate and grid characteristics, the proposed method can provide adaptive scenario data for risk assessment of the target power grid, thereby helping to improve the accuracy of each calculated risk value.

**Table 10:** High risk proportion of various indicators in Liaoning Power Grid of China in various schemes

| **Scheme** | **Confidence valueσ/%** | **Weather data source** | **Sources of uncertainty** | | **Consequence evaluation platform** | **Proportion of high-risk areas by region /%** | | | | |
| --- | --- | --- | --- | --- | --- | --- | --- | --- | --- | --- |
|  |  |  | Source and load | Grid side |  | Power deficiency | Insufficient spinning reserve | Voltage violation | Power flow violation | Transformer loading violation |
| 1 | 0 | Scenario generation | √ | √ | Planning platform | 68.94 | 100 | 87.5 | 87.5 | 87.5 |
| 2 |  | Historical heavy rain | √ | Reference [30] |  | 6.07 | 6.41 | 2.38 | 2.59 | 2.81 |
| 3 | 0 | Scenario generation | √ | × |  | 66.88 | 98.35 | 79.19 | 80.13 | 78.96 |
|  |  |  | × | √ |  | 62.74 | 90.2 | 86.28 | 84.44 | 84 |

Compared with Scheme 1, the high-risk proportions of various system indicators in Scheme 3 are reduced to varying degrees, with a reduction range of 2% to 9%. This indicates that risk assessment results considering only source load power/grid side failure uncertainties are overly optimistic. It can be seen that comprehensively and accurately characterizing source grid load uncertainties plays an important role in risk assessment work.

**Reviewer#1, Concern #3:The accuracy comparison in Table 11 (MAE, RMSE, R2, P95AE for MLP, DQN, PPO, SAC, DDPG, DDPG-Attention) is reported as a single run with a fixed data-partition random seed (2026). The gap between DDPG-Attention and the next-best baseline (DDPG) is 18.6% in MAE and 18.5% in RMSE, and the gap to SAC is smaller still. With no repeated trials across multiple seeds, no confidence intervals, and no significance test, it is not possible to determine whether these margins reflect the attention mechanism itself or run-to-run training variance, which is a standard concern for stochastic RL training curves.**

**Author response:** 非常感谢您指出我们在实验统计严谨性方面的不足。您指出“由于没有跨多个种子的重复试验、没有置信区间、也没有显著性检验，因此无法确定这些差距反映的是注意力机制本身还是运行间训练方差”，这是一个非常关键且专业的问题，切中了深度强化学习实验方法论的核心。我们完全认同您的观点。在深度强化学习中，算法性能对随机种子高度敏感——同一算法在相同超参数下使用不同随机种子运行，其结果可能呈现出显著差异，仅凭单次运行的结果声称某算法优于另一算法，缺乏必要的统计证据支撑。针对您的意见，我们补充了以下实验和统计分析：

**Author action:**

我们使用10个不同的随机种子（2021–2030），对所有6种算法（MLP、DQN、PPO、SAC、DDPG、DDPG-Attention）分别进行了10次独立训练和评估；采用 Wilcoxon符号秩检验（配对非参数检验）比较DDPG-Attention与次优算法（DDPG）在每个指标上的差异。在6.4.1节、6.4.2节、7 结论部分修改有关多次独立训练后的相关数据，并用红色字体标出：

**6.4.1 Comparison with Supervised Learning Baselines**

To ensure the statistical robustness of the comparison, all six algorithms (MLP, DQN, PPO, SAC, DDPG, and DDPG-Attention) were re-trained and evaluated 10 times independently using different random seeds (2021–2030). The dataset partitioning for each seed was kept identical across all algorithms to ensure a fair comparison. Table 11 reports the mean ± standard deviation of MAE, RMSE, *R*², and P95AE on the independent test set. Furthermore, the Wilcoxon signed-rank test was conducted between DDPG-Attention and the second-best performing algorithm (vanilla DDPG) to assess the statistical significance of the observed performance differences, The significance level is set to $\alpha$=0.05. If *p*<0.05, the null hypothesis is rejected, which demonstrates that the performance improvement brought by the attention mechanism is statistically significant. All error metrics are calculated within the normalized risk indicator space ranging from 0 to 1, with 240 complete 72-hour scenarios adopted as the independent test dataset. The proposed DDPG-Attention achieves the minimum MAE, RMSE and P95AE values as well as the maximum *R*^2^, which demonstrates that the feature token-based attention mechanism can improve the fitting accuracy of multiple risk indicators and enhance the control ability against extreme prediction errors.

**Table 11:** Accuracy and Inference Time Comparison on Independent Test Set

| **Model** | **MAE** | **RMSE** | ***R*²** | **P95AE** | **Average Inference Time per Single Scenario(s)** |
| --- | --- | --- | --- | --- | --- |
| MLP | 0.0398 ± 0.0012 | 0.0500 ± 0.0016 | 0.923 ± 0.004 | 0.0981 ± 0.0035 | 0.041 |
| DQN | 0.0560 ± 0.0021 | 0.0701 ± 0.0025 | 0.849 ± 0.006 | 0.1372 ± 0.0051 | 0.078 |
| PPO | 0.0485 ± 0.0018 | 0.0610 ± 0.0020 | 0.885 ± 0.005 | 0.1195 ± 0.0042 | 0.113 |
| SAC | 0.0425 ± 0.0015 | 0.0531 ± 0.0018 | 0.913 ± 0.004 | 0.1040 ± 0.0038 | 0.105 |
| DDPG | 0.0385 ± 0.0011 | 0.0482 ± 0.0015 | 0.929 ± 0.004 | 0.0951 ± 0.0032 | 0.091 |
| DDPG-Attention | 0.0315 ± 0.0009 | 0.0394 ± 0.0012 | 0.952 ± 0.003 | 0.0772 ± 0.0028 | 0.120 |
| *p-*value | < 0.01 | < 0.01 | < 0.01 | < 0.01 | - |

As can be observed from Table 11, statistical results from repeated experiments with multiple random seeds demonstrate that the proposed DDPG-Attention outperforms all baseline algorithms in terms of the mean values of the four evaluation metrics. Specifically, regarding overall prediction accuracy, the MAE and RMSE of DDPG-Attention are 0.0315 ± 0.0009 and 0.0394 ± 0.0012, respectively. Compared with the best-performing baseline DDPG (0.0385 ± 0.0011 and 0.0482 ± 0.0015), these metrics are reduced by approximately 18.2% and 18.3%, while they decrease by around 20.9% and 21.2% relative to the supervised learning model MLP (0.0398 ± 0.0012 and 0.0500 ± 0.0016), respectively. The Wilcoxon signed-rank test confirms that these improvements are statistically significant (*p-*value < 0.01), demonstrating that the performance gains stem from the attention mechanism rather than random training variance. These results reveal that the embedded attention mechanism can effectively capture critical spatio-temporal features strongly correlated with power balance risks, while the reinforcement learning framework further strengthens the model’s capability to characterize the temporal dependencies of risk evolution. It is worth noting that the accuracy comparison in Table 11 is based on open-loop evaluation for all models, where $\text{R}_{\text{t}\text{-1}}$ is the ground-truth PSD-BPA label. This protocol is adopted to ensure fair comparison of single-step mapping capability. For practical deployment, the model operates in a closed-loop autoregressive manner; the resulting performance degradation is quantified in Section 6.4.3, and the inference speed remains within the sub-second range.

In terms of goodness-of-fit, DDPG-Attention achieves an *R*^2^ of 0.952 ± 0.003, higher than 0.929 ± 0.004 for DDPG and 0.923 ± 0.004 for MLP, indicating that the proposed method possesses a stronger capability to interpret data variations than all comparative models. Particular attention should be paid to the tail error metric P95AE: DDPG-Attention reaches 0.0772 ± 0.0028, which is approximately 18.8% lower than DDPG 0.0951 ± 0.0032 and roughly 21.3% lower than MLP 0.0981 ± 0.0035. This metric quantifies model performance on the 5% extreme samples with the largest prediction errors. The prominent improvement verifies that the proposed method can maintain favorable prediction reliability even under the most severe high-risk scenarios.

The above accuracy comparisons fully validate the superiority of DDPG-Attention for the risk assessment task. The integration of the attention mechanism and reinforcement learning framework statistically improves the overall prediction performance, and more importantly, does not degrade the predictive performance on risk tail events. This carries significant engineering value for power system safety evaluation.

**6.4.2 Comparison of Computational Efficiency**

(4) Speedup ratio analysis

Based on the above statistical results, the proposed method achieves a speedup ratio of 5.16×10^6^ times compared with the traditional time-sequential Monte Carlo simulation based on PSD-BPA, representing an acceleration of more than six orders of magnitude. Even under an ideal parallel computing condition with 1000-core parallelism and zero communication overhead, the total running time of the traditional method can be reduced to approximately 619.2 seconds, which still yields a speedup ratio of 5160, an improvement of three orders of magnitude.Furthermore, although the single inference time of the proposed DDPG-Attention (0.120 s) increases by approximately 0.08 s compared with MLP (0.041 s), roughly three times that of MLP, the previous accuracy comparisons reveal significant improvements in prediction performance. Under the statistical framework of repeated experiments with multiple random seeds, the MAE of DDPG-Attention is 0.0315 ± 0.0009, approximately 20.9% lower than the 0.0398 ± 0.0012 of MLP; its P95AE reaches 0.0772 ± 0.0028, which is around 21.3% lower than MLP’s 0.0981 ± 0.0035.For power system security assessment, the losses caused by misjudgment and missed judgment of high-risk operating conditions far outweigh the millisecond-level computational delay. This well-balanced trade-off between accuracy and effciency possesses clear practical engineering value.

7 Conclusion

This paper constructs a rapid power balance risk assessment framework under extreme weather conditions by integrating meteorological sensitivity models, time-varying component failure probabilities, DVAE-based coupled scenario generation, time-series labels from PSD-BPA, and an attention-enhanced DDPG estimator. The revised approach explicitly defines the state space, continuous action space, state transition rules, reward function, feature token attention weights, supervised learning baselines, independent test metrics, and timing schemes. The main conclusions are summarized as follows:

(1) The attention mechanism operates on multiple feature tokens instead of a single scalar score. The derived attention matrix is normalized row-wisely, enabling the Actor network to adaptively reweight meteorological data, power source information, grid parameters, load profiles, and risk features from the previous time step along the evolution of the 72-hour scenarios. Under the statistical framework of ten independent runs with random seeds ranging from 2021 to 2030, the MAE, RMSE, *R*^2^ and P95AE of DDPG-Attention on the independent test set are 0.0315 ± 0.0009, 0.0394 ± 0.0012, 0.952 ± 0.003 and 0.0772 ± 0.0028, respectively. Compared with vanilla DDPG (0.0385 ± 0.0011, 0.0482 ± 0.0015, 0.929 ± 0.004, 0.0951 ± 0.0032), its MAE and RMSE are reduced by approximately 18.2% and 18.3%, *R*^2^ is improved by around 2.5%, and P95AE declines by roughly 18.8%. In comparison with the supervised learning baseline MLP (0.0398 ± 0.0012, 0.0500 ± 0.0016, 0.923 ± 0.004, 0.0981 ± 0.0035), MAE, RMSE and P95AE decrease by about 20.9%, 21.2% and 21.3%, while *R*^2^ increases by approximately 3.1%, The Wilcoxon signed-rank test confirms that these improvements are statistically significant (p < 0.01), demonstrating that the performance gains stem from the attention mechanism rather than random training variance. Meanwhile, it exhibits a faster reward convergence rate. It should be noted that the reported accuracy metrics are based on open-loop evaluation using ground-truth previous-step risk labels. In practical closed-loop deployment, the MAE moderately increases to 0.0382 due to error accumulation, yet the inference speed remains within 0.12 s, confirming the method’s engineering applicability.

**Reviewer#1, Concern #4 The manuscript compares a sequential MDP formulation (state includes risk features from the previous time step, per Section 5.1) against a feedforward MLP baseline trained by direct regression. The text does not specify whether the MLP receives the same previous-step risk features as the RL agents during evaluation, nor whether the RL agents are evaluated in open-loop (using ground-truth previous-step values) or closed-loop (using their own prior predictions) rollout on the independent test set. This distinction matters for a fair comparison, since closed-loop evaluation typically compounds error over the 72-hour horizon while open-loop evaluation does not.**

**Author response:** 非常感谢您指出我们在评估协议描述方面的疏漏。您提出的关于开环（open-loop）与闭环（closed-loop）评估的问题非常专业且关键，直接关系到MLP基线与本方法之间比较的公平性与可信度。我们对此深表歉意，未能在原稿中清晰阐明这一重要细节。针对您的意见，我们进行了以下澄清和修改：

在原稿中，我们实际上采用了一种公平且严谨的评估协议，但未能在文字中清晰表述。现澄清如下：

（1）评估模式：开环评估（Open-loop Evaluation）

在独立测试集上，所有模型——包括RL智能体（DDPG-Attention、DDPG、SAC、PPO、DQN）和监督学习MLP基线——均采用开环评估方式。具体而言，在每个时间步 $t$，状态向量中的“前一时刻风险特征” $R_{t-1}$ 使用的是由PSD-BPA计算得出的真实标签值，而非模型自身的先前预测。

（2）公平比较的前提

采用开环评估确保了比较的公平性：

所有模型在相同条件下进行比较——都使用相同的真实 $R_{t-1}$ 值；

比较的仅仅是模型在当前系统状态下的“单步映射能力”，而非模型处理自身误差累积的能力；

MLP与RL智能体在评估时处于完全对等的地位。

（3）开环评估的合理性依据

本研究的核心目标是“快速将当前系统状态映射为风险指标”，相当于用训练好的模型替代耗时的PSD-BPA仿真过程；

开环评估直接度量这一映射关系的准确性，与研究目标一致；

由于PSD-BPA能够为历史数据提供真实标签，在评估阶段使用这些真实值是完全可行且合理的。

（4）训练与部署的区分

需要强调的是：

训练阶段：RL智能体学习过程中，状态转移依赖环境反馈，$R_{t-1}$ 来自历史真实数据（PSD-BPA标签）；

评估阶段（独立测试集）：采用开环评估，$R_{t-1}$ 使用真实标签值；

实际部署阶段（在线推理）：采用闭环自回归推理，$R_{t-1}$ 使用模型自身的预测值。

（5）开环/闭环性能差异的补充说明

闭环推理的误差累积效应是客观存在的。为量化这一影响，我们补充了对比实验：在相同测试集上分别以开环和闭环方式评估DDPG-Attention。结果表明，闭环推理的MAE为0.0382，较开环（0.0315）升高约21%。尽管存在性能下降，闭环推理0.120 s的推理速度和0.0382的MAE仍能满足实际工程对时效性和精度的基本需求。该结果已在修订稿第6.4.1节末尾补充说明。

**Author action:**

（1）第5.1节（MDP建模）末尾新增评估协议说明

Evaluation Protocol: During the evaluation phase on the independent test set, all models—including the RL agents and the MLP baseline—are evaluated in an open-loop manner. Specifically, the ‘previous-step risk features’ $\text{R}_{\text{t}\text{-1}}^{\text{label}}$ provided in the state vector are the ground-truth labels calculated by PSD-BPA, rather than the models’ own prior predictions. This protocol ensures a fair comparison by evaluating each model’s capability to map the current system state to risk indicators without the confounding effect of temporal error accumulation. In actual online deployment, however, closed-loop autoregressive inference is adopted, where $\text{R}_{\text{t-}\text{1}}$ is recursively fed from the model’s own predictions.

（2）第5.3节（MLP基线）中明确说明输入一致性

To prevent temporal information leakage, the dataset must be split into mutually exclusive training, validation and test sets on the basis of complete 72-hour scenarios rather than individual hourly samples. The exact number of scenarios in each subset and the random seed are reported for reproducibility. The MLP baseline is trained via direct regression with the same normalized input features as the RL agents, including the ground-truth risk features from the previous time step during training. During evaluation, the MLP also receives the same open-loop ground-truth${\text{ }\text{R}}_{\text{t}\text{-1}}^{\text{label}}$as the RL agents, ensuring an equitable comparison.

（3）第6.4.1节（精度比较）中补充说明

As can be observed from Table 11, statistical results from repeated experiments with multiple random seeds demonstrate that the proposed DDPG-Attention outperforms all baseline algorithms in terms of the mean values of the four evaluation metrics. Specifically, regarding overall prediction accuracy, the MAE and RMSE of DDPG-Attention are 0.0315 ± 0.0009 and 0.0394 ± 0.0012, respectively. Compared with the best-performing baseline DDPG (0.0385 ± 0.0011 and 0.0482 ± 0.0015), these metrics are reduced by approximately 18.2% and 18.3%, while they decrease by around 20.9% and 21.2% relative to the supervised learning model MLP (0.0398 ± 0.0012 and 0.0500 ± 0.0016). These results reveal that the embedded attention mechanism can effectively capture critical spatio-temporal features strongly correlated with power balance risks, while the reinforcement learning framework further strengthens the model’s capability to characterize the temporal dependencies of risk evolution. It is worth noting that the accuracy comparison in Table 11 is based on open-loop evaluation for all models, where $\text{R}_{\text{t}\text{-1}}$ is the ground-truth PSD-BPA label. This protocol is adopted to ensure fair comparison of single-step mapping capability. For practical deployment, the model operates in a closed-loop autoregressive manner; the resulting performance degradation is quantified in Section 6.4.3, and the inference speed remains within the sub-second range.

（4）新增第6.4.3节（开环与闭环性能对比）

**6.4.3 Open-loop vs. Closed-loop Performance Analysis**

To quantify the impact of temporal error accumulation in closed-loop inference, we additionally evaluate DDPG-Attention in both open-loop and closed-loop modes on the same independent test set. In closed-loop mode, the state at time step *t* uses the model’s own prediction from time step $\text{t}\text{-1}$ as $\text{R}_{\text{t}\text{-1}}$. The results are shown in Table 13.

**Table 13:** Comparison of relevant data under open-loop and closed-loop modes

| **Evaluation Mode** | **MAE** | **RMSE** | **R²** | **P95AE** |
| --- | --- | --- | --- | --- |
| Open-loop | 0.0315 ± 0.0009 | 0.0394 ± 0.0012 | 0.952 ± 0.003 | 0.0772 ± 0.0028 |
| Closed-loop | 0.0382 ± 0.0015 | 0.0476 ± 0.0020 | 0.926 ± 0.005 | 0.0935 ± 0.0038 |

The closed-loop MAE is approximately 21% higher than the open-loop MAE, attributable to the compounding effect of prediction errors over the 72-hour horizon. Nevertheless, the closed-loop MAE of 0.0382 remains comparable to the open-loop performance of vanilla DDPG (0.0385), and the inference speed of 0.120s comfortably satisfies the sub-second requirement for online deployment. This demonstrates that the proposed method maintains practical utility even under the more challenging closed-loop inference condition.

（5）第7节（结论）中补充说明

(1) The attention mechanism operates on multiple feature tokens instead of a single scalar score. The derived attention matrix is normalized row-wisely, enabling the Actor network to adaptively reweight meteorological data, power source information, grid parameters, load profiles, and risk features from the previous time step along the evolution of the 72-hour scenarios. Under the statistical framework of ten independent runs with random seeds ranging from 2021 to 2030, the MAE, RMSE, *R*^2^ and P95AE of DDPG-Attention on the independent test set are 0.0315 ± 0.0009, 0.0394 ± 0.0012, 0.952 ± 0.003 and 0.0772 ± 0.0028, respectively. Compared with vanilla DDPG (0.0385 ± 0.0011, 0.0482 ± 0.0015, 0.929 ± 0.004, 0.0951 ± 0.0032), its MAE and RMSE are reduced by approximately 18.2% and 18.3%, *R*^2^ is improved by around 2.5%, and P95AE declines by roughly 18.8%. In comparison with the supervised learning baseline MLP (0.0398 ± 0.0012, 0.0500 ± 0.0016, 0.923 ± 0.004, 0.0981 ± 0.0035), MAE, RMSE and P95AE decrease by about 20.9%, 21.2% and 21.3%, while *R*^2^ increases by approximately 3.1%, The Wilcoxon signed-rank test confirms that these improvements are statistically significant (p < 0.01), demonstrating that the performance gains stem from the attention mechanism rather than random training variance. Meanwhile, it exhibits a faster reward convergence rate. It should be noted that the reported accuracy metrics are based on open-loop evaluation using ground-truth previous-step risk labels. In practical closed-loop deployment, the MAE moderately increases to 0.0382 due to error accumulation, yet the inference speed remains within 0.12 s, confirming the method’s engineering applicability.

**Reviewer#1, Concern #5 The authors are encouraged to discuss the following references, briefly acknowledging what these works accomplished and the results they achieved, so as to better position the present study within the existing literature.**

**Author response:** 非常感谢您建议我们讨论两篇参考文献，以更好地将本研究定位在现有文献中。我们认真阅读了您推荐的文献，并按照您的建议在引言部分增加了对这两项工作的简要讨论。

文献[1]（Feng et al.）针对高比例可再生能源接入导致的系统惯量下降问题，提出了一种虚拟电厂（VPP）聚合模型和市场机制，使分布式能源能够参与惯性和一次频率响应。该方法在IEEE 30节点和118节点系统的测试中，对频率最低点和准稳态频率的拟合平均绝对百分比误差不超过0.03%，系统总成本降低约40%，净利润增加约30%。该研究与我们关注的问题具有场景关联性——都聚焦于高比例可再生能源接入带来的系统稳定性挑战，但文献[1]侧重于频率稳定与市场机制设计，而本文关注功率平衡风险。

文献[2]（Meng et al.）针对双侧供电铁路系统中车辆运行点频繁变化导致的稳定性问题，提出了一种可解释的残差前馈神经网络（ResFNN）结合SHAP方法，用于高效、准确地辨识列车变流器的“黑箱”阻抗模型，评估系统在低频频段和高频频段的稳定性。该研究在方法论层面与本文具有关联性——都利用了神经网络来处理传统方法难以解决的复杂系统建模与辨识问题。

在正文中，我们已将上述讨论插入到引言第三段末尾（即“现有研究不足”与“本文方法概述”之间的过渡位置），并以此引出本文的研究动机和创新点。具体修改内容如下：

**Author action:**

在引言第三段后加入相关内容：

1 Introduction

With the in depth advancement of the “dual carbon” goals, the construction of new power systems has entered a critical stage, and the share of renewable energy installations, represented by wind power and photovoltaics (PV), continues to increase^[1-2]^. However, the strong intermittency and volatility of renewable energy output make the system operation significantly more dependent on meteorological conditions. Unlike highly destructive extreme weather events such as typhoons and thunderstorms, persistent stagnant weather does not directly damage the physical structure of the power grid, but its long duration and wide impact range can easily lead to renewable energy being in a state of low or even zero output for an extended period, thereby triggering long term power supply demand imbalance risks in the power system. Events such as high temperature droughts and multiple days without wind or sunlight that have occurred in many regions worldwide have fully demonstrated that weather factors are becoming an important unknown risk source threatening the security of power supply in new power systems^[3-4]^. Therefore, studying the supply demand imbalance risk of power systems under extreme weather and developing fast and accurate risk assessment methods are of significant theoretical importance and engineering value.

Currently, domestic and international scholars have conducted extensive research on power system risk assessment under extreme weather. In terms of source load characteristic modeling, references^[5-6]^ proposed renewable energy station output prediction methods considering factors such as wind speed, temperature, humidity, and solar irradiance. Reference[7] separated climate sensitive load from total load by analyzing the spatiotemporal correlation between climate factors and power load. Reference [8] studied the impact of high temperature and drought weather on the output of hydropower and thermal power, and proposed elasticity indicators at the power source level. In the field of system resilience assessment, existing studies have proposed static resilience indicators based on resource adequacy and component redundancy^[9-10]^, as well as dynamic resilience indicators established according to the recovery process during and after extreme events. Regarding scenario generation, reference [11] proposed a weather scenario construction method based on extreme value theory, but it lacked specific scenario modeling and case study validation. References [12-13] explored the emergency control strategies of power systems and the optimal configuration of distributed generation and energy storage devices under different scenarios.

However, existing research still has obvious limitations in coping with supply demand imbalance risks of power systems under extreme weather. On the one hand, most studies tend to analyze sudden extreme weather events and persistent stagnant weather separately, failing to uniformly reveal the differentiated impact mechanisms of the two types of extreme weather from the three aspects of supply, transmission, and demand. On the other hand, at the uncertainty modeling level, source side output fluctuations, grid side equipment failures, and load side climate sensitive responses are often treated in isolation, and a comprehensive quantification of their coupled uncertainties is lacking. In particular, the time varying characteristics of equipment failure probability, which are jointly affected by service life, environmental conditions, and operating status, are insufficiently considered in existing risk assessment models. More critically, although traditional time series production simulation or Monte Carlo methods can obtain relatively accurate risk results, their huge computational overhead makes it difficult to meet the timeliness requirements for rapid risk identification during the evolution of extreme weather. Moreover, existing resilience assessment studies mostly focus on short term emergency power supply after the destruction of grid topology by extreme weather, while the exploration of rapid assessment methods for global and persistent supply demand imbalance risks remains insufficient. The above deficiencies indicate that there is an urgent need to develop an intelligent method that can efficiently handle high dimensional state spaces, adaptively focus on key risk features, and combine offline training with online rapid assessment. Recently, data-driven methods have also been explored to address complex challenges in power and energy systems. Feng et al. [41] proposed a virtual power plant aggregation model and market mechanism that enables distributed energy resources to provide inertia and primary frequency response, achieving high modeling accuracy with mean absolute percentage errors below 0.03% in frequency response fitting and approximately 40% reduction in system operation costs. Separately, Meng et al. [42] developed an interpretable neural network for impedance identification in double-sided feeding railway systems, combining residual feedforward neural networks with SHAP analysis to effectively assess stability under variable operating conditions. While these studies demonstrate the potential of learning-based methods for system modeling and stability analysis, they do not directly address the problem of rapid, sequential risk assessment for power balance under extreme weather events—particularly the coupled uncertainties from source, grid, and load sides.

41 C. Feng, Huang Z, Lin J, et al. Aggregation model and market mechanism for virtual power plant participation in inertia and primary frequency response[J]. IEEE Transactions on Power Systems, 2025.
42 Meng X, Hu G, Liu Z, et al. Neural network-based impedance identification and stability analysis for double-sided feeding railway systems[J]. IEEE Transactions on Transportation Electrification, 2024, 11(1): 4429-4442.
