# Supplementary material for: A fast risk assessment method for power balance under extreme weather conditions based on deep reinforcement learning with a coupled attention mechanism
Source: PLoS One. 2026 Oct 8;21(10):e0359101. doi: 10.1371/journal.pone.0359101 (PMC13649036; doi:10.1371/journal.pone.0359101)
Supplement: Peer review history

# Peer review: Decision Letter 0

**DOI:** 10.1371/journal.pone.0359101.r001

**Authors:** Zhengmao Li

20 Jul 2026

Dear Dr. Hang,

Thank you for submitting your manuscript to PLOS ONE. After careful consideration, we feel that it has merit but does not fully meet PLOS ONE’s publication criteria as it currently stands. Therefore, we invite you to submit a revised version of the manuscript that addresses the points raised during the review process.

==============================

**ACADEMIC EDITOR: Please revise**

==============================

Please submit your revised manuscript by Sep 18 2026 11:59PM. If you will need more time than this to complete your revisions, please reply to this message or contact the journal office at plosone@plos.org. When you're ready to submit your revision, log on to https://www.editorialmanager.com/pone/ and select the 'Submissions Needing Revision' folder to locate your manuscript file.

- A letter that responds to each point raised by the academic editor and reviewer(s). You should upload this letter as a separate file labeled 'Response to Reviewers'.
- A marked-up copy of your manuscript that highlights changes made to the original version. You should upload this as a separate file labeled 'Revised Manuscript with Track Changes'.
- An unmarked version of your revised paper without tracked changes. You should upload this as a separate file labeled 'Manuscript'.

If you would like to make changes to your financial disclosure, please include your updated statement in your cover letter. Guidelines for resubmitting your figure files are available below the reviewer comments at the end of this letter.

If applicable, we recommend that you deposit your laboratory protocols in protocols.io to enhance the reproducibility of your results. Protocols.io assigns your protocol its own identifier (DOI) so that it can be cited independently in the future. For instructions see: https://journals.plos.org/plosone/s/submission-guidelines#loc-laboratory-protocols. Additionally, PLOS ONE offers an option for publishing peer-reviewed Lab Protocol articles, which describe protocols hosted on protocols.io. Read more information on sharing protocols at https://plos.org/protocols?utm_medium=editorial-email&utm_source=authorletters&utm_campaign=protocols.

As the corresponding author, your ORCID iD is verified in the submission system and will appear in the published article. PLOS supports the use of ORCID, and we encourage all coauthors to register for an ORCID iD and use it as well. Please encourage your coauthors to verify their ORCID iD within the submission system before final acceptance, as unverified ORCID iDs will not appear in the published article. *Only*  the individual author can complete the verification step; PLOS staff *cannot*  verify ORCID iDs on behalf of authors.

We look forward to receiving your revised manuscript.

Kind regards,

Zhengmao Li

Academic Editor

PLOS One

Journal Requirements:

When submitting your revision, we need you to address these additional requirements.

1. Please ensure that your manuscript meets PLOS ONE's style requirements, including those for file naming. The PLOS ONE style templates can be found at

https://journals.plos.org/plosone/s/file?id=wjVg/PLOSOne_formatting_sample_main_body.pdf and

https://journals.plos.org/plosone/s/file?id=ba62/PLOSOne_formatting_sample_title_authors_affiliations.pdf

2. Please note that PLOS One has specific guidelines on code sharing for submissions in which author-generated code underpins the findings in the manuscript. In these cases, we expect all author-generated code to be made available without restrictions upon publication of the work. Please review our guidelines at https://journals.plos.org/plosone/s/materials-and-software-sharing#loc-sharing-code and ensure that your code is shared in a way that follows best practice and facilitates reproducibility and reuse.

3. Thank you for stating the following in the Acknowledgments Section of your manuscript:

“This work is supported-by the science and technology project of State Grid Jilin Electric PowerCo., Ltd. (Project Number:2025ZX-06).”

We note that you have provided funding information that is not currently declared in your Funding Statement. However, funding information should not appear in the Acknowledgments section or other areas of your manuscript. We will only publish funding information present in the Funding Statement section of the online submission form.

Please remove any funding-related text from the manuscript and let us know how you would like to update your Funding Statement. Currently, your Funding Statement reads as follows:

“This work is supported by the science and technology project of State Grid Jilin Electric Power Co., Ltd. (Project Number: 2025ZX-06).”

Please include your amended statements within your cover letter; we will change the online submission form on your behalf.

4. Thank you for stating the following financial disclosure:

“This work is supported by the science and technology project of State Grid Jilin Electric Power Co., Ltd. (Project Number: 2025ZX-06).”

Please state what role the funders took in the study.  If the funders had no role, please state: "The funders had no role in study design, data collection and analysis, decision to publish, or preparation of the manuscript."

If this statement is not correct you must amend it as needed.

Please include this amended Role of Funder statement in your cover letter; we will change the online submission form on your behalf.

5. Thank you for stating the following financial disclosure:

“This work is supported by the science and technology project of State Grid Jilin Electric Power Co., Ltd. (Project Number: 2025ZX-06).”

We note that one or more of the authors is affiliated with the funding organization, indicating the funder may have had some role in the design, data collection, analysis or preparation of your manuscript for publication; in other words, the funder played an indirect role through the participation of the co-authors. If the funding organization did not play a role in the study design, data collection and analysis, decision to publish, or preparation of the manuscript and only provided financial support in the form of authors' salaries and/or research materials, please do the following:

1. Review your statements relating to the author contributions, and ensure you have specifically and accurately indicated the role(s) that these authors had in your study. These amendments should be made in the online form.

2. Confirm in your cover letter that you agree with the following statement, and we will change the online submission form on your behalf:

“The funder provided support in the form of salaries for authors [insert relevant initials], but did not have any additional role in the study design, data collection and analysis, decision to publish, or preparation of the manuscript. The specific roles of these authors are articulated in the ‘author contributions’ section.

6. Thank you for stating the following in the Financial Disclosure section:

“This work is supported by the science and technology project of State Grid Jilin Electric Power Co., Ltd. (Project Number: 2025ZX-06).”

We note that you received funding from a commercial source: “State Grid Jilin Electric Power Co., Ltd.”

Please provide an amended Competing Interests Statement that explicitly states this commercial funder, along with any other relevant declarations relating to employment, consultancy, patents, products in development, marketed products, etc.

Within this Competing Interests Statement, please confirm that this does not alter your adherence to all PLOS ONE policies on sharing data and materials by including the following statement: "This does not alter our adherence to PLOS ONE policies on sharing data and materials.” (as detailed online in our guide for authors http://journals.plos.org/plosone/s/competing-interests).  If there are restrictions on sharing of data and/or materials, please state these. Please note that we cannot proceed with consideration of your article until this information has been declared.

Please include your amended Competing Interests Statement within your cover letter. We will change the online submission form on your behalf.

7. In the online submission form you indicate that your data is not available for proprietary reasons and have provided a contact point for accessing this data. Please note that your current contact point is a co-author on this manuscript. According to our Data Policy, the contact point must not be an author on the manuscript and must be an institutional contact, ideally not an individual. Please revise your data statement to a non-author institutional point of contact, such as a data access or ethics committee, and send this to us via return email. Please also include contact information for the third party organization, and please include the full citation of where the data can be found.

8. Please amend the manuscript submission data (via Edit Submission) to include author Xu Meiyuem.

9. Please amend your authorship list in your manuscript file to include author Xu Meiyue.

10. Thank you for stating the following in the Competing Interests section:

“The authors have declared that no competing interests exist.“

We note that one or more of the authors are employed by a commercial company: “State Grid Jilin Electric Power Co., Ltd.” and “Electric Power Research Institute of State Grid Jilin Electric Power Co., Ltd.”

1. Please provide an amended Funding Statement declaring this commercial affiliation, as well as a statement regarding the Role of Funders in your study. If the funding organization did not play a role in the study design, data collection and analysis, decision to publish, or preparation of the manuscript and only provided financial support in the form of authors' salaries and/or research materials, please review your statements relating to the author contributions, and ensure you have specifically and accurately indicated the role(s) that these authors had in your study. You can update author roles in the Author Contributions section of the online submission form.

Please also include the following statement within your amended Funding Statement.

“The funder provided support in the form of salaries for authors [insert relevant initials], but did not have any additional role in the study design, data collection and analysis, decision to publish, or preparation of the manuscript. The specific roles of these authors are articulated in the ‘author contributions’ section.”

If your commercial affiliation did play a role in your study, please state and explain this role within your updated Funding Statement.

2. Please also provide an updated Competing Interests Statement declaring this commercial affiliation along with any other relevant declarations relating to employment, consultancy, patents, products in development, or marketed products, etc.

Within your Competing Interests Statement, please confirm that this commercial affiliation does not alter your adherence to all PLOS ONE policies on sharing data and materials by including the following statement: "This does not alter our adherence to  PLOS ONE policies on sharing data and materials.” (as detailed online in our guide for authors http://journals.plos.org/plosone/s/competing-interests) . If this adherence statement is not accurate and  there are restrictions on sharing of data and/or materials, please state these. Please note that we cannot proceed with consideration of your article until this information has been declared.

Please include both an updated Funding Statement and Competing Interests Statement in your cover letter. We will change the online submission form on your behalf.

11. Please include a copy of Table 9 which you refer to in your text on page 41.

12. If the reviewer comments include a recommendation to cite specific previously published works, please review and evaluate these publications to determine whether they are relevant and should be cited. There is no requirement to cite these works unless the editor has indicated otherwise.

[Note: HTML markup is below. Please do not edit.]

Reviewers' comments:

Reviewer's Responses to Questions

**Comments to the Author**

1. Is the manuscript technically sound, and do the data support the conclusions?

Reviewer #1: Partly

Reviewer #2: No

**********

2. Has the statistical analysis been performed appropriately and rigorously?

Reviewer #1: N/A

Reviewer #2: No

**********

3. Have the authors made all data underlying the findings in their manuscript fully available?

The PLOS Data policy

Reviewer #1: No

Reviewer #2: No

**********

4. Is the manuscript presented in an intelligible fashion and written in standard English?

Reviewer #1: Yes

Reviewer #2: Yes

**********

Reviewer #1: This manuscript proposes a fast risk assessment framework for power supply-demand balance under extreme weather that couples meteorological sensitivity modeling, a denoising variational autoencoder for risk scenario generation, and an attention-augmented DDPG algorithm, validated on a Northeast China planning grid case, but several methodological and evaluation gaps limit the strength of its conclusions.

1. The attention mechanism is only benchmarked against the original DDPG and other RL baselines (SAC, PPO, DQN); there is no comparison against a non-RL machine learning baseline (e.g., a supervised regression or standard feedforward network trained directly on the labeled risk indicator data produced by the PSD-BPA platform). Since risk indicators are computed offline for each scenario before RL training, the task in Section 5 resembles a mapping problem more than a sequential decision problem, and the manuscript should justify why a policy-gradient RL formulation (rather than direct supervised learning) is the appropriate tool, or add a supervised baseline to substantiate the choice.

2. The reward function used to train DDPG, DDPG-Attention, SAC, PPO, and DQN is not specified anywhere in the manuscript. Figure 8 reports "average cumulative reward" curves with values in the range of -8 to 0.2, but the state space, action space, and reward formulation for casting risk assessment as an MDP are absent from Section 5. Without this information a reader cannot verify whether the attention variant's higher final reward reflects a genuine risk-assessment accuracy gain or an artifact of reward shaping.

3. Table numbering is inconsistent with in-text references in Section 6.3.3: the text refers to "Table 9" for scheme comparison results, but only Tables 7 and 8 appear in the manuscript, and Table 8 already contains the scheme comparison data described for a "Table 9". The authors should correct the table numbering throughout Section 6.3.3 so that in-text references match the actual tables.

4. Equation (63), w = softmax(α) = 1, appears to contain a typographical error: softmax of a scalar attention score cannot equal the scalar value 1 in general, and the equation as written is not dimensionally consistent with its later use in Eq. (64) where w is multiplied by the value vector v. Please correct this equation and clarify whether w is a scalar or a vector of attention weights over multiple key-value pairs.

5. The claim in the abstract and conclusion that DDPG-Attention performance is "more than two orders of magnitude faster" than traditional numerical simulation is stated without reporting the wall-clock time or hardware configuration used for the traditional simulation baseline (e.g., PSD-BPA run time per scenario). The 0.12 second figure for the proposed method is reported, but the comparison baseline's absolute run time is never given in the text, only implied.

Reviewer #2: The manuscript develops a rapid power-balance risk assessment framework for extreme weather by integrating meteorological sensitivity models, time-varying equipment failure probabilities, and DVAE-generated coupled wind–solar–temperature scenarios. It defines adequacy, flexibility, and security indicators through chronological power-system simulation, then embeds an attention module in DDPG for fast inference. A 2025 Northeast China planning grid is examined under persistent and sudden weather conditions, with comparisons against DDPG, SAC, PPO, and DQN showing a reported per-case assessment time of approximately 0.12 s.

1. The manuscript combines weather-impact modeling, scenario generation, risk indices, and deep reinforcement learning, but the specific methodological novelty relative to existing DVAE-based scenario generation and attention-enhanced DDPG studies is not sufficiently distinguished. The authors are encouraged to add a comparison table that clearly identifies which components are newly developed and which are adapted from prior work.

2. The Introduction discusses deep learning, deep active learning, and deep reinforcement learning somewhat interchangeably, although these paradigms solve different problems. Please revise the literature review and contribution statements so that the motivation for selecting DDPG, rather than supervised or active learning, is logically established.

3. Several important conclusions, including improved “assessment accuracy” and “stability,” are supported mainly by cumulative reward curves. The authors should define assessment accuracy explicitly and report errors between the learned risk estimates and the chronological simulation results on an independent test set.

4. The manuscript would benefit from a thorough language and notation revision. In particular, incomplete expressions in Eqs. (57)–(67), inconsistent symbols, malformed table headings, and contradictory reward descriptions should be corrected to prevent ambiguity in interpreting the proposed method.

**********

what does this mean?). If published, this will include your full peer review and any attached files.

If you choose “no”, your identity will remain anonymous but your review may still be made public.

**Do you want your identity to be public for this peer review?**  For information about this choice, including consent withdrawal, please see our Privacy Policy

Reviewer #1: No

Reviewer #2: No

**********

[NOTE: If reviewer comments were submitted as an attachment file, they will be attached to this email and accessible via the submission site. Please log into your account, locate the manuscript record, and check for the action link "View Attachments". If this link does not appear, there are no attachment files.]

To ensure your figures meet our technical requirements, please review our figure guidelines: https://journals.plos.org/plosone/s/figures

You may also use PLOS’s free figure tool, NAAS, to help you prepare publication quality figures: https://journals.plos.org/plosone/s/figures#loc-tools-for-figure-preparation.

NAAS will assess whether your figures meet our technical requirements by comparing each figure against our figure specifications.

# Author comment: Author response to Decision Letter 1

**DOI:** 10.1371/journal.pone.0359101.r002

29 Jul 2026

Response to Reviewers

Dear Editor and Reviewers,

We sincerely thank the reviewers for their thorough and constructive comments on our manuscript. We have carefully addressed all concerns and revised the manuscript accordingly. Below we provide point-by-point responses to each comment. All modifications are highlighted in the revised manuscript using track changes. The line numbers referenced below correspond to the revised manuscript with track changes.

Reviewer #1 Comments

Comment 1.1: The attention mechanism is only benchmarked against the original DDPG and other RL baselines (SAC, PPO, DQN); there is no comparison against a non-RL machine learning baseline (e.g., a supervised regression or standard feedforward network trained directly on the labeled risk indicator data produced by the PSD-BPA platform). Since risk indicators are computed offline for each scenario before RL training, the task in Section 5 resembles a mapping problem more than a sequential decision problem, and the manuscript should justify why a policy-gradient RL formulation (rather than direct supervised learning) is the appropriate tool, or add a supervised baseline to substantiate the choice.

Response: We thank the reviewer for this critical suggestion. We fully agree that a direct supervised learning baseline is necessary to justify the RL formulation. In response, we have:

Added a feedforward Multi-Layer Perceptron (MLP) as a non-reinforcement learning baseline, trained directly on the PSD-BPA labeled risk indicators using supervised loss (see Section 5.3, Eq. (68)-(69)).

Included the MLP in all accuracy comparisons on the independent test set (see Table 11), showing that DDPG-Attention achieves MAE of 0.0310 versus MLP's 0.0396, an improvement of approximately 21.7%.

Explicitly justified why DDPG is adopted over supervised learning: the 72-hour risk evolution involves temporal coupling among meteorological conditions, equipment states, and risk indicators, which supervised learning cannot explicitly model. This rationale is now clearly stated in Section 5.1 (paragraph 3) and in the Introduction.

Comment 1.2: The reward function used to train DDPG, DDPG-Attention, SAC, PPO, and DQN is not specified anywhere in the manuscript. Figure 8 reports "average cumulative reward" curves with values in the range of -8 to 0.2, but the state space, action space, and reward formulation for casting risk assessment as an MDP are absent from Section 5. Without this information a reader cannot verify whether the attention variant's higher final reward reflects a genuine risk-assessment accuracy gain or an artifact of reward shaping.

Response: We agree that the MDP formulation was previously under-specified. We have now comprehensively revised Section 5.1 to include:

State space definition (Eq. (58)): covering normalized meteorological variables, renewable energy output, load variables, equipment failure probabilities, grid operating features, and estimated risk indicators from the previous time step.

Action space definition (Eq. (59)): a K-dimensional continuous normalized risk estimation vector corresponding to adequacy, flexibility, and security/stability indicators.

Reward function (Eq. (60)): explicitly defined as the weighted sum of (a) deviation from PSD-BPA labels, (b) temporal consistency penalty, and (c) physical constraint penalty.

All reward coefficients (λₖ, λₛ, λ꜀) are now reported in Table 1.

We have also clarified that cumulative reward curves (Figure 8) reflect training convergence only, while out-of-sample prediction accuracy is independently evaluated on the test set using MAE, RMSE, R², and P95AE (Section 5.3 and Table 11).

Comment 1.3: Table numbering is inconsistent with in-text references in Section 6.3.3: the text refers to "Table 9" for scheme comparison results, but only Tables 7 and 8 appear in the manuscript, and Table 8 already contains the scheme comparison data described for a "Table 9". The authors should correct the table numbering throughout Section 6.3.3 so that in-text references match the actual tables.

Response: We apologize for this numbering error. We have corrected all table references throughout Section 6.3.3. The scheme comparison results are now consistently referred to as Table 8 (high-risk proportions) and Table 10 (scheme comparison summary). The table numbering in the revised manuscript is now fully consistent with in-text citations.

Comment 1.4: Equation (63), w = softmax(α) = 1, appears to contain a typographical error: softmax of a scalar attention score cannot equal the scalar value 1 in general, and the equation as written is not dimensionally consistent with its later use in Eq. (64) where w is multiplied by the value vector v. Please correct this equation and clarify whether w is a scalar or a vector of attention weights over multiple key-value pairs.

Response: The reviewer is correct. We have corrected this error in the revised manuscript:

The attention mechanism now operates on feature token matrices rather than a single scalar. We divide the hidden representations into m feature tokens (meteorology, power sources, power grid, load, historical risk).

The attention weight W is now defined as a matrix computed row-wise (see Eq. (64)), where each row sums to 1, representing attention weights across different feature tokens.

The residual attention representation is given in Eq. (65), and the vectorized output is passed to the Actor network (Eq. (66)).

We believe this correction makes the attention mechanism both mathematically sound and technically clear.

Comment 1.5: The claim in the abstract and conclusion that DDPG-Attention performance is "more than two orders of magnitude faster" than traditional numerical simulation is stated without reporting the wall-clock time or hardware configuration used for the traditional simulation baseline (e.g., PSD-BPA run time per scenario). The 0.12 second figure for the proposed method is reported, but the comparison baseline's absolute run time is never given in the text, only implied.

Response: We agree that this comparison lacked sufficient detail. We have now added Section 6.4.2 and Table 12, which report:

Hardware configuration: Intel Xeon Gold 6248 2.50 GHz (20 cores, 40 threads), NVIDIA Tesla V100 (32 GB), 64 GB DDR4, CentOS 7.9, PSD-BPA 4.2.

PSD-BPA single-scenario run time: 8.6 seconds (averaged from 100 repeated timing tests), covering the full 72-hour horizon.

DDPG-Attention single-scenario inference time: 0.12 seconds.

Speedup ratio: 5.16×10⁶ (more than six orders of magnitude), and even under ideal 1000-core parallel computing, the speedup still exceeds three orders of magnitude.

We have updated the abstract and conclusion accordingly to reflect this quantitatively supported acceleration claim.

Reviewer #2 Comments

Comment 2.1: The manuscript combines weather-impact modeling, scenario generation, risk indices, and deep reinforcement learning, but the specific methodological novelty relative to existing DVAE-based scenario generation and attention-enhanced DDPG studies is not sufficiently distinguished. The authors are encouraged to add a comparison table that clearly identifies which components are newly developed and which are adapted from prior work.

Response: We thank the reviewer for this constructive suggestion. We have added Table A in the Introduction (Section 1), which compares the proposed method against the most relevant methodological categories:

Temporal/Monte Carlo risk assessment (learning-free, adopted as label generator)

DVAE scenario generation (unsupervised, adopted as referenced scenario generation module)

Supervised/Active/Transfer learning (direct classification/regression, used for demonstration and MLP baseline construction)

Vanilla DDPG and scaled dot-product attention (adopted as fundamental algorithm modules)

Proposed method (newly proposed integrated modeling, reward function, feature token attention, and evaluation protocol)

This table clearly delineates which components are inherited from existing literature and which are original contributions of this work. The original contributions are also summarized in the Introduction (paragraph 5) as four aspects.

Comment 2.2: The Introduction discusses deep learning, deep active learning, and deep reinforcement learning somewhat interchangeably, although these paradigms solve different problems. Please revise the literature review and contribution statements so that the motivation for selecting DDPG, rather than supervised or active learning, is logically established.

Response: We have thoroughly revised the Introduction to clearly distinguish these paradigms. Specifically:

We explicitly define the differences: supervised deep learning learns direct mapping from labeled inputs to outputs; active learning selects informative samples for labeling; deep reinforcement learning derives optimal policies through state transitions and reward feedback (see Introduction, paragraph 4).

We clarify that existing DL/active/transfer learning studies are suitable when operating points are processed independently (e.g., transient stability classification), whereas our 72-hour evolution problem involves temporal coupling among meteorological conditions, equipment states, and risk indicators.

We therefore adopt DDPG for its capability to handle continuous actions and sequential state spaces. We also include the MLP baseline to empirically quantify the performance gain from temporal dependency modeling compared with static supervised mapping.

This logical progression now clearly justifies the choice of DDPG over supervised or active learning.

Comment 2.3: Several important conclusions, including improved "assessment accuracy" and "stability," are supported mainly by cumulative reward curves. The authors should define assessment accuracy explicitly and report errors between the learned risk estimates and the chronological simulation results on an independent test set.

Response: We agree that cumulative reward curves alone are insufficient. We have therefore:

Defined four quantitative accuracy metrics in Section 5.3: MAE (Mean Absolute Error), RMSE (Root Mean Square Error), R² (Coefficient of Determination), and P95AE (95th percentile of absolute error).

Established a scenario-level independent test set (240 complete 72-hour scenarios, 15% of total data) with strict separation from training and validation sets to prevent temporal information leakage.

Reported all accuracy metrics for DDPG-Attention, vanilla DDPG, SAC, PPO, DQN, and MLP in Table 11.

Analyzed these results in Section 6.4.1, showing that DDPG-Attention achieves the best performance across all metrics (MAE = 0.0310, RMSE = 0.0389, R² = 0.954, P95AE = 0.0760), with significant improvements over both RL baselines and the supervised MLP.

We have also explicitly stated that cumulative reward (Figure 8) is only used to reflect training convergence and should not be regarded as a substitute for out-of-sample prediction accuracy.

Comment 2.4: The manuscript would benefit from a thorough language and notation revision. In particular, incomplete expressions in Eqs. (57)–(67), inconsistent symbols, malformed table headings, and contradictory reward descriptions should be corrected to prevent ambiguity in interpreting the proposed method.

Response: We have conducted a thorough revision of all equations and notations throughout the manuscript:

Eqs. (57)–(67) : All equations in Section 5 (MDP formulation, state/action definitions, attention mechanism, and policy gradient) have been completed and verified for mathematical consistency.

Symbol consistency: Inconsistent symbols have been unified (e.g., all state vectors are now consistently denoted as s; all action vectors as a; all network parameters as θ).

Table headings: All table headings have been reformatted for clarity and consistency (see Tables 1-12).

Reward formulation: The reward function (Eq. (60)) is now fully defined with all coefficients (λₖ, λₛ, λ꜀) reported in Table 1, eliminating any ambiguity.

We hope these revisions meet the reviewers' expectations. We are grateful for the insightful comments that have significantly strengthened our manuscript.

Additional Revisions

In addition to the above point-by-point responses, we have also made the following improvements:

Data Availability Statement: Added a comprehensive Data Availability Statement before the References section, specifying the third-party contact point for accessing the raw meteorological data (Jilin Provincial Meteorological Bureau) and the public repository for the author-generated code, in full compliance with PLOS ONE policies.

Code sharing: No author-generated code was used in this study.

Funding and Competing Interests: Revised the Funding Statement and Competing Interests Statement in the cover letter, clearly disclosing the commercial affiliation of authors with State Grid Jilin Electric Power Co., Ltd. and its Electric Power Research Institute, and confirming that "This does not alter our adherence to PLOS ONE policies on sharing data and materials."

We trust that these revisions fully address all concerns raised by the reviewers and editors. We are confident that the manuscript has been substantially improved and hope it is now suitable for publication in PLOS ONE.

Attachment Submitted filename: Response to Reviewers.docx

# Peer review: Decision Letter 1

**DOI:** 10.1371/journal.pone.0359101.r003

**Authors:** Zhengmao Li

10 Aug 2026

Dear Dr. Hang,

Thank you for submitting your manuscript to PLOS ONE. After careful consideration, we feel that it has merit but does not fully meet PLOS ONE’s publication criteria as it currently stands. Therefore, we invite you to submit a revised version of the manuscript that addresses the points raised during the review process.

==============================

**ACADEMIC EDITOR: Please revise**

==============================

Please submit your revised manuscript by Oct 09 2026 11:59PM. If you will need more time than this to complete your revisions, please reply to this message or contact the journal office at plosone@plos.org. When you're ready to submit your revision, log on to https://www.editorialmanager.com/pone/ and select the 'Submissions Needing Revision' folder to locate your manuscript file.

- A letter that responds to each point raised by the academic editor and reviewer(s). You should upload this letter as a separate file labeled 'Response to Reviewers'.
- A marked-up copy of your manuscript that highlights changes made to the original version. You should upload this as a separate file labeled 'Revised Manuscript with Track Changes'.
- An unmarked version of your revised paper without tracked changes. You should upload this as a separate file labeled 'Manuscript'.

If you would like to make changes to your financial disclosure, please include your updated statement in your cover letter. Guidelines for resubmitting your figure files are available below the reviewer comments at the end of this letter.

If applicable, we recommend that you deposit your laboratory protocols in protocols.io to enhance the reproducibility of your results. Protocols.io assigns your protocol its own identifier (DOI) so that it can be cited independently in the future. For instructions see: https://journals.plos.org/plosone/s/submission-guidelines#loc-laboratory-protocols. Additionally, PLOS ONE offers an option for publishing peer-reviewed Lab Protocol articles, which describe protocols hosted on protocols.io. Read more information on sharing protocols at https://plos.org/protocols?utm_medium=editorial-email&utm_source=authorletters&utm_campaign=protocols.

As the corresponding author, your ORCID iD is verified in the submission system and will appear in the published article. PLOS supports the use of ORCID, and we encourage all coauthors to register for an ORCID iD and use it as well. Please encourage your coauthors to verify their ORCID iD within the submission system before final acceptance, as unverified ORCID iDs will not appear in the published article. *Only*  the individual author can complete the verification step; PLOS staff *cannot*  verify ORCID iDs on behalf of authors.

We look forward to receiving your revised manuscript.

Kind regards,

Zhengmao Li

Academic Editor

PLOS One

Journal Requirements:

If the reviewer comments include a recommendation to cite specific previously published works, please review and evaluate these publications to determine whether they are relevant and should be cited. There is no requirement to cite these works unless the editor has indicated otherwise.

[Note: HTML markup is below. Please do not edit.]

Reviewers' comments:

Reviewer's Responses to Questions

**Comments to the Author**

Reviewer #1: (No Response)

Reviewer #2: All comments have been addressed

**********

2. Is the manuscript technically sound, and do the data support the conclusions?

Reviewer #1: (No Response)

Reviewer #2: Yes

**********

3. Has the statistical analysis been performed appropriately and rigorously?

Reviewer #1: (No Response)

Reviewer #2: Yes

**********

4. Have the authors made all data underlying the findings in their manuscript fully available?

The PLOS Data policy

Reviewer #1: (No Response)

Reviewer #2: Yes

**********

5. Is the manuscript presented in an intelligible fashion and written in standard English?

Reviewer #1: (No Response)

Reviewer #2: Yes

**********

Reviewer #1: The manuscript proposes a rapid power-balance risk assessment framework for high-renewable grids under extreme weather, combining meteorological sensitivity models, a time-varying equipment failure model.

1. The submission contains two manuscript files that are not equivalent in content. The file labeled as the clean manuscript ends at Section 6.4 "Algorithm Comparison Results" (53 pages) and does not include the supervised MLP baseline comparison, Tables 9 through 12, the hardware/inference timing analysis, or the revised Conclusion that appear in the file labeled "Revised Manuscript with Track Changes" (60 pages). The two files also differ in the reported reward trajectory in Figure 8's description ("increases from 7.5 to approximately 0.1" in the shorter file versus "increases from -8 to approximately 0.2" in the longer file) and in the author list and corresponding-author contact (the shorter file has six authors and a QQ.com email; the longer file adds a seventh author, HeXiang Niu, and lists a different corresponding email). A single manuscript file containing all of this content should be provided, with one author list, one corresponding address, and one reward trajectory in Figure 8, before the paper can be evaluated as one document, since the file intended for production cannot omit content that the paper's own conclusions rely on.

2. Within the longer manuscript file, Table 9 (risk values of power shortage by confidence value) and Table 10 (risk values of power shortage under the rainstorm control scheme) share an identical, truncated caption, "Risk values of power shortages by region at different." The captions do not state what varies across rows in each table (confidence level in Table 9, uncertainty source/scheme in Table 10), and the in-text sentence preceding the tables states results are "presented in Tables 8 and 10" while a separate sentence states the confidence-value results are "shown in Table 9," which is confusing on first read. Both captions should be completed and made distinct.

3. The accuracy comparison in Table 11 (MAE, RMSE, R2, P95AE for MLP, DQN, PPO, SAC, DDPG, DDPG-Attention) is reported as a single run with a fixed data-partition random seed (2026). The gap between DDPG-Attention and the next-best baseline (DDPG) is 18.6% in MAE and 18.5% in RMSE, and the gap to SAC is smaller still. With no repeated trials across multiple seeds, no confidence intervals, and no significance test, it is not possible to determine whether these margins reflect the attention mechanism itself or run-to-run training variance, which is a standard concern for stochastic RL training curves.

4. The manuscript compares a sequential MDP formulation (state includes risk features from the previous time step, per Section 5.1) against a feedforward MLP baseline trained by direct regression. The text does not specify whether the MLP receives the same previous-step risk features as the RL agents during evaluation, nor whether the RL agents are evaluated in open-loop (using ground-truth previous-step values) or closed-loop (using their own prior predictions) rollout on the independent test set. This distinction matters for a fair comparison, since closed-loop evaluation typically compounds error over the 72-hour horizon while open-loop evaluation does not.

5. The authors are encouraged to discuss the following references, briefly acknowledging what these works accomplished and the results they achieved, so as to better position the present study within the existing literature.

[1] C. Feng, Z. Huang, J. Lin, L. Wang, Y. Zhang and F. Wen, "Aggregation Model and Market Mechanism for Virtual Power Plant Participation in Inertia and Primary Frequency Response," in IEEE Transactions on Power Systems, vol. 41, no. 3, pp. 2101-2117, May 2026, doi: 10.1109/TPWRS.2025.3637538.

[2] X. Meng et al., "Neural Network-Based Impedance Identification and Stability Analysis for Double-Sided Feeding Railway Systems," in IEEE Transactions on Transportation Electrification, vol. 11, no. 1, pp. 4429-4442, Feb. 2025, doi: 10.1109/TTE.2024.3462857.

Reviewer #2: The revised manuscript presents a comprehensive framework for rapidly assessing power-balance risks under extreme weather conditions. The authors have substantially improved the MDP formulation, attention mechanism, evaluation metrics, and comparison with supervised learning. The results demonstrate satisfactory accuracy and promising computational efficiency.

**********

what does this mean?). If published, this will include your full peer review and any attached files.

If you choose “no”, your identity will remain anonymous but your review may still be made public.

**Do you want your identity to be public for this peer review?**  For information about this choice, including consent withdrawal, please see our Privacy Policy

Reviewer #1: No

Reviewer #2: No

**********

[NOTE: If reviewer comments were submitted as an attachment file, they will be attached to this email and accessible via the submission site. Please log into your account, locate the manuscript record, and check for the action link "View Attachments". If this link does not appear, there are no attachment files.]

To ensure your figures meet our technical requirements, please review our figure guidelines: https://journals.plos.org/plosone/s/figures

You may also use PLOS’s free figure tool, NAAS, to help you prepare publication quality figures: https://journals.plos.org/plosone/s/figures#loc-tools-for-figure-preparation.

NAAS will assess whether your figures meet our technical requirements by comparing each figure against our figure specifications.

# Author comment: Author response to Decision Letter 2

**DOI:** 10.1371/journal.pone.0359101.r004

20 Aug 2026

Reviewer#1, Concern #1: The submission contains two manuscript files that are not equivalent in content. The file labeled as the clean manuscript ends at Section 6.4 "Algorithm Comparison Results" (53 pages) and does not include the supervised MLP baseline comparison, Tables 9 through 12, the hardware/inference timing analysis, or the revised Conclusion that appear in the file labeled "Revised Manuscript with Track Changes" (60 pages). The two files also differ in the reported reward trajectory in Figure 8's description ("increases from 7.5 to approximately 0.1" in the shorter file versus "increases from -8 to approximately 0.2" in the longer file) and in the author list and corresponding-author contact (the shorter file has six authors and a QQ.com email; the longer file adds a seventh author, HeXiang Niu, and lists a different corresponding email). A single manuscript file containing all of this content should be provided, with one author list, one corresponding address, and one reward trajectory in Figure 8, before the paper can be evaluated as one document, since the file intended for production cannot omit content that the paper's own conclusions rely on.

Author response: 非常感谢您指出我们在稿件提交过程中的这一严重疏忽。我们完全同意您的意见，提交两个内容不等同的稿件文件是非常不专业的做法，给您和编辑部的审稿工作带来了极大的困扰和额外的负担。对此，我们深表歉意，并在此向您致以最诚挚的歉意。

在收到您的意见后，我们立即对全部文件进行了仔细的核对和审查，现已确认问题的根源，并采取了以下修正措施：

Author action:

（1）文件整合与内容统一

我们确认，标注为“Revised Manuscript with Track Changes.docx”的较长版本（60页）是完整、正确且最终版的稿件。该版本包含了您在意见中提到的所有内容，包括监督式MLP基线比较、表9至表12、硬件推理时间分析以及全面修订后的结论。之前提交的较短版本“Manuscript”（53页）是我们第一次修改前的初始版本。“Revised Manuscript with Track Changes.docx”是在“Manuscript”的基础上，根据第一次审稿人修改意见基础上修改增加内容后的版本。这次的第二修改，我们将“Revised Manuscript with Track Changes.docx”命名为Manuscript（2）.docx；将第二次修改后的文件命名为“Revised Manuscript with Track Changes（2）.docx”。

（2）作者列表与通讯邮箱统一

作者列表现已统一为较长版本中的全部七位作者。新增的第七位作者何香牛（HeXiang Niu） 是东北电力大学能源与动力工程学院的科研人员，在本研究的数据处理和模型验证环节做出了重要贡献，其署名是合理且必要的。通讯作者邮箱统一更正为 nhxneepu@163.com，这是王航博士在东北电力大学的官方工作邮箱。此前出现的QQ邮箱为个人备用邮箱，现已全部删除，以避免后续混淆。

（3）图8描述校正

感谢您敏锐地发现了两份文件中对图8奖励轨迹描述的不一致。经重新核查实验原始记录，我们确认图8中展示的奖励曲线起点约为-8，最终收敛至约0.2。较短版本中“从7.5增加到约0.1”的描述属于早期草稿中的笔误，与图中实际数据不符。我们已在统一后的稿件中对此进行了修正，确保文字描述与图表数据完全一致。

现已将全部内容整合为一份完整、统一的“清洁稿件”，不再保留任何不完整或过时的版本命名为“Revised Manuscript with Track Changes（2）.docx”。

________________________________________

Reviewer#1, Concern #2: Within the longer manuscript file, Table 9 (risk values of power shortage by confidence value) and Table 10 (risk values of power shortage under the rainstorm control scheme) share an identical, truncated caption, "Risk values of power shortages by region at different." The captions do not state what varies across rows in each table (confidence level in Table 9, uncertainty source/scheme in Table 10), and the in-text sentence preceding the tables states results are "presented in Tables 8 and 10" while a separate sentence states the confidence-value results are "shown in Table 9," which is confusing on first read. Both captions should be completed and made distinct.

Author response: 非常感谢您再次指出我们在表格标注和正文指引方面的疏漏。表9和表10标题相同且被截断的问题，以及正文中表格引用混乱的现象，确实给您的阅读带来了困扰。这是我们准备稿件时不够细致所致，对此我们深表歉意。我们已对相关问题进行了逐一修正，具体如下：

Author action:

将原表10标题的修正为: High risk proportion of various indicators in Liaoning Power Grid of China in various schemes；将各个表格的顺序重新调整如下所示：

To verify the proposed model and method, three evaluation and calculation schemes are designed and simulated in this section, and the corresponding results are presented in Tables 8 and 10. Among them, the risk of power shortage under different confidence levels is listed in Table 9.

Scheme 1: The proposed models and methods were used to conduct a comparative analysis of the system risk levels under extreme conditions corresponding to different confidence values σ.

Table 8: High-risk proportion of various indicators in various regions of China with different values of in scheme 1

Risk type Confidence value σ/% Proportion of high-risk areas by region /%

Heilongjiang Jilin Liaoning Eastern Inner Mongolia

Power deficiency -20 2.78 0 11.11 0

-10 63.89 26.39 65.28 0

0 65.28 33.33 68.94 0

10 72.37 45.76 75 1.09

20 77.41 50.28 80 3.51

Insufficient spinning reserve -20 11.11 0 80.56 0

-10 81.94 62.5 100 0

0 100 88.89 100 38.89

10 100 100 100 55.56

20 100 100 100 58.06

Voltage violation -20 0 0 11.11 0

-10 62.5 0 68.06 0

0 97.22 12.5 87.5 0

10 98.61 50 98.61 3.09

20 88.89 54.44 100 9.51

Power flow violation -20 0 0 11.11 0

-10 62.5 0 68.06 0

0 98.61 41.67 87.5 0

10 98.61 52.78 100 0

20 98.83 58.78 100 0

Transformer loading violation -20 0 0 11.11 0

-10 62.5 0 68.06 0

0 98.61 38.89 87.5 0

10 98.61 65.28 100 0

20 95.83 72.78 100 0

Frequency violation -20 0 0 4.17 0

-10 21.35 2.78 34.72 0

0 30.56 4.167 41.67 0

10 34.72 11.11 43.67 0

20 38.44 15.11 51.75 2.81

Table 9: Risk values of power shortages by region at different σ

Risk type Confidence value σ Heilongjiang Jilin Liaoning

Power deficiency -20% 1.39×10-4 0 1.5×10-3

-10% 2.77×10-3 0 7.24×10-3

0 1.88×10-3 9.56×10-4 4.8×10-3

10% 5.39×10-3 4.28×10-4 5.77×10-3

20% 6.7×10-3 5.09×10-3 7.55×10-3

Scheme 2:Using historical weather data of a certain rainstorm event as input, and adopting the direct failure model of equipment under rainstorm disasters from reference [38] for the environment-dependent failure probability, while keeping the other models as those proposed in this paper, the system risk was assessed.

Scheme 3: Risk scenario uncertainty models were established by considering only the source–load power fluctuations and only the equipment failures, respectively, and the system risk under partial uncertainty was calculated accordingly.

1) Superiority analysis of the proposed assessment method

Observing Table 8, it can be seen that as the confidence value σ increases, the weather becomes more severe, and the risk values of various indicators also increase. When σ increases to 0, the system is exactly at the critical state of abnormal operation, and the high-risk proportion of each indicator increases significantly, indicating that under such extreme conditions, the system is indeed a high-risk scenario. This further verifies that the extreme weather conditions defined based on the normal operating state threshold of the grid, as proposed in this paper, can indeed provide relatively accurate criteria for risk scenario identification in power grids. Moreover, this method has profound implications: by fitting the functional relationship between meteorological elements of different severity levels and the corresponding system operational risks, the risk level of a planned power grid under future extreme weather can be estimated merely by monitoring meteorological factors. This means that rapid identification of extreme weather scenarios and early warning of risks in power grids become feasible.

2) Accuracy analysis of the proposed assessment method

Extreme rainstorms are not included in the results of risk scenario identification in this paper. Therefore, simulations were carried out using historical data of a rainstorm event, and the results are shown in Table 10. Since no fault modeling of grid equipment under rainstorm weather was performed in this paper, and this case was merely used as a control group in the numerical example, the gridside uncertainty was characterized using the grid equipment fault model for rainstorm disasters from reference [38]. From Table 10, it can be seen that the high-risk proportions of various system indicators obtained by Scheme 1 are significantly higher than those obtained by Scheme 2 by tens of times. This indicates that the two types of extreme weather identified and generated for the Northeast China region in this paper can indeed cause considerable impacts on the power system of this region, whereas the un identified rainstorm poses a relatively low threat to the regional grid. Therefore, the scenario identification method proposed in this paper is effective. By fully considering regional climate and grid characteristics, the proposed method can provide adaptive scenario data for risk assessment of the target power grid, thereby helping to improve the accuracy of each calculated risk value.

Table 10: High risk proportion of various indicators in Liaoning Power Grid of China in various schemes

Scheme Confidence valueσ/% Weather data source Sources of uncertainty Consequence evaluation platform Proportion of high-risk areas by region /%

Source and load Grid side Power deficiency Insufficient spinning reserve Voltage violation Power flow violation Transformer loading violation

1 0 Scenario generation √ √ Planning platform 68.94 100 87.5 87.5 87.5

2 Historical heavy rain √ Reference [30] 6.07 6.41 2.38 2.59 2.81

3 0 Scenario generation √ × 66.88 98.35 79.19 80.13 78.96

× √ 62.74 90.2 86.28 84.44 84

Compared with Scheme 1, the high-risk proportions of various system indicators in Scheme 3 are reduced to varying degrees, with a reduction range of 2% to 9%. This indicates that risk assessment results considering only source load power/grid side failure uncertainties are overly optimistic. It can be seen that comprehensively and accurately characterizing source grid load uncertainties plays an important role in risk assessment work.

________________________________________

Reviewer#1, Concern #3:The accuracy comparison in Table 11 (MAE, RMSE, R2, P95AE for MLP, DQN, PPO, SAC, DDPG, DDPG-Attention) is reported as a single run with a fixed data-partition random seed (2026). The gap between DDPG-Attention and the next-best baseline (DDPG) is 18.6% in MAE and 18.5% in RMSE, and the gap to SAC is smaller still. With no repeated trials across multiple seeds, no confidence intervals, and no significance test, it is not possible to determine whether these margins reflect the attention mechanism itself or run-to-run training variance, which is a standard concern for stochastic RL training curves.

Author response: 非常感谢您指出我们在实验统计严谨性方面的不足。您指出“由于没有跨多个种子的重复试验、没有置信区间、也没有显著性检验，因此无法确定这些差距反映的是注意力机制本身还是运行间训练方差”，这是一个非常关键且专业的问题，切中了深度强化学习实验方法论的核心。我们完全认同您的观点。在深度强化学习中，算法性能对随机种子高度敏感——同一算法在相同超参数下使用不同随机种子运行，其结果可能呈现出显著差异，仅凭单次运行的结果声称某算法优于另一算法，缺乏必要的统计证据支撑。针对您的意见，我们补充了以下实验和统计分析：

Author action:

我们使用10个不同的随机种子（2021–2030），对所有6种算法（MLP、DQN、PPO、SAC、DDPG、DDPG-Attention）分别进行了10次独立训练和评估；采用 Wilcoxon符号秩检验（配对非参数检验）比较DDPG-Attention与次优算法（DDPG）在每个指标上的差异。在6.4.1节、6.4.2节、7 结论部分修改有关多次独立训练后的相关数据，并用红色字体标出：

6.4.1 Comparison with Supervised Learning Baselines

To ensure the statistical robustness of the comparison, all six algorithms (MLP, DQN, PPO, SAC, DDPG, and DDPG-Attention) were re-trained and evaluated 10 times independently using different random seeds (2021–2030). The dataset partitioning for each seed was kept identical across all algorithms to ensure a fair comparison. Table 11 reports the mean ± standard deviation of MAE, RMSE, R², and P95AE on the independent test set. Furthermore, the Wilcoxon signed-rank test was conducted between DDPG-Attention and the second-best performing algorithm (vanilla DDPG) to assess the statistical significance of the observed performance differences, The significance level is set to =0.05. If p<0.05, the null hypothesis is rejected, which demonstrates that the performance improvement brought by the attention mechanism is statistically significant. All error metrics are calculated within the normalized risk indicator space ranging from 0 to 1, with 240 complete 72-hour scenarios adopted as the independent test dataset. The proposed DDPG-Attention achieves the minimum MAE, RMSE and P95AE values as well as the maximum R2, which demonstrates that the feature token-based attention mechanism can improve the fitting accuracy of multiple risk indicators and enhance the control ability against extreme prediction errors.

Table 11: Accuracy and Inference Time Comparison on Independent Test Set

Model MAE RMSE R² P95AE Average Inference Time per Single Scenario(s)

MLP 0.0398 ± 0.0012 0.0500 ± 0.0016 0.923 ± 0.004 0.0981 ± 0.0035 0.041

DQN 0.0560 ± 0.0021 0.0701 ± 0.0025 0.849 ± 0.006 0.1372 ± 0.0051 0.078

PPO 0.0485 ± 0.0018 0.0610 ± 0.0020 0.885 ± 0.005 0.1195 ± 0.0042 0.113

SAC 0.0425 ± 0.0015 0.0531 ± 0.0018 0.913 ± 0.004 0.1040 ± 0.0038 0.105

DDPG 0.0385 ± 0.0011 0.0482 ± 0.0015 0.929 ± 0.004 0.0951 ± 0.0032 0.091

DDPG-Attention 0.0315 ± 0.0009 0.0394 ± 0.0012 0.952 ± 0.003 0.0772 ± 0.0028 0.120

p-value < 0.01 < 0.01 < 0.01 < 0.01 -

As can be observed from Table 11, statistical results from repeated experiments with multiple random seeds demonstrate that the proposed DDPG-Attention outperforms all baseline algorithms in terms of the mean values of the four evaluation metrics. Specifically, regarding overall prediction accuracy, the MAE and RMSE of DDPG-Attention are 0.0315 ± 0.0009 and 0.0394 ± 0.0012, respectively. Compared with the best-performing baseline DDPG (0.0385 ± 0.0011 and 0.0482 ± 0.0015), these metrics are reduced by approximately 18.2% and 18.3%, while they decrease by around 20.9% and 21.2% relative to the supervised learning model MLP (0.0398 ± 0.0012 and 0.0500 ± 0.0016), respectively. The Wilcoxon signed-rank test confirms that these improvements are statistically significant (p-value < 0.01), demonstrating that the performance gains stem from the attention mechanism rather than random training variance. These results reveal that the embedded attention mechanism can effectively capture critical spatio-temporal features strongly correlated with power balance risks, while the reinforcement learning framework further strengthens the model’s capability to characterize the temporal dependencies of risk evolution. It is worth noting that the accuracy comparison in Table 11 is based on open-loop evaluation for all models, where "R" _"t-1" is the ground-truth PSD-BPA label. This protocol is adopted to ensure fair comparison of single-step mapping capability. For practical deployment, the model operates in a closed-loop autoregressive manner; the resulting performance degradation is quantified in Section 6.4.3, and the inference speed remains within the sub-second range.

In terms of goodness-of-fit, DDPG-Attention achieves an R2 of 0.952 ± 0.003, higher than 0.929 ± 0.004 for DDPG and 0.923 ± 0.004 for MLP, indicating that the proposed method possesses a stronger capability to interpret data variations than all comparative models. Particular attention should be paid to the tail error metric P95AE: DDPG-Attention reaches 0.0772 ± 0.0028, which is approximately 18.8% lower than DDPG 0.0951 ± 0.0032 and roughly 21.3% lower than MLP 0.0981 ± 0.0035. This metric quantifies model performance on the 5% extreme samples with the largest prediction errors. The prominent improvement verifies that the proposed method can maintain favorable prediction reliability even under the most severe high-risk scenarios.

The above accuracy comparisons fully validate the superiority of DDPG-Attention for the risk assessment task. The integration of the attention mechanism and reinforcement learning framework statistically improves the overall prediction performance, and more importantly, does not degrade the predictive performance on risk tail events. This carries significant engineering value for power system safety evaluation.

6.4.2 Comparison of Computational Efficiency

(4) Speedup ratio analysis

Based on the above statistical results, the proposed method achieves a speedup ratio of 5.16×106 times compared with the traditional time-sequential Monte Carlo simulation based on PSD-BPA, representing an acceleration of more than six orders of magnitude. Even under an ideal parallel computing condition with 1000-core parallelism and zero communication overhead, the total running time of the traditional method can be reduced to approximately 619.2 seconds, which still yields a speedup ratio of 5160, an improvement of three orders of magnitude.Furthermore, although the single inference time of the proposed DDPG-Attention (0.120 s) increases by approximately 0.08 s compared with MLP (0.041 s), roughly three times that of MLP, the previous accuracy comparisons reveal significant improvements in prediction performance. Under the statistical framework of repeated experiments with multiple random seeds, the MAE of DDPG-Attention is 0.0315 ± 0.0009, approximately 20.9% lower than the 0.0398 ± 0.0012 of MLP; its P95AE reaches 0.0772 ± 0.0028, which is around 21.3% lower than MLP’s 0.0981 ± 0.0035.For power system security assessment, the losses caused by misjudgment and missed judgment of high-risk operating conditions far outweigh the millisecond-level computational delay. This well-balanced trade-off between accuracy and effciency possesses clear practical engineering value.

7 Conclusion

This paper constructs a rapid power balance risk assessment framework under extreme weather conditions by integrating meteorological sensitivity models, time-varying component failure probabilities, DVAE-based coupled scenario generation, time-series labels from PSD-BPA, and an attention-enhanced DDPG estimator. The revised approach explicitly defines the state space, continuous action space, state transition rules, reward function, feature token attention weights, supervised learning baselines, independent test metrics, and timing schemes. The main conclusions are summarized as follows:

(1) The attention mechanism operates on multiple feature tokens instead of a single scalar score. The derived attention matrix is normalized row-wisely, enabling the Actor network to adaptively reweight meteorological data, power source information, grid parameters, load profiles, and risk features from the previous time step along the evolution of the 72-hour scenarios. Under the statistical framework of ten independent runs with random seeds ranging from 2021 to 2030, the MAE, RMSE, R2 and P95AE of DDPG-Attention on the independent test set are 0.0315 ± 0.0009, 0.0394 ± 0.0012, 0.952 ± 0.003 and 0.0772 ± 0.0028, respectively. Compared with vanilla DDPG (0.0385 ± 0.0011, 0.0482 ± 0.0015, 0.929 ± 0.004, 0.0951 ± 0.0032), its MAE and RMSE are reduced by approximately 18.2% and 18.3%, R2 is improved by around 2.5%, and P95AE declines by roughly 18.8%. In comparison with the supervised learning baseline MLP (0.0398 ± 0.0012, 0.0500 ± 0.0016, 0.923 ± 0.004, 0.0981 ± 0.0035), MAE, RMSE and P95AE decrease by about 20.9%, 21.2% and 21.3%, while R2 increases by approximately 3.1%, The Wilcoxon signed-rank test confirms that these improvements are statistically significant (p < 0.01), demonstrating that the performance gains stem from the attention mechanism rather than random training variance. Meanwhile, it exhibits a faster reward convergence rate. It should be noted that the reported accuracy metrics are based on open-loop evaluation using ground-truth previous-step risk labels. In practical closed-loop deployment, the MAE moderately increases to 0.0382 due to error accumulation, yet the inference speed remains within 0.12 s, confirming the method’s engineering applicability.

________________________________________

Reviewer#1, Concern #4 The manuscript compares a sequential MDP formulation (state includes risk features from the previous time step, per Section 5.1) against a feedforward MLP baseline trained by direct regression. The text does not specify whether the MLP receives the same previous-step risk features as the RL agents during evaluation, nor whether the RL agents are evaluated in open-loop (using ground-truth previous-step values) or closed-loop (using their own prior predictions) rollout on the independent test set. This distinction matters for a fair comparison, since closed-loop evaluation typically compounds error over the 72-hour horizon while open-loop evaluation does not.

Author response: 非常感谢您指出我们在评估协议描述方面的疏漏。您提出的关于开环（open-loop）与闭环（closed-loop）评估的问题非常专业且关键，直接关系到MLP基线与本方法之间比较的公平性与可信度。我们对此深表歉意，未能在原稿中清晰阐明这一重要细节。针对您的意见，我们进行了以下澄清和修改：

在原稿中，我们实际上采用了一种公平且严谨的评估协议，但未能在文字中清晰表述。现澄清如下：

（1）评估模式：开环评估（Open-loop Evaluation）

在独立测试集上，所有模型——包括RL智能体（DDPG-Attention、DDPG、SAC、PPO、DQN）和监督学习MLP基线——均采用开环评估方式。具体而言，在每个时间步 t，状态向量中的“前一时刻风险特征” R_(t-1) 使用的是由PSD-BPA计算得出的真实标签值，而非模型自身的先前预测。

（2）公平比较的前提

采用开环评估确保了比较的公平性：

所有模型在相同条件下进行比较——都使用相同的真实 R_(t-1) 值；

比较的仅仅是模型在当前系统状态下的“单步映射能力”，而非模型处理自身误差累积的能力；

MLP与RL智能体在评估时处于完全对等的地位。

（3）开环评估的合理性依据

本研究的核心目标是“快速将当前系统状态映射为风险指标”，相当于用训练好的模型替代耗时的PSD-BPA仿真过程；

开环评估直接度量这一映射关系的准确性，与研究目标一致；

由于PSD-BPA能够为历史数据提供真实标签，在评估阶段使用这些真实值是完全可行且合理的。

（4）训练与部署的区分

需要强调的是：

训练阶段：RL智能体学习过程中，状态转移依赖环境反馈，R_(t-1) 来自历史真实数据（PSD-BPA标签）；

评估阶段（独立测试集）：采用开环评估，R_(t-1) 使用真实标签值；

实际部署阶段（在线推理）：采用闭环自回归推理，R_(t-1) 使用模型自身的预测值。

（5）开环/闭环性能差异的补充说明

闭环推理的误差累积效应是客观存在的。为量化这一影响，我们补充了对比实验：在相同测试集上分别以开环和闭环方式评估DDPG-Attention。结果表明，闭环推理的MAE为0.0382，较开环（0.0315）升高约21%。尽管存在性能下降，闭环推理0.120 s的推理速度和0.0382的MAE仍能满足实际工程对时效性和精度的基本需求。该结果已在修订稿第6.4.1节末尾补充说明。

Author action:

（1）第5.1节（MDP建模）末尾新增评估协议说明

Evaluation Protocol: During the evaluation phase on the independent test set, all models—including the RL agents and the MLP baseline—are evaluated in an open-loop manner. Specifically, the ‘previous-step risk features’ "R" _"t-1" ^"label" provided in the state vector are the ground-truth labels calculated by PSD-BPA, rather than the models’ own prior predictions. This protocol ensures a fair comparison by evaluating each model’s capability to map the current system state to risk indicators without the confounding effect of temporal error accumulation. In actual online deployment, however, closed-loop autoregressive inference is adopted, where "R" _"t-1" is recursively fed from the model’s own predictions.

（2）第5.3节（MLP基线）中明确说明输入一致性

To prevent temporal information leakage, the dataset must be split into mutually exclusive training, validation and test sets on the basis of complete 72-hour scenarios rather than individual hourly samples. The exact number of scenarios in each subset and the random seed are reported for reproducibility. The MLP baseline is trained via direct regression with the same normalized input features as the RL agents, including the ground-truth risk features from the previous time step during training. During evaluation, the MLP also receives the same open-loop ground-truth〖" R" 〗_"t-1" ^"label" as the RL agents, ensuring an equitable comparison.

（3）第6.4.1节（精度比较）中补充说明

As can be observed from Table 11, statistical results from repeated experiments with multiple random seeds demonstrate that the proposed DDPG-Attention outperforms all baseline algorithms in terms of the mean values of the four evaluation metrics. Specifically, regarding overall prediction accuracy, the MAE and RMSE of DDPG-Attention are 0.0315 ± 0.0009 and 0.0394 ± 0.0012, respectively. Compared with the best-performing baseline DDPG (0.0385 ± 0.0011 and 0.0482 ± 0.0015), these metrics are reduced by approximately 18.2% and 18.3%, while they decrease by around 20.9% and 21.2% relative to the supervised learning model MLP (0.0398 ± 0.0012 and 0.0500 ± 0.0016). These results reveal that the embedded attention mechanism can effectively capture critical spatio-temporal features strongly correlated with power balance risks, while the reinforcement learning framework further strengthens the model’s capability to characterize the temporal dependencies of risk evolution. It is worth noting that the accuracy comparison in Table 11 is based on open-loop evaluation for all models, where "R" _"t-1" is the ground-truth PSD-BPA label. This protocol is adopted to ensure fair comparison of single-step mapping capability. For practical deployment, the model operates in a closed-loop autoregressive manner; the resulting performance degradation is quantified in Section 6.4.3, and the inference speed remains within the sub-second range.

（4）新增第6.4.3节（开环与闭环性能对比）

6.4.3 Open-loop vs. Closed-loop Performance Analysis

To quantify the impact of temporal error accumulation in closed-loop inference, we additionally evaluate DDPG-Attention in both open-loop and closed-loop modes on the same independent test set. In closed-loop mode, the state at time step t uses the model’s own prediction from time step "t-1" as "R" _"t-1" . The results are shown in Table 13.

Table 13: Comparison of relevant data under open-loop and closed-loop modes

Evaluation Mode MAE RMSE R² P95AE

Open-loop 0.0315 ± 0.0009 0.0394 ± 0.0012 0.952 ± 0.003 0.0772 ± 0.0028

Closed-loop 0.0382 ± 0.0015 0.0476 ± 0.0020 0.926 ± 0.005 0.0935 ± 0.0038

The closed-loop MAE is approximately 21% higher than the open-loop MAE, attributable to the compounding effect of prediction errors over the 72-hour horizon. Nevertheless, the closed-loop MAE of 0.0382 remains comparable to the open-loop performance of vanilla DDPG (0.0385), and the inference speed of 0.120s comfortably satisfies the sub-second requirement for online deployment. This demonstrates that the proposed method maintains practical utility even under the more challenging closed-loop inference condition.

（5）第7节（结论）中补充说明

(1) The attention mechanism operates on multiple feature tokens instead of a single scalar score. The derived attention matrix is normalized row-wisely, enabling the Actor network to adaptively reweight meteorological data, power source information, grid parameters, load profiles, and risk features from the previous time step along the evolution of the 72-hour scenarios. Under the statistical framework of ten independent runs with random seeds ranging from 2021 to 2030, the MAE, RMSE, R2 and P95AE of DDPG-Attention on the independent test set are 0.0315 ± 0.0009, 0.0394 ± 0.0012, 0.952 ± 0.003 and 0.0772 ± 0.0028, respectively. Compared with vanilla DDPG (0.0385 ± 0.0011, 0.0482 ± 0.0015, 0.929 ± 0.004, 0.0951 ± 0.0032), its MAE and RMSE are reduced by approximately 18.2% and 18.3%, R2 is improved by around 2.5%, and P95AE declines by roughly 18.8%. In comparison with the supervised learning baseline MLP (0.0398 ± 0.0012, 0.0500 ± 0.0016, 0.923 ± 0.004, 0.0981 ± 0.0035), MAE, RMSE and P95AE decrease by about 20.9%, 21.2% and 21.3%, while R2 increases by approximately 3.1%, The Wilcoxon signed-rank test confirms that these improvements are statistically significant (p < 0.01), demonstrating that the performance gains stem from the attention mechanism rather than random training variance. Meanwhile, it exhibits a faster reward convergence rate. It should be noted that the reported accuracy metrics are based on open-loop evaluation using ground-truth previous-step risk labels. In practical closed-loop deployment, the MAE moderately increases to 0.0382 due to error accumulation, yet the inference speed remains within 0.12 s, confirming the method’s engineering applicability.

________________________________________

Reviewer#1, Concern #5 The authors are encouraged to discuss the following references, briefly acknowledging what these works accomplished and the results they achieved, so as to better position the present study within the existing literature.

Author response: 非常感谢您建议我们讨论两篇参考文献，以更好地将本研究定位在现有文献中。我们认真阅读了您推荐的文献，并按照您的建议在引言部分增加了对这两项工作的简要讨论。

文献[1]（Feng et al.）针对高比例可再生能源接入导致的系统惯量下降问题，提出了一种虚拟电厂（VPP）聚合模型和市场机制，使分布式能源能够参与惯性和一次频率响应。该方法在IEEE 30节点和118节点系统的测试中，对频率最低点和准稳态频率的拟合平均绝对百分比误差不超过0.03%，系统总成本降低约40%，净利润增加约30%。该研究与我们关注的问题具有场景关联性——都聚焦于高比例可再生能源接入带来的系统稳定性挑战，但文献[1]侧重于频率稳定与市场机制设计，而本文关注功率平衡风险。

文献[2]（Meng et al.）针对双侧供电铁路系统中车辆运行点频繁变化导致的稳定性问题，提出了一种可解释的残差前馈神经网络（ResFNN）结合SHAP方法，用于高效、准确地辨识列车变流器的“黑箱”阻抗模型，评估系统在低频频段和高频频段的稳定性。该研究在方法论层面与本文具有关联性——都利用了神经网络来处理传统方法难以解决的复杂系统建模与辨识问题。

在正文中，我们已将上述讨论插入到引言第三段末尾（即“现有研究不足”与“本文方法概述”之间的过渡位置），并以此引出本文的研究动机和创新点。具体修改内容如下：

Author action:

在引言第三段后加入相关内容：

1 Introduction

With the in depth advancement of the “dual carbon” goals, the construction of new power systems has entered a critical stage, and the share of renewable energy installations, represented by wind power and photovoltaics (PV), continues to increase[1-2]. However, the strong intermittency and volatility of renewable energy output make the system operation significantly more dependent on meteorological conditions. Unlike highly destructive extreme weather events such as typhoons and thunderstorms, persistent stagnant weather does not directly damage the physical structure of the power grid, but its long duration and wide impact range can easily lead to renewable energy being in a state of low or even zero output for an extended period, thereby triggering long term power supply demand imbalance risks in the power system. Events such as high temperature droughts and multiple days without wind or sunlight that have occurred in many regions worldwide have fully demonstrated that weather factors are becoming an important unknown risk source threatening the security of power supply in new power systems[3-4]. Therefore, studying the supply demand imbalance risk of power systems under extreme weather and developing fast and accurate risk assessment methods are of significant theoretical importance and engineering value.

Currently, domestic and international scholars have conducted extensive research on power system risk assessment under extreme weather. In terms of source load characteristic modeling, references[5-6] proposed renewable energy station output prediction methods considering factors such as wind speed, temperature, humidity, and solar irradiance. Reference[7] separated climate sensitive load from total load by analyzing the spatiotemporal correlation between climate factors and power load. Reference [8] studied the impact of high temperature and drought weather on the output of hydropower and thermal power, and proposed elasticity indicators at the power source level. In the field of system resilience assessment, existing studies have proposed static resilience indicators based on resource adequacy and component redundancy[9-10], as well as dynamic resilience indicators established according to the recovery process during and after extreme events. Regarding scenario generation, reference [11] proposed a weather scenario construction method based on extreme value theory, but it lacked specific scenario modeling and case study validation. References [12-13] explored the emergency control strategies of power systems and the optimal configuration of distributed generation and energy storage devices under different scenarios.

However, existing research still has obvious limitations in coping with supply demand imbalance risks of power systems under extreme weather. On the one hand, most studies tend to analyze sudden extreme weather events and persistent stagnant weather separately, failing to uniformly reveal the differentiated impact mechanisms of the two types of extreme weather from the three aspects of supply, transmission, and demand. On the other hand, at the uncertainty modeling level, source side output fluctuations, grid side equipment failures, and load side climate sensitive responses are often treated in isolation, and a comprehensive quantification of their coupled uncertainties is lacking. In particular, the time varying characteristics of equipment failure probability, which are jointly affected by service life, environmental conditions, and operating status, are insufficiently considered in existing risk assessment models. More critically, although traditional time series production simulation or Monte Carlo methods can obtain relatively accurate risk results, their huge computational overhead makes it difficult to meet the timeliness requirements for rapid risk identification during the evolution of extreme weather. Moreover, existing resilience assessment studies mostly focus on short term emergency power supply after the destruction of grid topology by extreme weather, while the exploration of rapid assessment methods for global and persistent supply demand imbalance risks remains insufficient. The above deficiencies indicate that there is an urgent need to develop an intelligent method that can efficiently handle high dimensional state spaces, adaptively focus on key risk features, and combine offline training with online rapid assessment. Recently, data-driven methods have also been explored to address complex challenges in power and energy systems. Feng et al. [41] proposed a virtual power plant aggregation model and market mechanism that enables distributed energy resources to provide inertia and primary frequency response, achieving high modeling accuracy with mean absolute percentage errors below 0.03% in frequency response fitting and approximately 40% reduction in system operation costs. Separately, Meng et al. [42] developed an interpretable neural network for impedance identification in double-sided feeding railway systems, combining residual feedforward neural networks with SHAP analysis to effectively assess stability under variable operating conditions. While these studies demonstrate the potential of learning-based methods for system modeling and stability analysis, they do not directly address the problem of rapid, sequential risk assessment for power balance under extreme weather events—particularly the coupled uncertainties from source, grid, and load sides.

C. Feng, Huang Z, Lin J, et al. Aggregation model and market mechanism for virtual power plant participation in inertia and primary frequency response[J]. IEEE Transactions on Power Systems, 2025.

Meng X, Hu G, Liu Z, et al. Neural network-based impedance identification and stability analysis for double-sided feeding railway systems[J]. IEEE Transactions on Transportation Electrification, 2024, 11(1): 4429-4442.

Attachment Submitted filename: Response_to_Reviewers_auresp_2.docx

# Peer review: Decision Letter 2

**DOI:** 10.1371/journal.pone.0359101.r005

**Authors:** Zhengmao Li

9 Sep 2026

<p>A Fast Risk Assessment Method for Power Balance Under Extreme Weather Conditions Based on Deep Reinforcement Learning with a Coupled Attention Mechanism

PONE-D-26-32244R2

Dear Dr. Hang,

We’re pleased to inform you that your manuscript has been judged scientifically suitable for publication and will be formally accepted for publication once it meets all outstanding technical requirements.

Within one week, you’ll receive an e-mail detailing the required amendments. When these have been addressed, you’ll receive a formal acceptance letter and your manuscript will be scheduled for publication.

An invoice will be generated when your article is formally accepted. Please note, if your institution has a publishing partnership with PLOS and your article meets the relevant criteria, all or part of your publication costs will be covered. Please make sure your user information is up-to-date by logging into Editorial Manager at Editorial Manager® and clicking the ‘Update My Information' link at the top of the page. For questions related to billing, please contact billing support.

If your institution or institutions have a press office, please notify them about your upcoming paper to help maximize its impact. If they’ll be preparing press materials, please inform our press team as soon as possible -- no later than 48 hours after receiving the formal acceptance. Your manuscript will remain under strict press embargo until 2 pm Eastern Time on the date of publication. For more information, please contact onepress@plos.org.

Kind regards,

Zhengmao Li

Academic Editor

PLOS One

Additional Editor Comments (optional):

Reviewers' comments:

Reviewer's Responses to Questions

**Comments to the Author**

Reviewer #1: (No Response)

Reviewer #2: All comments have been addressed

**********

2. Is the manuscript technically sound, and do the data support the conclusions?

Reviewer #1: (No Response)

Reviewer #2: Yes

**********

3. Has the statistical analysis been performed appropriately and rigorously?

Reviewer #1: (No Response)

Reviewer #2: Yes

**********

4. Have the authors made all data underlying the findings in their manuscript fully available?

The PLOS Data policy

Reviewer #1: (No Response)

Reviewer #2: Yes

**********

5. Is the manuscript presented in an intelligible fashion and written in standard English?

Reviewer #1: (No Response)

Reviewer #2: Yes

**********

Reviewer #1: (No Response)

Reviewer #2: The authors have adequately addressed the comments raised in the previous review rounds and have substantially improved the manuscript. The methodology, experimental design, statistical analysis, and evaluation protocol are now presented more clearly and rigorously. The additional experiments further strengthen the reliability of the results. Overall, my main concerns have been satisfactorily resolved, and I consider the manuscript suitable for publication in its current form.

**********

what does this mean?). If published, this will include your full peer review and any attached files.

If you choose “no”, your identity will remain anonymous but your review may still be made public.

**Do you want your identity to be public for this peer review?**  For information about this choice, including consent withdrawal, please see our Privacy Policy

Reviewer #1: No

Reviewer #2: No

**********

# Editor report: Acceptance letter

**DOI:** 10.1371/journal.pone.0359101.r006

**Authors:** Zhengmao Li

PONE-D-26-32244R2

PLOS One

Dear Dr. Hang,

I'm pleased to inform you that your manuscript has been deemed suitable for publication in PLOS One. Congratulations! Your manuscript is now being handed over to our production team.

At this stage, our production department will prepare your paper for publication. This includes ensuring the following:

* All references, tables, and figures are properly cited

* All relevant supporting information is included in the manuscript submission,

* There are no issues that prevent the paper from being properly typeset

You will receive further instructions from the production team, including instructions on how to review your proof when it is ready. Please keep in mind that we are working through a large volume of accepted articles, so please give us a few days to review your paper and let you know the next and final steps.

Lastly, if your institution or institutions have a press office, please let them know about your upcoming paper now to help maximize its impact. If they'll be preparing press materials, please inform our press team within the next 48 hours. Your manuscript will remain under strict press embargo until 2 pm Eastern Time on the date of publication. For more information, please contact onepress@plos.org.

You will receive an invoice from PLOS for your publication fee after your manuscript has reached the completed accept phase. If you receive an email requesting payment before acceptance or for any other service, this may be a phishing scheme. Learn how to identify phishing emails and protect your accounts at https://explore.plos.org/phishing.

If we can help with anything else, please email us at customercare@plos.org.

Thank you for submitting your work to PLOS One and supporting open access.

Kind regards,

PLOS One Editorial Office Staff

on behalf of

Dr Zhengmao Li

Academic Editor

PLOS One
